# An amygdalar neural ensemble that encodes the unpleasantness of pain

**DOI:** 10.1126/science.aap8586

**Published:** 2018-01-18

**Authors:** Gregory Corder, Biafra Ahanonu, Benjamin F. Grewe, Dong Wang, Mark J. Schnitzer, Grégory Scherrer

**Affiliations:** 1 Department of Anesthesiology, Perioperative, and Pain Medicine, Stanford University School of Medicine, Stanford, CA 94305, USA; 2 Department of Molecular and Cellular Physiology, Stanford University School of Medicine, Stanford, CA 94305, USA; 3 Department of Neurosurgery, Stanford University School of Medicine, Stanford, CA 94305, USA; 4 Stanford Neurosciences Institute, Stanford University, Stanford, CA 94305, USA; 5 Department of Biology, Stanford University, Stanford, CA 94305, USA; 6 Howard Hughes Medical Institute, Stanford University, Stanford, CA 94305,USA; 7 CNC Program, Stanford University, Stanford, CA 94305, USA; 8 Department of Applied Physics, Stanford University, Stanford, CA 94305, USA; 9 New York Stem Cell Foundation—Robertson Investigator, Stanford University, Stanford, CA 94305, USA; * These authors contributed equally to this work; † Present address: Department of Psychiatry and Department of Neuroscience, Perelman School of Medicine, University of Pennsylvania, Philadelphia, PA 19104, USA; ‡ Present address: Institute of Neuroinformatics, ETH and University of Zurich, Zurich 8057, Switzerland

## Abstract

Pain is an unpleasant experience. How the brain’s affective neural circuits attribute this aversive quality to nociceptive information remains unknown. By means of time-lapse in vivo calcium imaging and neural activity manipulation in freely behaving mice encountering noxious stimuli, we identified a distinct neural ensemble in the basolateral amygdala that encodes the negative affective valence of pain. Silencing this nociceptive ensemble alleviated pain affective-motivational behaviors without altering the detection of noxious stimuli, withdrawal reflexes, anxiety, or reward. Following peripheral nerve injury, innocuous stimuli activated this nociceptive ensemble to drive dysfunctional perceptual changes associated with neuropathic pain, including pain aversion to light touch (allodynia). These results identify the amygdalar representations of noxious stimuli that are functionally required for the negative affective qualities of acute and chronic pain perception.

Pain is both a sensory and affective experience (*1*). The unpleasant percept that dominates the affective dimension of pain is coupled with the motivational drive to engage protective behaviors that limit exposure to noxious stimuli (*2*). Although previous work has uncovered detailed mechanisms underlying the sensory detection of noxious stimuli and spinal processing of nociceptive information (*3*), how brain circuits transform emotionally inert information ascending fromthe spinal cord into an affective pain percept remains unclear (*4*). Attaining a better understanding of the mechanisms underlying pain affect is important, because it could lead to novel therapeutic strategies to limit the suffering of chronic pain patients.

The amygdala critically contributes to the emotional and autonomic responses associated with valence coding of neural information, such as responses during fear or pain (*5*). Damage to the basolateral amygdala (BLA) can induce a rare phenomenon in which noxious stimuli remain detected and discriminated but are devoid of perceived unpleasantness and do not motivate avoidance (*6**,* *7*). Conversely, impairment of somatosensory cortex function reduces the ability to both localize noxious stimuli and describe their intensity, without altering aversion or avoidance (*8**,* *9*). Thus, BLA affective neural circuits might link nociceptive inputs to aversive perceptions and behavior selection.

Patients with chronic pain often suffer allodynia, a pathological state in which an intense unpleasant percept arises in response to innocuous stimuli such as light touch (*10*). Notably, the BLA displays heightened activity during chronic pain (*11*), and longitudinal functionalmagnetic resonance imaging studies in humans and rodents show that neural hyperactivity and altered functional connectivity in the amygdala parallel the onset of chronic pain, suggesting that the BLA might play a critical role in shaping pathological pain perceptions (*12*–*14*). However, it remains unclear how the BLA influences the unpleasant aspects of innate acute and chronic pain perceptions (*15*), while the role of nociceptive circuits in the central amygdala are better understood (*16**,* *17*). Previous studies attempting to define pain affect mechanisms recorded the acute nociceptive responses of single amygdalar neurons in anesthetized animals (*11**,* *18*). However, recent work has shown that the BLA encodes information via the coordinated dynamics of neurons within large ensembles (*19*); it is therefore important to resolve how the BLA processes pain affect at the neural ensemble level in awake, freely behaving animals.

We first performed fluorescence in situ hybridization studies and used the immediateearly gene marker of neural activity, c-Fos, to determine that c-Fos^+^ neurons activated by nociceptive stimuli comprised a population of mid-anterior BLA Camk2a^+^ principal neurons (fig. S1). To identify how the BLA encodes nociceptive information, we used a head-mounted miniature microscope to track the somatic Ca^2+^ dynamics of individual BLA Camk2a^+^ principal neurons in freely behaving mice presented with diverse noxious and innocuous stimuli (Fig. 1, A to D, and figs. S2 and S3) (*20*). We monitored pain-related behaviors by measuring each animal’s locomotor acceleration, which allowed us to track both reflexive withdrawal and affectivemotivational behaviors that include attendance to the stimulated tissue and escape (Fig. 1, A and E, and fig. S4).

**Fig. 1:**
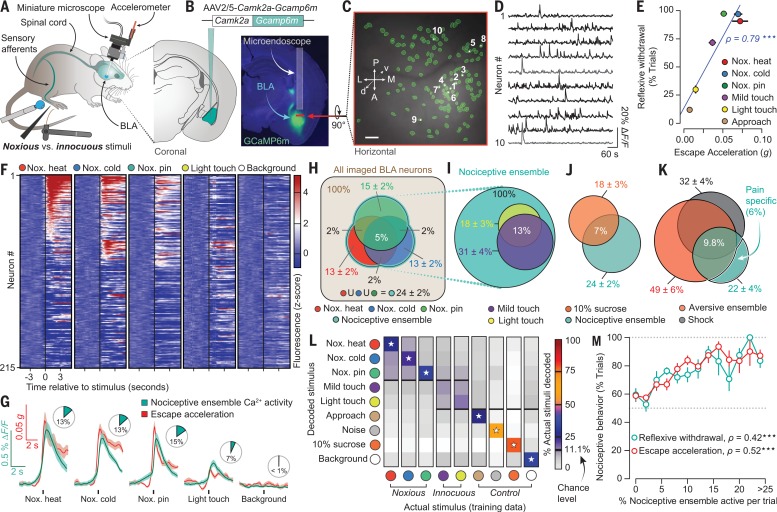
**A distinct nociceptive neural ensemble in the BLA represents diverse painful stimuli.** (**A**) BLA neural activity was imaged in freely behaving mice with a microendoscope and the virally expressed fluorescent Ca^2+^ indicator GCaMP6m. Noxious mechanical (pin prick) and thermal (55°C H_2_O and 5°C H_2_O or acetone) stimuli were delivered to the left hindpaw, while reflexive and affective-motivational behavior were monitored via a scope-mounted accelerometer. (**B**) Microendoscope placement and GCaMP6m expression in the right, contralateral BLA. The red line marks the focal plane and is also a 1.0-mm scale bar. (**C** and **D**) Map of active BLA neurons (n = 131 neurons) with numbers in (C) matching independent component analysis–derived neuron activity traces in (D). Scale bar, 100 µm. (**E**) Spearman’s correlation between reflexive withdrawal and affective-motivational escape acceleration. (**F**) Mean Ca2^+^ response (*Z*-scored Δ*F/F* per trial) across all trials for all BLA neurons imaged during a single session (n = 215 neurons) from the same animal. Neurons are aligned from high to low Ca^2+^ responses in the noxious heat trials. Individual neuron identifications between different stimuli are consistent across the trial rows. (**G**) Stimulus-locked mean Ca^2+^ activity within the nociceptive ensemble (cyan) and mean affective-motivational escape acceleration (red). Shaded region, ±SEM. Pie charts indicate the percentages of significantly responding neurons. (**H**) Venn diagram of neural populations encoding nociceptive information in response to noxious heat, cold, and pin stimuli. Numbers show means ± SEM of percentages of significantly responding neurons across imaging sessions (see fig. S5E). (**I**) Neural populations within the nociceptive ensemble that encode innocuous light touch (0.07-g filament) and mild touch (a 1.4- or 2.0-g filament). (**J**) Divergent neural populations (versus the nociceptive ensemble) encoding appetitive stimuli (10% sucrose consumption). (**K**) Overlapping BLA populations between the nociceptive ensemble, electric footshock, and aversive stimuli (isopentylamine odor, facial air puff, 85-dB noise, and quinine consumption). A subset of nociceptive ensemble neurons were pain specific (~6% of the BLA neurons). (**L**) Accuracies of a nine-way Naïve Bayes decoder that distinguishes the ensemble activities for noxious, innocuous, aversive, anticipatory, and appetitive stimuli. The percentage of decoder accuracy to output for the actual stimuli (diagonal) was compared to that for the incorrect stimuli (off the diagonal) and normalized so that each actual stimuli column added up to 100%. Stars on the diagonal indicate the correct prediction of said stimulus was significantly greater than all off-diagonal stimuli within the same column (Wilcoxon sign-rank, Benjamini-Hochberg corrected). (**M**) Spearman’s correlation (ρ) between per trial pain behavioral responses and nociceptive ensemble activation. Error bars, ±SEM per session animal responses; *n* = 9 mice, 3 to 4 sessions each.

Noxious heat, cold, and pin prick stimuli elicited significant Ca^2+^ responses in 15 ± 2% (SEM), 13 ± 2%, and 13 ± 2% of active BLA neurons, respectively [3397 neurons (117 ± 8 neurons per session)] (Fig. 1, F to H, and table S1). Innocuous light touch induced Ca^2+^ activity in a smaller subset of neurons (7 ± 1%) (Fig. 1, F and I, and fig. S5E). Alignment of all stimulusevoked ensemble responses to the noxious heat trials revealed an overlapping population of principal neurons that encoded nociceptive information across pain modalities (i.e., noxious heat, cold, pin), which we refer to here as the BLA nociceptive ensemble (24 ± 2% of active BLA neurons) (Fig. 1, F to I).

This ensemble was composed of multimodal responsive neurons, as well as a unique population that appeared to encode nociception selectively and no other sensory information (6 ± 1% of all imaged neurons) (Fig. 1K and fig. S5G). Pain behavioral responses evoked by noxious stimuli closely mirrored the activity of this nociceptive neural ensemble (Fig. 1, E and G, and fig. S4, D and E). The nociceptive ensemble contained a subset of neurons that maintained their noxious stimulus response properties for more than a week (11% of 3223 cross-day–aligned neurons) (fig. S6). Increasingly salient stimuli, from light touch (18 ± 3% of the nociceptive ensemble) to mild touch (31 ± 4%), activated larger subsets of the nociceptive ensemble (Fig. 1, G and I, fig. S5, D and E, and table S1) and induced heightened behavior (Fig. 1E and fig. S4). Expectation of stimulus contact (“approach/no contact” trials) also evoked sparse BLA activity (7 ± 2% of the total population) (fig. S5, A to E, and table S1). BLA activity did not correlate with exploratory locomotion (fig. S7, A to E) (*21*).

To determine whether the BLA nociceptive ensemble broadly encodes stimulus valence (*22**,* *23*), we presented mice with an appetitive stimulus (10% sucrose). Sucrose consumption was encoded by a distinct ensemble (18 ± 3% of all neurons) that only overlapped with a subset of neurons in the nociceptive ensemble (7% of total neurons) (Fig. 1J and fig. S5E) (*19*). Similar to conditioned responsive valence networks (*23*), neurons encoding unconditioned nociceptive and appetitive information were spatially intermingled (fig. S5, F, H, and I). Consistent with these results, nociceptive c-Fos^+^ Neuronsexpressed the negative valence marker gene Rspo2 but not the positive valence marker gene *Pppr*1b (24) (fig. S1, D and E).

We next determined if the nociceptive ensemble was engaged during aversive experiences other than pain by presenting a panel of sensory, but nonsomatosensory or nonnaturalistic, aversive stimuli, including repulsive odor, bitter taste, loud tone, facial air puff, andelectric shock. We found that while there was overlap between the neural ensembles that encode nociceptive, aversive, and electric shock stimuli (~10% of all imaged neurons), there remained a subset of BLA neurons (~6% of imaged neurons) that responded only to naturalistic nociceptive stimuli (Fig. 1K and fig. S8).

By analyzing the neural ensemble dynamics with pattern classification methods, we were able to classify and distinguish with high accuracy noxious stimuli from other aversive stimuli (fig. S8E), supporting the finding that noxious stimuli induce a distinct mode of BLA activation (supplementary text S1). Moreover, sensory stimuli of different valences, intensities, and modalities are represented by unique activity codes. Noxious stimuli were encoded distinctly from one another and could be distinguished with even higher fidelity from innocuous, nonnociceptive aversive, and appetitive stimuli (Fig. 1L and fig. S9, A and B), indicating that there is a core set of BLA neurons that encodes nociceptive stimuli via specific dynamic neural codes. One crucial finding was that greater activation of this BLA nociceptive ensemble was predictive of increased pain behaviors, suggesting that BLA nociceptive processing influences the magnitude of pain behaviors (Fig. 1M and fig. S7, H and I).

To test the causal role of the BLA nociceptive ensemble for pain behaviors, we expressed a Cre-dependent inhibitory DREADD neuromodulator (hM4-mCherry) in mutant TRAP mice (Fos^CreERT2^) by applying noxious pin pricks that induced activity-dependent, spatially, and temporally controlled DNA recombination and hM4- mCherry expression (noci-TRAP^hM4^ mice) (Fig. 2, A to C, and fig. S10) (*25**,* *26*). Since the BLA encodes multiple modalities of nociceptive stimuli within a core ensemble (Fig. 1H), we hypothesized that silencing the neurons activated by noxious pin prickwould alter behavioral responses to all types of noxious stimuli. Indeed, the hM4 agonist clozapine-N-oxide (CNO; 10 mg/kg) significantly reduced both attending and escape behaviors, but not stimulus detection and withdrawal, for both mechanical and thermal noxious stimuli (Fig. 2, D to G, and fig. S11, A and B). CNO alone had no effect on pain behaviors in control mice (fig. S11C) (27). To test operant pain behavior, we next allowed noci-TRAP^hM4^ mice to explore a thermal gradient track in which the polar ends were set at noxious cold (5 to 17°C) and hot (42 to 48°C) temperatures (Fig. 2H). The noci-TRAP^hM4^ mice injected with control saline rapidly acquired an adaptive avoidance strategy of the noxious zones. In contrast, noci-TRAP^hM4^ mice treated with CNO visited the noxious zones more frequently and for prolonged periods (Fig. 2, H to J, and fig. S12). Similarly, inhibition of the BLA nociceptive ensemble eliminated pain affectivemotivational behaviors induced by the optogenetic activation of peripheral primary afferent nociceptors (fig. S13).

**Fig. 2:**
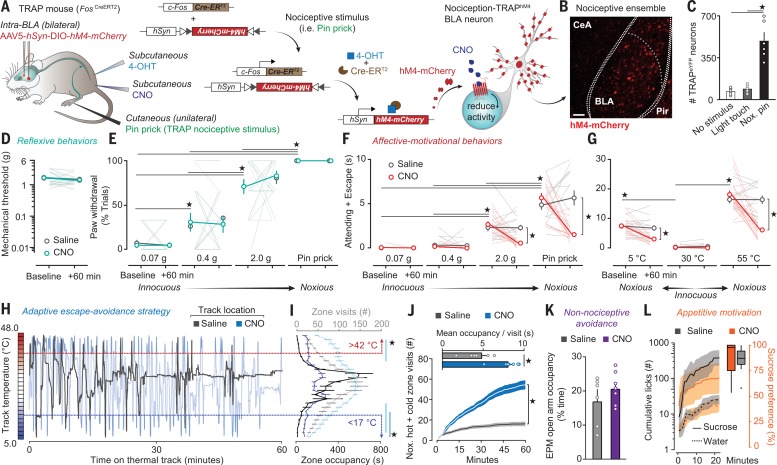
**The BLA nociceptive ensemble is necessary for generating protective and avoidance behavioral responses to painful stimuli.** (**A**) Experimental strategy for inhibiting BLA nociceptive ensemble activity. Nociception-mediated targeted recombination in activity neural populations (noci-TRAP) of the inhibitory DREADD(hM4) receptor. CNO, clozapine N-oxide; 4-OHT, 4-hydroxytamoxifen. (**B**) noci-TRAP^hM4^ expression in the BLA nociceptive ensemble. CeA, central amygdala; ITC, intercalated neurons; Pir, piriform cortex. Scale bar, 50 µm. (**C**) Quantification of BLA noci-TRAPeYFP neurons following either no stimulus, innocuous touch (0.07-g filament), or noxious pin prick stimulation; n = 6mice/group. (**D** and **E**) Effect of inhibiting the BLA nociceptive ensemble against reflexive behaviors, demonstrated by a von Frey mechanical threshold assay (D) and reflexive withdrawal frequency to increasing noxious mechanical stimuli (E). n = 14 mice per group. (**F** and **G**) Effect of inhibiting the BLA nociceptive ensemble against pain affective-motivational behaviors in response to increasingly noxious mechanical (F) and thermal stimuli (G). *n* = 14 mice per group. (H) Effect of inhibiting the BLA nociceptive ensemble on adaptive avoidance behavior to noxious thermal environments.The kymograph displays mouse location on a thermal gradient track over a 60-min trial following administration of saline (*n* = 6 mice) or CNO (*n* = 7 mice). Noxious temperature zones were areas at <17°C and >42°C. (**I**) Total number of visit entries (gray and light blue lines) and the occupancy time (black and dark blue lines) in the track’s 25 thermal zones. (**J**) Temporal cumulative visits and the mean occupancy time per visit (inset) to the noxious hot and cold zones. (**K**) Occupancy time within the open arms of an elevated plus maze (EPM). (**L**) The 10% sucrose spout lick rates and preference over a water choice. Overlaid dots and lines represent individual animals. Error bars, ± SEM. For (C) and (E) to (G) (CNO group baseline time points only), one-way analysis of variance (ANOVA; Friedman’s) plus Dunn’s correction. For (D) to (G) and (I), two-way repeated measures ANOVA with Bonferroni correction. For (J) and (K), data on left analyzed with Kolmogorov-Smirnov test; data on right analyzed with Student’s t test. Star, *P* < 0.05.

Whether pain and anxiety rely on common or distinct BLA ensembles is unknown; therefore, we placed noci-TRAP^hM4^ mice within an elevated plus maze, in which anxiety drives avoidance of the open arms (Fig. 2K). The noci-TRAP^hM4^ mice given either saline or CNO displayed equivalent visits to and occupancy of the open arms (fig. S14, A and B). Since nociceptive and sucrose reward-related information were encoded in divergent networks (Fig. 1J), we tested the contribution of the nociceptive ensemble to appetitive motivational drive during sucrose preference training. CNO enhanced sucrose reward in sucrose-naïve conditions (28) but had no retarding effects on preference development or on lick rates, relative to controls (Fig. 2L and fig. S14C). Thus, this BLA nociceptive ensemble transforms emotionally inert nociceptive information into an affective signal that is necessary for the selection and learning of motivational protective pain behaviors.

We next investigated the contribution of BLA neural ensemble activity to chronic pain. A hallmark of chronic neuropathic pain is the appearance of allodynia and hyperalgesia, both pathological perceptual states in which aversion is ascribed to innocuous somatosensory stimuli and exacerbated in response to noxious stimuli, respectively (Fig. 3A) (*29*). We hypothesized that this pathological perceptual switch might result from maladaptive transformations in BLA coding. We tracked the longitudinal dynamics of BLA ensembles before and after the development of neuropathic pain induced by sciatic nerve injury (17,396 neurons, n = 17mice) (Fig. 3). Throughout the development of chronic neuropathic pain, a subset of neurons stably encoded the nociceptive ensemble for both noxious mechanical and cold stimuli (fig. S6). Nerve injury did not significantly increase the spontaneous activity of the nociceptive ensemble and overall BLA population (fig. S15, A and B). However, BLA neural activity elicited in response to light touch displayed a significant expansion within the nociceptive ensemble in neuropathic (291 ± 88% increase) but not in uninjured mice (38 ± 14% decrease) (Fig. 3, D to G, and fig. S15, C to E). The emergence of this neuropathic coding schema was accompanied by the development of reflexive paw withdrawal hypersensitivity and by enhanced affective-motivational pain behaviors (Fig. 3, B and C, and fig. S4, C to F). The magnitudes of the behavioral responses and the BLA nociceptive ensemble Ca^2+^ activity were significantly correlated before and after injury (Fig. 3H and fig. S15F). These results suggest a role for the BLA in the emergence of allodynia in chronic pain states.

**Fig. 3:**
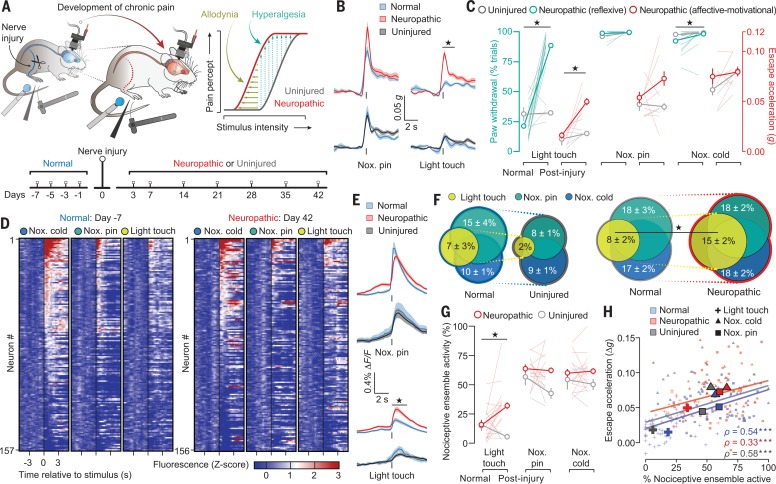
**Convergence of BLA neural ensemble representations of innocuous and noxious information during chronic pain.** (**A**) Longterm tracking of BLA neural activity with microendoscopes throughout the development of chronic neuropathic pain. Peripheral nerve injury results in an increased sensitivity and perceived aversion to innocuous (allodynia) and noxious (hyperalgesia) stimuli. (**B**) Affective-motivational escape acceleration for neuropathic (top row; n = 5 mice) and uninjured (bottom row; n = 4 mice) animals in response to noxious pin or light touch stimuli before and after nerve injury. Dark lines, means; shaded regions, ±SEM. (**C**) Hyperalgesic and allodynic behavioral responses in neuropathic (*n* = 13 mice for paw withdrawal, n = 5 mice for escape acceleration) or uninjured (n = 4 mice for both measures) animals after application of light touch (0.07-g filament), noxious pin, or noxious cold (acetone or 5°C H_2_O drop) stimuli, respectively. Data were quantified by reflexive hypersensitivity (left axis) and affective-motivational escape acceleration (right axis). (**D**) Mean Ca2^+^ activity (Z-scored Δ*F/F* per trial) of all neurons from the same animal for that imaging session, before and after nerve injury, in response to noxious pin prick, noxious cold, and light touch stimuli. Neuron identifications were consistent between stimuli within a day, but not across days (n = 157 and 156 neurons, for days −7 and 42, respectively). (**E**) Mean Ca2^+^ response within the nociceptive ensemble for neuropathic (top row; n = 13 mice, 12,026 total neurons imaged) and uninjured (bottom row; *n* = 4 mice, 5370 total neurons imaged) animals in response to noxious pin or light touch stimuli. (**F**) Venn diagrams of percentages of significantly responding neurons to noxious pin, noxious cold, and light touch before and after nerve injury. (**G**) Overlapping neural populations responsive to light touch within the nociceptive ensemble (pin prick and 5°C water or acetone responsive neurons) after nerve injury (*n* = 13 mice) or in uninjured animals (*n* = 4 mice). Numbers indicate means ± SEM. (**H**) Percentages of nociceptive ensemble activated and escape acceleration per imaging session (light-colored points) and across animal groups and conditions (dark, larger points) show significant correlations [Spearman’s r = 0.54 (normal), 0.33 (Neuropathic), and 0.58 (Uninjured) groups]. All tests results in the figure were analyzed via Wilcoxon rank-sum with Benjamini- Hochberg correction unless otherwise noted. Stars, *P* < 0.01.

We next asked if we could prevent the neural transformation of light touch sensory information into an aversive signal and eliminate chronic pain unpleasantness by gaining genetic access to the nociceptive ensemble with innocuous stimuli in neuropathic TRAP mice. At 21 days post–nerve injury, when allodynia had fully developed (fig. S16, B to E), we delivered a light touch TRAP protocol to express hM4-mCherry in the BLA nociceptive ensemble (neuropathic TRAP^hM4^ mice) (Fig. 4, A and B, and fig. S16). At day 42 postinjury, neuropathic TRAP^hM4^ mice displayed significant allodynia and hyperalgesia, for both reflexive and affective-motivational pain responses, relative to uninjured mice (Fig. 4, C to E).While the injection of CNO in neuropathic TRAP^hM4^ mice did not alter reflexive hypersensitivity (Fig. 4D), we observed a profound decrease in neuropathic affective-motivational behaviors, regardless of stimulus intensity or modality (Fig. 4E and fig. S17, A and B). Uninjured TRAP^hM4^ mice given the light touch TRAP protocol expressed levels of ^hM4^-mCherry in the BLA that were similar to those of nonstimulated control mice (Fig. 4B and fig. 2C), presumably because the nociceptive ensemble does not strongly encode innocuous information under normal conditions (Fig. 1I). We observed neither CNO-mediated changes in affectivemotivational pain behaviors in these uninjured mice nor CNO effects on neuropathic reflexive or affective-motivational behaviors in the absence of hM4 expression (Fig. 4, C to E, and fig. S17, A and B). In addition to tactile allodynia, patients with neuropathic pain often report intense pain in response to cold temperatures (cold allodynia). We therefore ran neuropathic TRAP^hM4^ mice through a two-chamber thermal escape-avoidance assay in which the floor of one chamber was cooled (from 30° to 10°C) (Fig. 4F). Uninjured TRAP^hM4^ mice avoided the cold chamber, while mice with nerve injury showed enhanced avoidance, consistent with allodynia (Fig. 4, F and G). Notably, CNO administration to neuropathic TRAP^hM4^ mice generated a near-total indifference between cold and neutral temperature chambers (Fig. 4, F and G). Together, these results indicate that the BLA nociceptive ensemble is also necessary for the pain aversion associated with allodynia and hyperalgesia during chronic pain states.

**Fig. 4:**
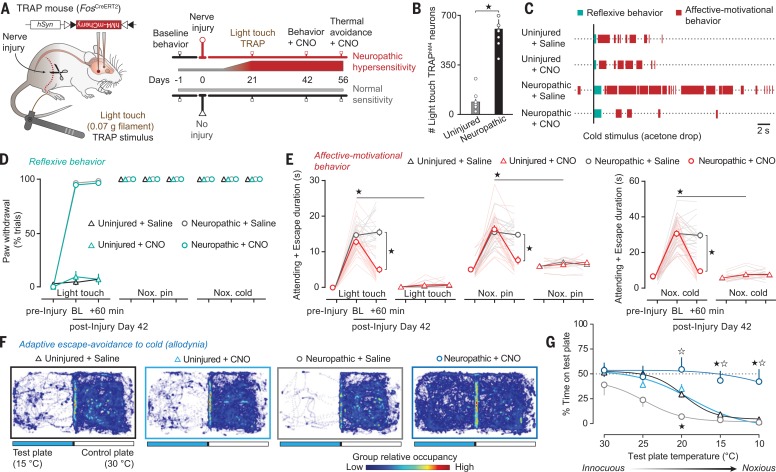
**Inhibition of neuropathic BLA ensemble activity reduces the aversive quality of chronic pain.** (**A**) Utilization of light touch to gain genetic access to, and manipulate, the neuropathic nociceptive ensemble. (**B**) Quantification of light touch TRAP neurons in the BLA of neuropathic mice compared to uninjured mice; *n* = 7 per group. (**C**) Behavioral raster plots from neuropathic mice showing the effects of inhibiting the BLA nociceptive ensemble on reflexive and affective-motivational pain behaviors associated with cold allodynia. (**D** and **E**) Summary of the effects of ensemble inhibition against reflexive (D) and affective-motivational (E) pain behaviors in response to noxious pin prick, noxious cold (acetone drop), or formerly innocuous touch stimuli (0.07-g filament). Behavior was assessed before and 42 days after nerve injury and again at 60 min after CNO or saline administration on day 42; *n* = 14 per group. (**F** and **G**) Effects of neuropathic ensemble inhibition on adaptive avoidance during a cold place aversion assay. (F) Group mean exploration paths, color coded for the relative occupancy time, following CNO or saline treatments; (G) summary of the effects in response to decreasing floor plate temperatures; n = 6 per group. Stars, *P* < 0.05 for all panels. In (G), the black star indicates *P* < 0.05 versus the uninjured + saline group; open star, *P* < 0.05 versus the neuropathic + saline group.Overlaid dots and lines represent individual subjects. Error bars, ±SEM. For (B), Student’s t test; (D and E), two-way ANOVA with Bonferroni correction; (G) three-way ANOVA with Bonferroni correction.

Thus, disrupting neural activity in a nociceptive ensemble in the BLA is sufficient to reduce the affective dimension of pain experiences, without altering their sensory component. The unconditioned nociceptive ensemble described here is a stable network of amygdalar principal neurons that is responsive to a diverse array of noxious stimuli.Within this ensemble, combinatorial neural ensemble codes distinguish the various thermal and mechanical nociceptive stimuli. These codes likely represent stimulus modality, intensity, salience, and valence to provide additional qualitative information that enriches individual pain affect percepts (30). The presence of a purely nociceptive-specific subpopulation of neurons within the larger BLA nociceptive ensemble, distinct from general aversion-encoding populations, suggests the capacity for computing and assigning an accompanying “pain tag” to valence information. This categorical signal could prioritize the negative valence of intense noxious stimuli and scale the selection of conative pain protective behaviors. It is thought that hierarchical pathways transform low-level sensory inputs into higher-order affective responses (5, 31). Our chemogenetic manipulations suggest that this critical node in the nociceptive brain circuitry plays a critical role in shaping pain experiences, by providing an evaluation of nociceptive information that, in turn, intrinsically motivates protective behaviors associated with pain (32).

The phenomenological description of a pain experience is normally that of a complex but unified sensory and emotional perception that can neither exist alone as an unanchored aversive state nor stand merely on its emotionally inert sensory qualities (33, 34). Though activity within the BLA nociceptive ensemble cannot account for the instantiation of the entire pain experience, we propose that the BLA nociceptive ensemble transmits abstracted valence information to the central amygdala, striatal, and cortical networks (35–37). For example, BLA neurons projecting to the CeA may send a “pain tag” that helps select for appropriate defensive responses to impending or immediate threats (23) (supplementary text S2). In parallel, connected cortical regionsmight coalesce BLA affective signals with sensory-discriminative information to process them against prior experiences and internal states for further evaluation at cognitive levels, all of which contribute to the construction of a pain experience (4, 38).

During chronic pain states, BLA ensemble coding of innocuous somatosensory information changes to engage the nociceptive ensemble, leading to perceived aversion and protective behavioral responses when encountering normally nonpainful stimuli, such as light touch. Whether this change in ensemble activity results from peripheral or central sensitization (3, 39), amygdalar input, or intra-amygdala plasticity (11) remains an open question. Chronic pain is not simply a sensory disorder but a neurological disease with affective dysfunction that profoundly impacts the mental state of millions of pain patients (40). Clinical management of chronic pain remains a staggering challenge, given the heterogeneity of underlying causes, and the overreliance on opioid analgesics has contributed to the opioid epidemic (41, 42). Comprehensive strategies that provide substantive relief across pain types are urgently needed (43). To make progress along this translational path, we have identified in the BLA a critical neural ensemble target that mediates chronic pain unpleasantness. This finding may enable the development of chronic pain therapies that could selectively diminish pain unpleasantness, regardless of etiology, without influencing reward, and importantly, preserving reflexes and sensorydiscriminative processes necessary for the detection and localization of noxious stimuli (44, 45). Collectively, our findings begin to refine the neural basis and coding principles underlying the multiple dimensions and complexity of the pain experience for developing more effective analgesic therapies.

## Supplementary Material

An amygdalar neural ensemble that encodes the unpleasantness of pain

## SUPPLEMENTARY MATERIALS

www.sciencemag.org/content/363/6424/276/suppl/DC1

Table S1

References (47–94)
