# Supplementary material for: An amygdalar neural ensemble that encodes the unpleasantness of pain
Source: Science. 2018 Jan 18;363(6424):276–81. doi: 10.1126/science.aap8586 (PMC6450685; doi:10.1126/science.aap8586)
Supplement: An amygdalar neural ensemble that encodes the unpleasantness of pain [file Science-363-276-s1.pdf]

www.sciencemag.org/content/363/6424/276/suppl/DC1

Supplementary Materials for

An amygdalar neural ensemble that encodes the unpleasantness of pain

Gregory Corder, Biafra Ahanonu, Benjamin F. Grewe, Dong Wang, Mark J. Schnitzer*, Grégory Scherrer*

*Corresponding author. Email: mschnitz@stanford.edu (M.J.S.); gs25@stanford.edu (G.S.)

Published 18 January 2019, *Science* **363**, 276 (2019)
DOI: 10.1126/science.aap8586

This PDF file includes:

Supplementary Text
Materials and Methods
Figs. S1 to S17
Table S1
References

Supplementary Text

<u>Supplemental Note 1</u>: This is the first comprehensive demonstration and direct comparative mapping of unconditioned nociceptive and aversive responses in the basolateral amygdala (BLA). For decades, electric foot shock has been the primary punisher for studies on associative fear learning and active avoidance (*47*). It is assumed that electric shocks activate some generalizable unconditioned stimulus (US) network in the BLA (*15*). Prior to the recent development of GECI-based imaging technologies, *in vivo* electrophysiological probes could not evaluate the neural representation of electric shocks due to the passing current. As such, most BLA studies have focused on the conditioned cue response network (CR+) that predict the aversive shocks. Using miniature microendoscope *in vivo* Ca^{2+} imaging approaches, we recently found that electric foot shocks do in fact activate a US network in the BLA that is distinguishable from appetitive and CR+ representations (*19*), which is supported by optogenetic manipulations (*15*). Now, we provide new evidence that electric shock US ensembles have separable overlap with natural, painful stimuli representations (e.g. the nociceptive ensemble, which includes the representations of noxious heat, cold, and pin prick). Not only could we decode the activity patterns distinguishing nociceptive versus shock stimuli with high fidelity (**Fig. S8E**), but we also found that most individual BLA neurons active during electric shocks never displayed nociceptive-related activity (**Fig. 1J and S8B**). Surprisingly, we observed more co-active neurons than expected by chance between electric shocks, and facial air puffs or isopentalamine odorant, than between shocks and the nociceptive stimuli (**Fig. S8D**). This demonstrates that experimental shocks may not be processed in a similar biological mechanism as evolved nociceptive processes (*15*). Depending on the current, voltage, and frequency of the electric shock, as well as the cutaneous bodily location it is applied to (glabrous vs. hairy skin, or finger-tips vs. trunk), there is likely to be activation of a multitude of functionally heterogeneous somatosensory primary afferents, each with their own activation threshold properties, which in turn could transduce competing neural information into the spinal cord "pain gate" (*48*, *49*). This undoubtedly produces an unnatural pain-like percept, or even numerous sensations that are self-reported as perceived itch, light touch, stinging, pressure, and tickle (*50*, *51*). Thus, the identity and function of these non-nociceptive, shock-encoding BLA neurons remains unclear. This is not to say that electric shocks cannot be perceived as aversive, or induce reactive or goal-directed behaviors. Indeed, rodents show increased escape, as well as audible and ultrasonic vocalizations to shocks (*17*, *52*). However, we suggest the use of more natural stimuli, or optogenetic activation of specific peripheral sensory neuron populations (**Fig. S13**) (*53*), over electric shocks when identifying the specific functions of individual circuit elements relating to translational efforts for pain, fear, anxiety, or other psychiatry disorders.

<u>Supplemental Note 2</u>: In addition to the multiple cortical and striatal connections, a major projection target of the BLA is the central amygdala (CeA) complex (*11*), known for executing learned locomotor freezing and flight responses to threats (*54*, *55*). In addition to the BLA nociceptive ensemble described here, there is a possible parallel, or co-requisite circuit for encoding physiologic or emotional pain-related information that ascends from the spinal cord dorsal horn, through the lateral parabrachial nucleus (PBN) to the lateral capsule of the CeA (*56*). Importantly, the PBN→CeA circuit is not selective for nociception, as it is activated by several forms of non-nociceptive information, including itch, nausea, hunger, and bacterial infection (*57–59*). However, inhibition of this pathway reportedly reduces the immediate reactive locomotor response during an electric foot shock (*17*). In contrast, we find that inhibition of the BLA nociceptive ensemble does not eliminate the immediate reactive or reflexive nociceptive response, but does reduce the temporally-delayed, non-stereotyped affective-motivational behaviors that continue beyond active nociceptive input (**Figs. 2 and 4**). Furthermore, the BLA integrates with higher-order corticostriatal circuits (*5*, *18*, *31*, *35*, *36*), whereas the CeA largely influences descending nociception-control circuits, such as the ventrolateral periaqueductal gray (*60–64*). Interestingly, the famous case study of Henry Molaison, also known as Patient H.M., who had a bilateral temporal lobectomy that destroyed the BLA, but left the central amygdala largely intact (*65*), could sense noxious thermal stimuli, but did not verbally report them as "painful" or unpleasant, nor did he protectively guard his arm (*6*). This human self-report and behavioral study offers an important insight toward the shaping of pain unpleasantness that suggests a dissociative role between the BLA and CeA over the conscious qualitative experience of pain negative affect. Further, in primates the BLA is enlarged relative to the CeA, suggesting an increase in importance or influence of this structure in pain or other emotional processing (*5*). Therefore, while we maintain the possibility of a redundant parallel mechanism for affective processing of nociception (*66*), we and others propose that the functional role of PBN→CeA serves as an autonomic alarm system (*58*). In this view, the CeA coordinates with hypothalamic and brainstem endogenous antinociceptive systems for general arousal and motivation, while also integrating abstract affective information from the BLA nociceptive ensemble (*30*). Thus, the CeA might compute danger information, including nociception, as a digital go/no-go reflexive motivational signal, as opposed to the BLA that uses a high-dimensional analog process to abstract not only valence, but also each stimuli's sensory modality and intensity involved in the affective aspects of pain perception (*67*).

Materials and Methods

Animals

All procedures were approved by the Stanford University Administrative Panel on Laboratory Animal Care in accordance with American Veterinary Medical Association guidelines and the International Association for the Study of Pain. We housed mice 1–5 per cage and maintained them on a 12-hour light/dark cycle in a temperature-controlled environment with *ad libitum* access to food and water. Animals undergoing active Ca^{2+} imaging experiments (after mounting the miniature microscope baseplate) were singly housed. For behavioral manipulation and neuroanatomy experiments, we utilized *Fos*-^{CreERT2} mice (B6.129(Cg)-Fostm1.1(cre/ERT2)Luo/J, Jackson Laboratory, stock #21882, male, aged 8–15 weeks at the start of all experiments). For BLA miniature microscope imaging experiments, we utilized C57Bl/6J mice (Jackson Laboratory, stock #664, male, aged 8–12 weeks at the start of experiments). For dorsomedial striatum (DMS) miniature microscope imaging experiments, we utilized wild-type (*Shank3B*$^{+/+}$) or knockout (*Shank3B*$^{-/-}$) *Shank3B*;*Drd1a*$^{Cre/+}$ or *Shank3B*;*A2A*$^{Cre/+}$ mice obtained from Guoping Feng (MIT).

Drugs

4-hydroxytamoxifen (Sigma, #H6278) prepared in Kolliphor EL (Sigma, # 27963), Clozapine-N-oxide (Tocris, # 4936), and 0.9% Sodium chloride (Hospira, #NDC 0409-4888-10).

Viral reagents

Viral reagents for miniature microscope imaging

For Ca^{2+} imaging using GCaMP6m (*68*) in BLA *Camk2a*+ principal neurons, we intracranially injected 500 nL of AAV2/5-*Camk2a*-GCaMP6m-WPRE (Schnitzer lab custom preparation; titre: 6.7 × 10^{12} GC/mL for **Fig. S3A, C** mice) into the right BLA at coordinates anteroposterior (AP): –1.60 mm, mediolateral (ML): +3.32 mm, dorsoventral (DV): –4.70 mm (**Fig. S3B** animals) or AP: –1.70 mm, ML: +3.30 mm (–3.30 mm for left BLA mice), DV: –4.70 mm (**Fig. S3A, C** animals). For Ca^{2+} imaging in DMS D_1 or D_2 dopamine receptor-expressing medium spiny neurons, we injected mice with AAV2/9-*CAG*-FLEX-GCaMP6m (Schnitzer lab custom preparation; titre: 1.37 × 10^{12} GC/mL) at coordinates AP: –0.80 mm, ML: +1.50 mm, DV: –2.5 mm (down to –3.0 then back up to –2.5 mm from dura).

Viral reagents for TRAP studies

For chemogenetic activity manipulation of BLA neuronal ensembles, we intracranially injected 100 nL of either AAV5-*hSyn*-DIO-*hM4-mCherry* (U. North Carolina Viral Core; titre: 3.98 × 10^{12}), AAV5-*hSyn*-DIO-*mCherry* (U. North Carolina Viral Core; titre: 4.72 × 10^{12}), AAVDJ-*Ef1a*-DIO-eYFP (Stanford Viral Core; titre: 2.65 × 10^{11}) into both the left and right BLA at coordinates AP: –1.20 mm, ML: ±3.1 mm, DV: –4.60 mm.

For transdermal optogenetic activation of primary afferent nociceptors, we intrathecally injected 2.5 µL of AAV6-*hSyn*-ChR2(H134R)-eYFP (U. North Carolina Viral Core; titre: 2.17 × 10^{13}) directly into the subarachnoid space so that the virus reaches the CSF and can infect nociceptors.

Stereotaxic injections and surgical procedures

Injection procedures for TRAP study animals

We conducted all surgeries under aseptic conditions using a digital small animal stereotaxic instrument (David Kopf Instruments). We anaesthetized mice with isoflurane (5% induction, 1–2% maintenance) in the stereotaxic frame for the entire surgery and maintained their body temperature using a heating pad. We injected mice with a beveled 33G needle facing medially, attached to a 10-µL microsyringe (Nanofil, WPI) for delivery of viral reagents at a rate of 20 nL/min for more precise targeting (e.g. of DREADD(hM4) expression) using a microinjection unit (Model 5000, Kopf). After reagent injection, the needle was raised 100 µm for an additional 10 min to allow the virus to diffuse at the injection site and then slowly withdrawn over an additional 3 min. After surgery, we maintained the animal's body temperature using a radiant heat lamp until fully recovered from anesthesia.

Injection procedures for miniature microscope animals

We conducted all surgeries under aseptic conditions with glass bead sterilized surgical tools (Dent-Eq, BS500) and used a digital small animal stereotaxic instrument (David Kopf Instruments). We anaesthetized mice with isoflurane (2–5% induction, 1–2% maintenance) in the stereotaxic frame for the entire surgery and maintained body temperature using a heating pad (FHC, DC Temperature Regulation System). For **Fig. S3A, C** mice, we injected using a beveled 33G needle (WPI, NF33FBV-2), facing medially, attached to a 10-µL microsyringe (Nanofil, WPI). We delivered viral reagents at a rate of 250 nL/min using a microsyringe pump (UMP3, WPI) and its controller (Micro4, WPI) for GCaMP expression. We performed injections for **Fig. S3B** mice as described previously (*19*). After reagent injection, we raised the needle 100 µm for an additional 5–10 min to allow the virus to diffuse at the injection site and then slowly withdrew the needle over an additional minute. After surgery, animals recovered from anesthesia on a heating pad to maintain body temperature.

Microendoscope implantation and mounting

Microendoscope implantation in BLA and DMS mice

For BLA-implanted mice run through the protocol in **Fig. S3A, C** and DMS-implanted mice in **Fig. S7A-E**, we performed stereotaxic implantation of a stainless steel cannula 11–19 days after AAV viral injections. We fabricated 1.06-mm-diameter stainless steel cannulas (custom cut 18G McMaster's 89935K66 to 4.2 mm length pieces at Stanford Varian Physics Machine Shop or ordered custom cut 304 S/S Hypodermic Tubing 18G to 4.3 mm length pieces from Ziggy's Tubes and Wires) and attached 2-mm-diameter 0.1-mm-thick Schott Glass (TLC International, custom order) onto one end using optical adhesive (Norland Optical Adhesive No. 81, NC9586074). We ground down the excess glass using a polisher (Ultra Tec ULTRAPOL End & Edge Polisher, #6390) and film (Ultra Tec, M.8228.1), and then placed the completed cannula in a sealed scintillation vial until use during implantation surgeries.

For implantation surgeries, we anaesthetized mice with isoflurane (2–5% induction, 1–2% maintenance, both in oxygen) and maintained their body temperature using a heating pad (FHC, DC Temperature Regulation System). After head hair removal (Nair, Church and Dwight Co. NRSL-22339-05) and opening the mouse skin, we performed small craniotomies in three locations—ML: (–0.7, 2.1, –3.1) mm and AP: (5.2, –3.6, –3.6) mm. We screwed three stainless steel screws (Component Supply Company, MX-000120-01SF) into the skull right up to dura and then performed a craniotomy using a drill (Osada Model EXL-M40) and 1.4 mm round drill burr (FST, 19007-14). We cleaned away bone fragments and other detritus from the opening using sterilized forceps (Fine Science Tools, Dumont #5 Forceps, 11252-20). We continuously applied mammalian Ringers (Fisher Scientific, 50-980-246) to the surgical area when necessary for the remainder of the surgery. To prevent increased intracranial pressure and improve quality of the imaging site, we aspirated all overlying tissue down to ~ DV: -4.20 mm (BLA mice) or -2.10 mm (DMS mice) with a 27G needle (Sai-Infusion, B27-50-27G or VWR Cat. No. 89134-172).

We attached a 1.06-mm stainless steel cannula onto a custom designed 3D printed cannula holder (Stratasys Objet30 printer, VeroBlackPlus material). For BLA-implanted mice, we lowered the cannula to AP: –1.70 mm, ML: +3.30 mm (right BLA) or –3.30 mm (left BLA), DV: –4.50 mm. For DMS-implanted mice, we lowered the cannula to AP: –0.80 mm, ML: +1.50 mm, DV: –2.35 mm. This placed the cannula ~100–300 µm above the imaging plan based on the specifications of the GRIN lens microendoscope's imaging side working distance. Next, we immediately retracted the cannula from the craniotomy site and aspirated any additional debris or blood that had been pushed down during the initial implant then re-lowered the cannula into the implant site, covered the cannula with adhesive cement (C&B, S380 Metabond Quick Adhesive Cement System), and allowed the cement to fix for 2–3 min. We placed custom designed laser cut headbars (LaserAlliance, 18–24G thickness stainless steel) over the left posterior skull screw and applied a layer of dental cement (Coltene Whaledent, Hygenic Perm) to affix both the headbar and cannula to the skull. The cement dried for 7–10 min before we covered the cannula with bio tape (NC9033794 Tegaderm Transparent Dressing), fixed the tape to the cement with ultraviolet (UV) glue (Loctite(R) Light-Activated Adhesive #4305), and allowed the animal to recover from anesthesia on a heated pad.

Verification of microendoscope implantation and GCaMP expression in awake, behaving mice

Several weeks after implantation, we checked awake animals for GCaMP6m fluorescence and Ca^{2+} transient activity on a custom designed apparatus. We avoided using anesthesia as this causes the BLA to exhibit reduced activity or become silent, which might have potentially led us to classify animals incorrectly as unusable due to lack of neural activity even though their neurons might have been active if the animal had been awake. We head-fixed mice by clamping (Siskiyou, CC-1) their headbar and allowed them to run on a running wheel (Fisher Scientific, InnoWheel, Catalog No.14-726-577), which was attached via a custom designed 3D printed part (Stratasys Objet30 printer, VeroBlackPlus material) to a rotary

encoder (Signswise 600P/R Incremental Rotary Encoder). We then lowered a custom-designed 1.0-mm-diameter microendoscope probe based on a gradient refractive index (GRIN) lens (Grintech GmBH) into the stainless steel cannula using forceps or a 27G needle attached to a vacuum line. We attached the miniature microscope onto a holder (Inscopix, Gripper Part, ID: 1050-002199) connected to a goniometer (Thorlabs, GN1) that allowed us to tilt the miniature microscope in x-z and y-z planes. We connected the holder to a three-axis micromanipulator and used it to lower the miniature microscope until we were in the microendoscope's focal plane. To determine an optimal part of the microendoscope to image neural activity, we made minor position adjustments of the miniature microscope in the x-y plane using the micromanipulator. To ensure the entire field-of-view was in focus, we adjusted the miniature microscope's tilt relative to the microendoscope. We used the imaging software (Inscopix, nVista 2.0) to display incoming imaging frames in units of relative fluorescence changes ($\Delta F/F$); this allowed us to observe Ca^{2+} transient activity in the awake behaving mice. We checked for time-locked responses to both auditory (e.g. clap) and sensory (e.g. tail pinch) stimuli, along with any signs of indicator overexpression (i.e. brightly fluorescent neurons lacking Ca^{2+} transient activity). Mice passing both tests moved onto mounting of the miniature microscope baseplate.

Miniature microscope baseplate mounting

In anesthetized (2% isoflurane in oxygen) mice that met the criteria described above, we fixed the microendoscope in place with UV curable epoxy (Loctite(R) Light-Activated Adhesive #4305) and stereotaxically lowered the miniature microscope, with the baseplate attached, toward the top of the microendoscope until the brain tissue was in focus. To ensure that the entire field-of-view was in focus, we used a goniometer (Thorlabs, GN1) to adjust the orientation of the miniature microscope until it was parallel to that of the microendoscope. To fix the baseplate onto the skull, we built a layer of blue-light curable composite (Pentron, Flow-It N11VI) from the dental cement on the mouse's skull toward, but not touching, the baseplate, followed by a layer of UV-curable epoxy (Loctite(R) Light-Activated Adhesive #4305) that affixed the baseplate to the composite. To prevent external light from contaminating the imaging field-of-view, we coated the outer layer of the composite and UV glue with black nail polish (OPI, Black Onyx NL T02). We attached a custom-designed cover (LaserAlliance, 16G thickness stainless steel) to the baseplate to protect the microendoscope. After surgery, mice recovered from anesthesia on a heating pad (FHC, DC Temperature Regulation System). For animals run through the protocol of **Fig. S3B**, we implanted the microendoscope and mounted the baseplate as described in (*19*); we had previously run these mice (n = 8), and *only* these mice of **Fig. S3B**, through a behavioral and imaging protocol as described in (*19*).

Integrated miniature microendoscope imaging and animal test procedure

Miniature microscope behavioral protocol

After mounting the miniature microscope onto a mouse and checking for adequate GCaMP6m expression, we habituated each mouse to the testing environment for at least three days prior to imaging. To preclude any emotional contagion between mice, we brought only one mouse into the isolated, light-, sound- and temperature-controlled testing environment. Further, we housed mice individually.

The experimental procedure for mice (n = 9) analyzed in **Figs. 1** and **3** is described below. A general outline is shown in **Fig. S3A, C**. The experimenter stayed in the testing environment throughout habituation to limit variations related to stress. The main protocol consisted of three or four imaging sessions performed on non-consecutive days (days -7, -5, -3, -1 or -6, -4, -2 pre-SNI) to allow animals to recover, and to reduce photobleaching resulting from long imaging sessions.

At the start of each imaging session, we head-fixed the mouse (using a Siskiyou CC-1), mounted the miniature microscope, checked for GCaMP6m fluorescence, aligned the field of view (FOV) to the previous session FOV, and placed the mouse within the test chamber. Before sensory stimulation, we measured spontaneous neural activity by recording Ca^{2+} activity for 10 min while the mouse habituated to, and freely moved within, the testing box. The mouse received no explicit experimenter-delivered sensory stimuli during this period. After baseline recording, the mouse had 15 min of access to an incentive (sucrose) to capture BLA neural responses to positive valence stimuli. To induce mice to lick without needing prior water deprivation, we used a 10% sucrose solution. We detected licks and delivered sucrose using a custom-built circuit based on a previous design (*69*). A custom electronic circuit (built using Arduino elements) collected lick data and synchronized all incoming data using output TTL pulses from the miniature microscope DAQ. Control signals from this circuit drove a solenoid (NResearch, 161P011) that delivered 10% sucrose instantly after the 1st lick in a bout. We programmed a 5-s-period between liquid deliveries. Thus, even if the mouse licked continuously, this approach provided a sufficient interval between incentive deliveries to relate the evoked neural activity with specific delivery time points.

Next, the mouse began the sensory testing protocol, in which the experimenter delivered a battery of stimuli: 0.07-g and 1.4- or 2.0-g von Frey hairs (light and mild touch); 25G needle (noxious pin prick); water drops at 5ºC or acetone (noxious cold), 30ºC (innocuous liquid, for **Fig. S3A** animals [n = 2]), and 55ºC (noxious heat) delivered via applying a small drop from a 1-mL syringe; fake-out stimuli where no contact was made ("Approach/No contact"); and noise (startle response control). We delivered all stimuli 15 times per session, except "Approach/No contact" and noise, which we delivered 9 times each. See **Fig. S3** for details about timing information related to individual stimuli and stimuli blocks. We wrote custom code in the *R* computing environment to design a randomized stimulus delivery protocol for each session, subject to the following constraints: light touch, noxious cold, mild touch, and innocuous liquid or noxious heat had a set order at the beginning; the same stimuli could not have adjacent stimuli blocks; and "Approach/No contact" stimuli blocks would occur during the first 3 main stimuli super-blocks. We measured withdrawal reflexes and affective-motivational behaviors (attending and escape) using high-speed cameras (AVT Guppy Pro F-125 1/3" CCD Monochrome Camera #68-567 or The Imaging Source DMK 23FM021) and accelerometers (Sparkfun ADXL335 or ADXL345, with data collected using an Arduino Uno or Saleae Logic 8). We included "Approach/No contact" trials to detect possible BLA responses related to expectation of stimulus delivery and error-prediction. These "Approach/No contact" imaging trials consisted of bringing either a 0.07-g von Frey hair, a 25G needle, 1-mL syringe, or a 85-dB noise delivery device toward the animal but neither making contact nor turning on the noise. We randomly interspersed "Approach/No contact" trials between other stimuli blocks. To control for the possibility that the BLA hindpaw stimuli responses were startle-induced, we used a loud tone (~80–85 dB) as an aversive but non-nociceptive sensory stimuli. We delivered the tone (centered around 4 kHz) for 300 ms by triggering an Arduino, loaded with custom code, to drive a TDK PS1240 Piezo Buzzer.

Subsequently, **Fig. S3A, C** mice underwent a modified spared nerve injury (SNI) surgery (see '*Chronic neuropathic pain model'* below for a description of the surgical procedure) (*70*). We then repeated the sensory testing protocol and recording of neural activity at 3, 7, 14, 21, 28, 35, 42 days post-surgery, as thermal and mechanical hypersensitivity developed and persisted (**Fig. S4E-F**). After SNI, mice started showing pain affective-motivational responses such as attending or escape behaviors when experimenters stimulated the injured paw with an innocuous 0.07-g von Frey hair (mechanical allodynia) or 5ºC water droplet (cold allodynia). At times, mice also displayed increased pain affective-motivational responses when stimulated with a sharp pin (mechanical hyperalgesia) or 55ºC water (heat hyperalgesia).

For mice given the procedure in **Fig. S3B***,* a simplified protocol allowed assessment of the interaction between BLA neuron response to innocuous and noxious stimuli. Before stimulation, we measured background neural activity by recording Ca^{2+} activity for 15 min while each mouse freely explored a 17.78 cm × 19.05 cm box. We then transferred each mouse to the behavior testing chamber (10.16 cm × 15.24 cm) where it habituated for ~5 min. We then delivered a battery of stimuli starting with three superblocks, in which 0.07-g (light touch), 0.4-g (moderate touch), and 2.0-g (mild touch) von Frey filament stimuli were each given ten times at intervals of 30 s with 60 s between stimuli blocks and 3 min between superblocks. Next, we applied drops of acetone (noxious cold) 10 times at 60 s intervals followed by pricking the skin with a 25G needle (noxious pin) 10 times. Mice then underwent the SNI surgery and were imaged at days 3, 7, 14, and 21 post-surgery.

Miniature microscope behavior recording hardware

For animals run through the procedure in **Fig. S3A, C**, we synchronized all incoming data in the following manner. The miniature microscope acted as the master controller of event timing as we considered time locking to Ca^{2+} activity the most critical feature. We had two hardware setups for collecting all relevant behavior videos, stimulus delivery times, and accelerometer data: one relied on a set of Arduino microcontrollers and the other one used two Saleae Logic 8 (SL8) along with helper Arduinos. In both setups two cameras recorded mouse behavior and were positioned either below the mouse, to capture stimulus delivery and reflexive responses, or facing the test chamber, to capture the mouse's affective-motivational behaviors. In the first setup (**Fig. S3A** mice**)**, the miniature microscope DAQ output a TTL that drove both cameras by triggering interrupt pins on an Arduino Uno, which then drove each camera, allowing us to synchronize each camera video frame with the Ca^{2+} imaging data. We used the Image Acquisition Toolbox in MATLAB (Mathworks) to collect TTL triggered video frames from each camera. A separate Arduino Mega element collected information on stimulus delivery times via a custom circuit that allowed the experimenter to select the current stimuli, using a keyboard (Adafruit, Product ID #1824) and LCD display (Adafruit, Product ID #772), and to click a button upon stimulus application to record the delivery time for later analysis. A third Arduino Uno measured the analog voltage signal from the accelerometer (Sparkfun ADXL335 or ADXL345) attached to the miniature microscope. The last Arduino measured the onset of licks and sent control signals to open a solenoid (NResearch, 161P011) to release sucrose. Both accelerometer and stimulus Arduino outputs had internal session times based off of each Arduino's internal clock as well as from the miniature microscope frame

number (via the TTL) included in their output; we used the miniature microscope frame numbers to do the final synchronization in later analyses with the miniature microscope Ca^{2+} imaging data. Each data-collecting Arduino received a synchronizing TTL signal from the miniature microscope's DAQ and streamed data to a PC where we saved the information using a custom MATLAB script.

In the second setup (**Fig. S3C** mice), the first SL8 measured analog outputs from the miniature microscope attached accelerometer (100 Hz sample rate, see **Fig. 1A**), connected via a four- (Daburn Electronics & Cable, #2714/4) or a five- (Daburn Electronics & Cable, #2714/5 or Cooner Wire Company NMUF5/36-2550SJ) conductor wire; simultaneously, we recorded in the same SL8 timestamps for the onsets of licks and control signals for sucrose delivery (triggered on the rise of each signal pulse to obtain exact timing information). To collect the latter data, we designed a separate circuit consisting of two Arduinos (Uno and Mega) and a custom lick detector that measured the mouse's licks (signal #1) and sent a control signal (signal #2) to turn on a solenoid. The first SL8 recorded both signals #1 and #2. The second SL8 collected stimulus-onset times from a custom-designed circuit that allowed experimenters to select a stimulus and press a button to timestamp when they delivered a particular stimulus. Saleae software (Logic 1.2.xx) recorded and saved all data from each SL8. We wrote custom Python and MATLAB scripts to extract the data for use in subsequent analysis.

For all mice, we manually checked each session's annotated stimulus-onset time, using a custom MATLAB program to scroll manually through a video recorded from a camera positioned below the mouse. Using this program, we corrected instances in which the annotation did not match the actual onset time of stimulus delivery. To ensure accuracy, we frame-locked this camera to the miniature microscope by triggering each video frame collected by the microscope's DAQ 'sync out' TTL line. We used the stimulus timestamps collected in the imaging sessions of **Fig. S3A, C** mice to automatically jump to the estimated stimulus onset time frame in the behavioral video, which facilitated the manual determinations of stimulus onset-times.

Miniature microscope recording parameters

We recorded all miniature fluorescent microscope videos at a frame rate of 10 or 20 Hz using between 213 ± 3 and 390 ± 7 μW LED light intensity (measured from miniature microscope GRIN with a Thorlabs PM100D and S120C) and saved each frame as a 12 bit image (of varying size, analyzed in a range of 250–275 × 250–270 pixels after down-sampling in each spatial dimension by a factor of 4 from the raw data). We used a stage micrometer (WARD's Natural Science, 94 W 9910) to empirically calculate each pixel to be 2.51 μm × 2.51 μm.

Noxious and aversive stimuli experiments

We delivered a range of noxious, aversive, and appetitive stimuli to animals (**Fig. S8**): noxious cold (acetone), noxious heat (~55°C water), noxious pin (25G needle), air puff (300 ms), isopentylamine (~85 mM in H_2O, delivered via 300-ms air puff), loud noise (~85 dB for 300 ms, same as previously described), electric footshock (0.6 mA for 2 s), quinine (0.06 mM), and 10% sucrose. We habituated mice to a fear conditioning test chamber, similar to our previous setup (*19*), for 30 min on four consecutive days prior to conducting experiments. After mounting of the miniature microscope, we allowed mice to habituate for 10 min to the test chamber, followed by an additional 10 min of *ad libitum* access to 10% sucrose. We then followed the test procedure outlined in **Fig. S8A** and used the same data collection hardware as in **Fig. S3C**. Because the main behavior chamber and the fear conditioning chambers were in separate rooms, we allotted time for the mouse to re-habituate to the fear conditioning chamber for 10 min after the *ad libitum* quinine access. We cleaned all chambers with 70% ethanol before each experimental procedure.

We delivered noxious cold (acetone), noxious heat (~55°C water), noxious pin (25G needle), and loud noise (~85 dB) as described above. Isopentylamine (Sigma-Aldrich SKU #126810, CAS #107-85-7) is an odor shown to be aversive in multiple previous studies (*71–73*). We placed 50 μL of isopentylamine onto a small piece of tissue paper (Kimtech, #05511) and placed this immediately into a 10-mL blood serum tube (Fisher # 02685A) and re-capped. We then inserted two 16G needles through the tube cap and attached these to a valve (Gems Sensors and Controls, MB202-VB30-L203) controlling air delivery to a metal tube used to manually direct odorant to animals in the test chamber. We delivered air puffs through a blunt, 16G needle. We delivered both isopentylamine and air puff for 300 ms with medical-grade compressed air (UN1002) at between 20 to 30 PSI. We aimed isopentylamine and air puff stimuli during delivery at the nose and front half of the face, respectively. Mice received quinine (0.06 mM in deionized water (*74*)) after licking a metal tube in an identical manner as 10% sucrose but through a different tube to avoid contamination. For footshock trials, we habituated mice for 10 min followed by five deliveries of a 0.6-mA electric footshock, with 2 min between each stimulation. To synchronize the onset time of each footshock with Ca^{2+} imaging data and each behavior cameras' videos, we collected TTLs

output by the miniature microscope DAQ and footshock software (Freeze Frame, Actimetrics) on a Saleae Logic 8 DAQ box, which allowed us to determine the specific image frames of the Ca^{2+} video that were synchronous with each footshock. We collected all subsequent data, processed the Ca^{2+} videos, and performed analyses as in the main protocol used in **Fig. S3**.

Clozapine-N-oxide control experiments

To check for possible alterations of neural activity in the presence of CNO alone (i.e. no hM4 expression), we conducted a shortened version of the main protocol (**Fig. S11D-G**). We injected mice with CNO (10 mg/kg), then placed them back in their home cage. After approximately 25 min, we mounted the miniature microscope on the mouse's head and placed them within the test chamber. They habituated for 10 min followed by *ad libitum* access to 10% sucrose solution delivered in the same manner as previously described. Mice then received a battery of stimuli: light touch (0.07-g von Frey filament), noxious pin (25G needle), or a loud noise (~85 dB, same as previously described). We collected all subsequent data, processed Ca^{2+} imaging movies, and performed analysis similar to the main protocol used in **Fig. S3**.

Processing Ca^{2+} imaging videos and identifying neurons

Pre-processing of Ca^{2+} movies

We processed all Ca^{2+} imaging data in the MATLAB software environment using methods similar to previous studies (*19*, *20*). To reduce computational processing times and boost signal-to-noise, we down-sampled imaging movies collected from the miniature microscope in both *x* and *y* lateral spatial dimensions using 4 × 4 bi-linear interpolation. To remove motion artifacts, we registered all frames in an imaging session to a chosen reference frame using *Turboreg* (*75*). Rather than register the entire frame, we selected and registered a sub-region of the field-of-view; this allowed us to choose a region with high-contrast features and without artifacts (*e.g.* dust particles on the optics) that could impede registration.

To improve the performance of motion correction, we first normalized the image frames by subtracting from each frame its mean value. We then spatially bandpass-filtered each frame of the movie (cutoff frequency: ~0.10–0.16 cycle/μm using a Gaussian cut-off filter, which highlighted spatial features at the ~6–10 μm scale). We performed an image complement operation on each frame, by subtracting each pixel value from the maximum pixel value in that frame (*i.e.* dark areas became light, and *vice versa*); this inverted the image and generally made the blood vessels and other dark static features appear more prominently, which benefited image registration. We obtained two-dimensional spatial translation coordinates from *Turboreg* by having the algorithm compare each processed frame to a reference frame (the 100th movie frame). We then used the translation values so obtained for each image frame to register the raw Ca^{2+} movie, but pre-processed in a different manner so as to aid cell extraction, rather than spatial registration.

To facilitate cell extraction, we divided each frame of the raw Ca^{2+} movie by a low-frequency bandpass-filtered version of itself (cutoff frequency: ~0.0014–0.0063 or ~0.0014–0.01 cycle/μm using a Gaussian cut-off filter). This served to diminish neuropil and other background fluctuations. We then registered the resulting image frames using the two-dimensional spatial translation coordinates obtained previously.

Since motion correction can cause the movie edges to take on inconsistent borders due to variable translations, we determined the maximum amount all frames were translated during the motion correction procedure in each dimension (t_{max}) and then added a border of size t_{max} pixels extending from the edge of each frame toward the middle of the frame. We set a maximum border size (t_{max}) of 14 pixels (~35 μm). We converted each movie frame to relative changes in fluorescence using the following formula: $\frac{\Delta F(t)}{F_0} = \frac{F(t)-F_0}{F_0}$, where F_0 was the mean image over the entire movie. Lastly, we temporally-smoothed each movie by down-sampling from the original 20 or 10 Hz to 5 Hz; specifically, for a $x \times y \times t$ movie, we bi-linearly down-sampled in $x \times t$ to reduce computational processing times, which is equivalent to performing a 1D linear interpolation in time of the intensity values at each pixel.

Extraction of neuron shapes, locations, and activity traces

After processing each session's Ca^{2+} imaging videos, we computationally extracted individual neurons and their activity traces using the PCA-ICA algorithm (*76*). We used the following parameters for PCA-ICA: $\mu = 0.1$ and a maximum of 750 iterations. The parameter μ is the relative weight of temporal information in ICA, and $\mu = 0.1$ indicates we performed a spatio-temporal ICA with greater weight given to the spatial than to the temporal skewness. The algorithm output a series of candidate spatial filters ($x \times y \times n$) and temporal traces ($n \times t$)—where *n* is the number of neurons, *t* is the frame, and (*x*, *y*) are spatial dimensions—associated with temporally varying sources, which we then manually verified as neurons.

Manual neuron identification

For all imaging sessions analyzed in this study, we used neurons manually identified by a single human scorer. For each imaging session, we loaded a custom MATLAB GUI that displayed the spatial filter and activity trace of each candidate cell, along with the candidate cell's average Ca^{2+} transient waveform. The human scorer also viewed a maximum projection image of all output spatial filters (**Fig. S2F**), on which the currently selected candidate cell was highlighted.

In addition, we noted that ICA (and other neuron extraction algorithms) often yielded candidate sources with images and activity traces that look highly similar to those of real neurons but that are actually associated with neuropil or other sources of contamination in the movie. Thus, we added another GUI interface to avoid including these false positives. Specifically, we cropped the movie to a 31 pixel × 31 pixel (~78 μm × ~78 μm) region centered on the centroid of each candidate cell; we then created movies containing 10 frames before and after the onset of an individual peak in the candidate Ca^{2+} activity trace to help visualize actual transient-related activity in the movie. Each ICA output had up to 24 of these movies created based on each output's highest signal-to-noise (SNR) peaks. We spatially concatenated all of these movies associated with a specific ICA output to create a montage movie that allowed the human scorer to view movie data associated with peaks in the activity trace for each output at once, which eased decision-making. We used several criteria to classify an ICA output as a neuron: minimal overlap of an output's spatial filter with blood vessels or other contaminating signal sources, resemblance of each output's spatial filter to a 2D Gaussian or an expected neuron shape based on prior knowledge (**Fig. S2E**, "spatial filters"), similarity of the spatial filter to activity within the movie and proximity of output's centroid to movie activity (**Fig. S2E**, "activity in movie"), and similarity of the average transient waveform to a typical Ca^{2+} transient waveform as observed using GCaMP6, such as a fast rise time followed by a slow decay (**Fig. S2E**, "activity traces"). Using these criteria, on average a human scorer manually determined 39 ± 1.4% (n = 138 imaging sessions) of ICA outputs to be neurons (**Fig. S2F**).

For animals in which the internal capsule was present and neurons from the piriform cortex were within the imaging plane, we used a custom MATLAB GUI to manually selected a region corresponding to the location of putative BLA neurons and excluded all other neurons in the imaging plane not within this region (**Fig. S2D**). We used an additional criterion regarding the cellular activity rate, since the piriform cortex often had higher overall rates of activity than BLA neurons that made them distinguishable. All references to 'neurons' within the context of miniature microscope imaging in this study refers to these manually curated BLA neurons from PCA-ICA and subsets therein (**Fig. S2D-F**).

Ca^{2+} transient detection and activity trace binarization

To detect Ca^{2+} events (used in analysis of movement-related, stimulus-induced, and spontaneous neuronal activity), we used a threshold-crossing algorithm similar to previously described methods (*20*). To reduce detection of spurious, high SNR noise, we spatially smoothed the signal by averaging over a 600 ms sliding window. To remove baseline fluctuations, we calculated a sliding median (40 s window) and subtracted this from the activity trace. To capture transient events during the rise time, we took the time-derivative of the resulting trace, calculated the standard deviation (σ) for each signal, and identified any peaks that were ≥2.5 s.d. above baseline noise while enforcing a limit of a minimum inter-event time of >10 frames (2 s). We created a binarized activity vector for each neuron in which all frames associated with candidate peaks were assigned values of one and all other non-event frames assigned values of zero. We concatenated all n neurons binarized activity vectors into an $n \times f$ matrix that we used in subsequent analysis, where indicated.

Calculation of spontaneous firing rate

To assess whether the spontaneous firing rate of BLA neurons changed. For all mice run through the **Fig. S3** protocols and with available baseline miniature microscope imaging session data (either 10 or 15 min), we calculated the mean event rate irrespective of the animal's movement, or other states, during the baseline period. For all mice, we defined Ca^{2+} transients as in "*Ca^{2+} transient detection and activity trace binarization*" and determined the mean Ca^{2+} event rate by calculating the mean rate of Ca^{2+} transients during the baseline period for each neuron. To calculate the mean Ca^{2+} event rate across the neuron population, we took the mean over all neurons' spontaneous rates. To compare spontaneous rates across mice, and before and after the mice were in a neuropathic state, we first calculated the mean population firing rate of all pre-SNI or -sham surgery sessions for each mouse individually. We then used this mean value to normalize the spontaneous Ca^{2+} population event rates measured for the same animal during all subsequent imaging sessions (**Fig S15A-B**). We calculated the nociceptive ensemble firing rate (**Fig S10A-B**) identically, but the final population rate only included neurons within that session that were classified as within the nociceptive ensemble (see *Definition and calculation of nociceptive ensemble*).

Identification of neurons with significant stimulus-evoked responses

Identification of responsive neurons

To determine which neurons significantly responded to a given stimuli, we took neuronal activity data (PCA-ICA output traces) from a 2-s-post-stimulus interval for all trials (creating a $n \times t \times f$ matrix, where n = number of neurons, t = number of trials, and f = number of frames per trial) and binned it into 1-s bins by taking the mean of each bin's $\Delta F/F$ activity. For each cell, we then compared the binned activity response values to those in an identically binned 2-s window from –5 s to –3 s before the stimuli. We pooled this activity across all presentations of a specific stimulus and calculated a p-value for each neuron using a one-tailed Wilcoxon rank-sum. We designated any neurons for which $P < 0.01$ as being significantly responsive to a given stimulus.

Definition and calculation of nociceptive ensemble

We defined the *BLA nociceptive ensemble* in two ways throughout this study. For studies of mice in a normal, non-neuropathic state (**Fig. 1**), we defined the nociceptive ensemble as consisting of neurons responsive to noxious pin (25G needle), noxious heat (55°C water), or noxious cold (5°C water). When mice were in a neuropathic state or had undergone a sham surgery (**Fig. 3**), we defined the nociceptive ensemble as neurons responsive to either noxious pin or noxious cold (5°C water or acetone) (**Fig. S3A, C** animals) or to either noxious pin or acetone (**Fig. S3B** animals). In all cases, we separately assigned stimuli responsive neurons to the nociceptive ensemble for each session using the above definitions. First, we identified significantly responding neurons (see *Calculation of stimuli responsive neurons*) for each stimulus individually, and then we identified neurons responding to any stimuli within the above definitions as part of that session's nociceptive ensemble. For specific cases as noted within the text, we restricted subsequent analyses to neurons within the nociceptive ensemble.

Spatial distributions of significantly responsive neurons

To calculate the spatial distributions of significantly responsive neurons, we first computed each neuron's centroid location. For each neuron's $x \times y$ spatial filter output by PCA-ICA, we binarized the image by calculating the maximum value and set all values below 50% of this value to 'zero' (not part of the neuron) and the remainder to 'one' (part of the neuron). We then set to 'zero' any pixels not connected to the maximum value using a union-finding algorithm implemented in a standard MATLAB function. The x and y coordinates for all parts of each neuron's spatial filter image that are still labeled 'one' were found and multiplied by their true values in the original spatial filter imaged. We then calculated the arithmetic mean of each dimension's weighted coordinate vector and rounded it to the nearest whole pixel value. This allowed us to obtain centroids that are centered closer to the peak intensity of the spatial filter. We converted all neuron centroid pixel values to metric units (2.51 µm/pixel) and computed the full pairwise Euclidean distance matrix for all neuron-neuron pairs in a session. We then binned distances in 1-µm increments and the empirical cumulative distribution calculated for both all neurons and only for neurons significantly responsive to each stimuli (**Fig. S5H**).

Cross-day analysis of BLA neuronal activity

To match neurons across days we implemented a multi-step algorithm similar to previously published work (*19*, *20*). We thresholded spatial filters from PCA-ICA by setting to zero any values below 40% the maximum for each spatial filter and used these thresholded filters to calculate each neuron's centroid, see "*Spatial distribution of significantly responsive neurons and neuron centroid calculation*". We modified that procedure for cross-day alignment by not rounding each neuron's centroid coordinates to the nearest pixel value in order to improve accuracy of cross-day alignment. We created simplified spatial filters that contained a 10-pixel-radius circle centered on each neuron's centroid location; this allowed us to register different days while ignoring any slight day-to-day differences in PCA-ICA's estimate of each neuron's shape even if the centroid locations were similar.

For each animal, if we had N sessions to align, we chose the $N/2$ session (rounded down to the nearest whole number) to align to (*align session*) in order to compensate for any drift that may have occurred during the course of the imaging protocol. For all other imaging sessions, we first created two neuron maps based on the thresholded spatial ("thresholded neuron maps") and 10-pixel-radius circle ("circle neuron maps") filters described above (see **Fig. S6A**) by taking a maximum projection across all x and y pixels and spatial filters (max in 3rd dimension of $x \times y \times n$ neuron filter matrix, where n = neuron number). We registered these neuron maps to the align session using *Turboreg* (*75*) with rotation enabled for all animals and isometric scaling enabled for a subset of animals in cases where that improved results. First, we registered the thresholded neuron maps for a given session to the align session. Second, we used the output 2D spatial

transformation coordinates to also register the circle neuron maps followed by registration of the circle neuron map with that animal's align session. We applied the resulting 2D spatial transformation coordinates to the thresholded neuron map. We repeated this procedure at least five times (**Fig. S6A**). We used the final registration coordinates to transform all spatial filters from that session so they matched the align session's spatial filters and repeated this process for all sessions for each animal individually.

After registering all sessions to the align session, we then re-calculated all the centroid locations as described above. We set the align session centroids as the initial seed for all *global cells.* Global cells are a tag to identify which neurons are matched across imaging sessions; for example, global cell #1 might be associated with neurons #1, #22, #300, #42, and #240 across each of five imaging sessions, respectively. Starting with the first imaging session for an animal, we calculated the pairwise Euclidean distance between all global cells' and the selected session's neurons' centroids. We then determined any cases in which a global cell was within 5 µm (nominally ~2 pixels) of a selected session's neurons. In such cases, the algorithm added that neuron to that global cell's pool of neurons, the global cell's centroid re-calculated as the mean location between all associated session neurons' centroid locations, and any unmatched neurons in that session annotated as new candidate global cells. We repeated this process for all sessions associated with a given animal (see **Fig. S6A-C**).

After assigning all neurons across all animal's imaging sessions to a global cell, we then conducted a manual visual inspection of each animal's cross-day registration. We removed imaging sessions that did not align well with other sessions associated with a particular animal. This lead to us removing n = 42 sessions from this analysis across all **Fig. S3** mice. In addition, to quantify our alignment accuracy, we calculated the pairwise distance between all session neurons' centroid locations that are associated with a common global cell and showed that the majority of alignment was below 5 µm (**Fig. S6D-E**). We further confirmed this by taking all global cells associated with at least two or more neurons and comparing their associated neurons' centroid location with the global cell's centroid location (**Fig. S6F**).

To calculate the number of sessions a global cell responded to specific stimuli, we used the classification of significantly coding neurons in "*Determination of significantly responding stimuli neurons*". We then checked for each global cell the number of sessions it responded to a given stimuli while ignoring any global cells who only had activity on a single session (**Fig. S6G-I**). To calculate maximum duration of stimulus responsivity, and because not all sessions were run exactly on the specified protocol days, we used the actual date the imaging session took place on to calculate both the earliest and latest date that a global cell significantly responded to each stimuli and took the difference to obtain a measure for how long a neuron stably coded for said stimuli (**Fig. S6J**).

Analysis of the overlap in neural ensembles responsive to different stimuli

We sought to determine whether the neuronal ensembles responsive to two different stimuli were consistent with a hypothesis of statistical independent coding channels. To test this hypothesis, we needed to compute the likelihood that statistically independent assignments of cells' coding identities would yield the observed level of overlap in the two coding ensembles. There are two ways to calculate the expected level of overlap under an assumption of independence. Prior methods used bootstrapping to estimate an empirical null distribution and compared the actual overlap to that. Here we introduce an alternative, exact solution.

We calculated the extent to which the observed overlap was unexpected by chance as a specific instance of the classic statistics thought-experiment of drawing without replacement balls from an urn containing black and white balls. In our case, we had a population of N neurons and were seeking the probability, p, of having k successes (number of significant neurons for stimulus #2) in a population with pre-defined K successes (number of significant neurons for stimulus #1) in n drawings (number of significant neurons for stimulus #2). Using the *hygecdf* and *hygestat* functions in MATLAB, we calculated p and the expected number of overlap neurons given the actual number of significantly responsive neurons observed for stimuli #1 and #2 (**Fig S9C**). We validated the results through comparisons to shuffle tests based on the same parameters and using 1,000 rounds of 1,000,000 shuffles to construct bootstrapped distributions (**Fig. S9D**). Because the two methods attained nearly identical results, we used the hypergeometric distribution instead of shuffle tests to reduce computational processing times and to obtain an exact p-value.

To determine whether the overlap in coding ensembles became more expected than chance, either before or after spared nerve injury (**Fig. S15E**), we performed Wilcoxon rank-sum tests in the *R* programming language using a Benjamini-Hochberg multiple comparisons correction (*77*) to identify whether the overlap differed significantly that expected by chance (see **Fig. S9E**).

Analysis of stimulus-evoked behavioral responses.

Analysis of accelerometer traces

We collected quantitative measurement of animal movement in response to stimuli using a custom 3D printed part (Stratasys Objet30 printer, VeroBlackPlus material) to attach a tri-axial accelerometer (Sparkfun, ADXL345 or ADXL335 accelerometer) to the miniature microscope body (**Fig. 1A**). We recorded accelerometer x, y, and z channel data using either an Arduino running custom code (20-Hz sample rate) or a Saleae data acquisition device (Logic 8, 100-Hz sample rate) and synchronized each with Ca^{2+} imaging data using TTL pulses from the miniature microscope DAQ box. We store the output voltage from the accelerometer for later analysis at 10-bit resolution (1024 value range, for both Arduino and Saleae setups). For accelerometer data recorded with the Saleae box, we down-sampled to 20 Hz by slicing the 100-Hz data into 50-ms bins and taking the mean value within each bin. We used a median filter (4 frames wide) to remove noise in each accelerometer channel. As we did not know the orientation of the accelerometer at all times, and to remove the static acceleration due to gravity (*78*), we high-pass filtered (zero-phase 3rd-order Butterworth, 0.5-Hz cut-off frequency) the entire accelerometer x, y, and z channel digital signal. We then computed total acceleration (A_t) using the following equation:

$$A_t = \sqrt{(a_x)^2 + \left(a_y\right)^2 + (a_z)^2}$$

Where a_x, a_y, and a_x indicate the x, y, and z accelerometer output channels. To reduce noise, we zero-phase lowpass filtered the total acceleration in MATLAB (butterworth, 1-Hz cut-off, 3rd order). We down-sampled the resulting total acceleration from 20 Hz to 5 Hz by binning the 20-Hz data into 200-ms sections and calculating the mean acceleration within each section. For cases in which we used Z-scored acceleration, we calculated the mean (μ_{pre}) and standard deviation (σ_{pre}) response for 3–5 s before the stimuli and computed the Z-score for the 5 s before and after the stimuli using the following equation:

$$Z_{score}(t) = \frac{A_t(t) - \mu_{pre}}{\sigma_{pre}}$$

Where *t* is the frame relative to stimulus onset. We then compiled acceleration data for all animals (n = 9, 8–11 sessions each) before and after spared nerve injury (**Fig. S4A, C-E**). To determine whether animals had moved statistically more than chance, before and after spared nerve injury, we calculated the mean session response per animal in a 2-s window after each stimulus and compared it to a baseline from 3–5 s before each stimulus. We determined significance by calculating all p-values < 0.05 using a Wilcoxon rank-sum test with Benjamini–Hochberg multiple comparison correction. When calculated across all eight stimuli (plus background), we found that light touch showed significant increases in behavioral responses (**Fig. S4D, E**).

We compared accelerometer data for each stimulus from **Fig. S3A, C** mice to human scored data (see below and **Fig. S4C**) by calculating the % of that stimuli's trials in a session the animal scored as responding to a stimulus and comparing this to the mean acceleration for that stimuli across all trials in the same session. For the comparison for sessions pre-SNI or -sham surgery (**Fig. 1E**), we found that the two measures were highly correlated (Spearman's $\rho = 0.79$, p-value < 0.001) and that in the case of both no injury and neuropathic mice that the accelerometer offered a greater dynamic range to separate noxious stimuli that otherwise saturate with the binary human behavior scoring measure (see inset in **Fig. S4C**).

Manual scoring of miniature microscope behavior videos

We visually identified nociceptive reflex responses in each mouse's video session recordings obtained in the miniature microendoscope studies. We used a similar identification procedure to that outlined in "*Behavioral quantification of acute and chronic pain behaviors*". We used these behaviors and associated video frame annotations for both behavioral analysis and time-locking of Ca^{2+} data to behavior.

Decoding of stimuli based on BLA neuron activity

To test the specificity of the neural code in response to various stimuli delivered to animals run through the protocol in **Fig. S3A, C**, we implemented naïve Bayes classifiers in the MATLAB programming environment using standard MATLAB

libraries. Naïve Bayes classifiers assume statistical independence between predictors (in this study, BLA neurons), but they also work well when this is not the case (*20*). The goal of a naïve Bayes classifier is to predict the response, *y* (stimuli given at time point *t*) based on predictors, $x_{1\text{-}n}$ (BLA neurons), where *n* is the number of neurons in our case. Formally:

$$P(y(t), |x(t)_1, \dots, x(t)_n) = \frac{P(y(t))P(x(t)_1, \dots, x(t)_n|y(t))}{P(x(t)_1, \dots, x(t)_n)}$$

Where *t* is a particular post-stimulus frame during the session, *P(y(t))* is the probability of a stimulus at a given time point, *P(x|y)* is the probability of activity in neurons (*x*) given a stimulus was present (*y*), and *P(x)* is the probability of a neuron being activated at time *t*. Thus *P(y|x)* gives us the probability of stimulus *y* given we observe *x* activity pattern within the entire BLA neural ensemble. We then use the following classification rule under the assumption of statistical independence between predictors (e.g. BLA neurons or *x*):

$$\hat{y}(t) = \arg\max_y P(y(t)) \prod_i^n P\left(x_i(t)|y(t)\right)$$

This rule allowed us to predict at each time point, *t*, the most likely stimulus (class of $\hat{y}(t)$) given the observed response of all *n* neurons. We first split the neural data into testing and training sets on a per trial basis with 70% of trials for training and 30% for testing. We performed 50-fold cross validation by training a new decoder using randomly picked set of training trials and testing that decoder on a non-overlapping set of test trials from the entire set of stimuli trials. Crucially, since we created decoders that had to simultaneously predict either nine (**Fig. 1L**) or ten (**Fig. S8E**) stimuli, we sought to avoid biases due to some stimuli appearing on more trials than others. To correct for this, we limited the number of each stimuli's trials used for testing and training to whichever stimuli in that session had the minimum number of trials, but we still allowed sampling from the full range of trials for each stimulus during each test round.

For the training set, we constructed a $n \times f$ matrix consisting of all *n* neurons in a session and *f* frames composed of all 10 frames in a 2-s window after stimulus delivery for all trials and stimuli (excluding 30°C water). The response was a $1 \times f$ vector composed of the same 2-s window after the stimulus for all chosen stimuli trials, marked to indicate which stimuli trial each frame was associated with. We used the predictor matrix and response vector to train a naïve Bayes classifier using the *NaiveBayes* MATLAB class with a Gaussian distribution assumed for $P(x(t)_1, \dots, x(t)_n|y(t))$, seen as

$$P(x(t)_i|y(t)) = \frac{1}{\sqrt{2\pi\sigma_y^2}} exp\left(-\frac{\left(x(t)_i - \mu_y\right)^2}{2\sigma_y^2}\right)$$

Where μ_y and σ_y are the mean and standard deviation estimates of neuron *i* response to a particular class in *y*. We ran each trained classifier on another $n \times f$ matrix containing the test set neural activity data and the resulting naïve Bayes classifiers predicted stimuli compared to the actual stimuli given during the test set time periods. We constructed a confusion matrix from the predicted and actual stimuli then normalized each column (corresponding to each actual stimuli) by the number of actual stimuli given to allow comparison of the decoder accuracy for each stimulus compared to others (**Fig. 1L** and **Fig. S9B**). To ensure that the decoding specificity was due to individual neurons' specific activity in response to each stimulus, we ran another 50 rounds where we shuffled the stimulus identities used to train the decoder but kept the testing set stimuli unchanged. This removed much of the predicted stimuli specificity (**Fig. S9B**). We used this same procedure to decode nociceptive from other aversive and incentivized stimuli (see *Noxious and aversive stimuli experiments*) in **Fig. S8E**.

Analysis of locomotor behavior in the open-field assay

Determinations of mouse locations

To determine the spatial trajectories of BLA- and DMS-implanted mice performing the open-field locomotor assay, we used custom *ImageJ* and MATLAB code to analyze video recordings of the mouse behavior (*79*). First, in *ImageJ*, for each video frame we extracted binary masks of the mouse by using manually-determined intensity thresholds. This allowed us to move

the threshold higher or lower to ensure we captured the mouse on the maximal number of movie frames. To remove unwanted pixels from the resulting masks (black on white background) and to make later processing easier, the algorithm then performed a single round of erosion on each mask, by setting any pixels to white that did not have at least eight neighboring black pixels in a 3 × 3 grid. Subsequently, we spatially smoothed the mask to reduce jitter in the trajectory by thrice dilating each image, which involved setting any pixels to black with at least one pixel that was black in a 3 × 3 region. We used *ImageJ*'s particle tracking algorithm (*ParticleAnalyzer.java*) to identify candidate animal locations. We restricted the algorithm to finding objects whose area was between 100 and 20,000 pixels and identified the centroid locations.

Because the particle tracking algorithm output multiple candidates for each imaging frame, we then used custom MATLAB code to identify and retain the largest particle (assumed to be the mouse) identified in each frame. For any frames missing tracking (for example, an animal was obscured from the FOV or left the imaged area) we assigned missing values. To validate the accuracy of mouse tracking, for a subset of behavior videos we added to each frame a marker corresponding to the animal's calculated position on that frame and visually inspected any deviations from the animal's true location in the video. In the movies inspected in this way, we rarely saw the animal's extracted trajectory deviate suddenly or substantially from the animal's actual location, which was consistent with the smooth trajectories seen when visually inspecting locomotor trajectories across the entire behavioral session. Using the spatial trajectories of each mouse (**Fig. S7C)**, we then analyzed the behavior and compared it to BLA and DMS neuron activity (**Fig. S7A-E**).

We calculated the mouse's speed (s) at a given frame, f , of the behavioral video by using the following equation

$$s_f = \sqrt{\left(x_f - x_{f-1}\right)^2 + \left(y_f - y_{f-1}\right)^2}$$

where x_f and y_f are the mouse's x and y coordinates in video frame f. We linearly down-sampled each mouse's spatial trajectory trace s from 20 Hz to 5 Hz to match imaging data and temporally smoothed by a 1-s (5-frame) moving mean. We identified movement bouts by finding all movements of >0.75 cm/s on spatial trajectory traces. We then searched for all transition times when the mouse either initiated or ceased periods of movement. To identify periods of movement initiation, we identified times when the mouse's speed stayed below 0.75 cm/s for 4 s before and above 0.75 cm/s for 4 s after a given time point. The converse was true for movement offsets. We removed any candidates that did not meet these criteria and the final animal movement onset and offset times used in subsequent analysis.

Alignment of locomotor and Ca^{2+} *imaging data*

To analyze the possible modulation of BLA activity by generic movements of the mouse, as opposed to movements that were specifically evoked by noxious stimuli, we examined mouse locomotor speed as a function of the percent of active neurons in mice imaged within the BLA or DMS (see **Fig. S7A**). We found that both D_1 and D_2 dopamine-receptor expressing medium spiny neurons in mice imaged in the DMS were modulated by movement, in line with recent results (*21*). To account for the ~1-s timescale of GCaMP6m synchrony between neurons, as we have observed in the DMS previously (*21*), we first took the binarized neuron activity trace (see *Ca^{2+} transient detection and activity trace binarization*) and computed the mean activity over a 1-s sliding window. We then calculated the percentage of active neurons in the population ("population activity") by taking the mean over the n dimension of the $n \times f$ binarized activity trace (where n = # of neurons and f = number of frames). We then grouped and calculated the mean population activity of all frames in which the animal achieved an instantaneous speed within a defined interval; in this case, we made 0.1-cm/s intervals starting from 0 to the maximum speed of the animal during that session. To compare the degree to which movement modulated neural activity across animals and to control for differences in baseline activity between mice, we normalized by dividing the percentage of active neurons for each mouse's open field session by their mean activity during rest (<0.5 cm/s). Using this procedure, we found that movement speed modulated neural activity in the DMS, but not in the BLA (see **Fig. S7A**).

To characterize neural activity at movement initiation and termination, we used the frames annotated for movement onset and offset (see *Calculation of miniature microscope open field movement bouts*) and the mean percentage of neurons active (without the temporal smoothing done previously to preserve precise onset and offset timing information). We then pulled out from the population-activity trace all frames associated with a 6-s window centered on each movement onset. This produced a $m_o \times f_o$ matrix where m_o is each movement onset bout and f_o is the frames in a 6-s window (3 s before and 3 s after) around the movement onset. We then computed the mean of all these windows at each frame relative to movement onset for each animal's open field session (mean in the 1st dimension of the $m_o \times f_o$ matrix). Lastly, we calculated the mean of all animal mean movement onset responses. We conducted the same procedure for movement offset (**Fig. S7B**).

Statistical analyses

We performed all statistical analyses within the *R* or MATLAB (2015b or 2017a) software environments, unless otherwise noted. Throughout the text, "signed-rank" and "rank-sum" tests refer to Wilcoxon signed-rank and rank-sum tests, respectively. We used the Benjamini-Hochberg (B-H) procedure for all non-ANOVA multiple comparisons correction (*77*)**.** For ANOVA analyses, we performed either a one-way or two-way repeated measures ANOVA via the *aov* function in *R* followed by a Tukey test, when appropriate. When comparing specific hypotheses, we ran the necessary pairwise statistical test followed by a B-H correction. We did not blind the experimenters performing the imaging analyses regarding the cohorts (neuropathic or uninjured) or pain states (pre- or post-SNI) of the mice. However, we used identical code and analysis methods for all cohorts throughout the study. Unless otherwise noted, values and error bars in the text denote means ± SEM.

Code and data availability

For Ca^{2+} imaging video motion correction, the *C* code is available on the author's website (*75*). Our MATLAB implementation of the image registration is also available upon request. Code used for pre-processing Ca^{2+} imaging data, neuron identification and activity trace extraction, ICA output manual cell classification GUI, and animal behavior tracking is available at (*46*). Any other code used in this study's findings, to generate graphs and perform statistical analysis, are available upon reasonable request.

The datasets of this study, approximately 43 TB in size, are available upon reasonable request to the corresponding authors.

Histology for miniature microscope mice tissue

We transcardially perfused all mice used in the imaging protocol in **Fig. S3A, C** with 4% formalin in PBS (Fisher Scientific, NC0238527). We stored brains in 4% formalin in PBS and sectioned them at 100 µm using a vibratome (Leica VT1000S). For staining tissue sections, we washed sections three times in PBS with 0.3% Triton-X100 for 5 min each, blocked with 10% Donkey Serum (Jackson Immunoresearch, 017-000-121 Normal Donkey Serum) in 0.3% Triton-X100 in PBS for 1 hr at room temperature, and stained with primary antibody (Invitrogen α-GFP A11122 rabbit at 1:1000 dilution) overnight at 4°C . The following day (all procedures at room temperature), we washed sections three times for 5 min each in 0.3% Triton-X100 in PBS, stained with secondary antibody (DyLight 549 Donkey α-rabbit at 1:500) for 90 min, stained with DNA stain (AppliChem DAPI BioChemica, 50 nm/mL in 1x PBS) for 20 min, and performed a final wash in 1x PBS. We mounted slices onto glass coverslips with mounting media (SouthernBiotech, Fluoromount-G cat no. 0100-01). We acquired large field-of-view images (**Fig. S2B**) with a standard fluorescence macroscope (Z16, Leica) while we collected zoomed in images (**Fig. S2C**) with a two-photon (Prairie Technologies, Ultima Multiphoton Microscopy System using Olympus LUCPLFLN 20x objective). Where applicable, we only adjusted raw miniature microscope histology images with linear manipulations of contrast and brightness.

Chronic neuropathic pain model

To induce a chronic pain state, we used a modified version of the Spared Nerve Injury (SNI) model of neuropathic pain, as previously described (*70*). This model entails surgical section of two of the sciatic nerve branches (common peroneal and tibial branches) while sparing the third (sural branch). Following SNI, the receptive field of the lateral aspect of the hindpaw skin (innervated by the sural nerve) displays hypersensitivity to tactile and cool stimuli, eliciting pathological reflexive and affective-motivational behaviors (allodynia). To perform this peripheral nerve injury procedure, anesthesia was induced and maintained throughout surgery with isoflurane (4% induction, 1.5% maintenance in oxygen). The left hind leg was shaved and wiped clean with alcohol and betadine. We made a 1-cm incision in the skin of the mid-dorsal thigh, approximately where the sciatic nerve trifurcates. The biceps femoris and semimembranosus muscles were gently separated from one another with blunt scissors, thereby creating a <1-cm opening between the muscle groups to expose the common peroneal, tibial, and sural branches of the sciatic nerve. Next, ~2 mm of both the common peroneal and tibial nerves were transected and removed, without suturing and with care not to distend the sural nerve. The leg muscles are left uncultured and the skin was closed with tissue adhesive (3M Vetbond), followed by a Betadine application. During recovery from surgery, mice were placed under a heat lamp until awake and achieved normal balanced movement. Mice were then returned to their home cage and closely monitored over the following three days for well-being.

Targeted Recombination in Active Populations (TRAP) of BLA neural ensembles

For all TRAP procedures, stereotaxic bilateral injections of viral reagents occurred 3–5 weeks prior to TRAP. Please see ***Fig. S10D*** *and* ***S16A*** *for schematic experimental timelines.*

Acute nociceptive TRAP (noci-TRAP)

We habituated mice to a first testing room (room-A) for three consecutive days. Execution of all TRAP procedures occurred in Room-A. During these habituation days, no nociceptive stimuli were delivered and no baseline thresholds were measured (i.e. mice were naïve to pain experience before the TRAP procedure). In room-A, we placed individual mice within red plastic cylinders (10.16-cm D), with a red lid, on a raised perforated, flat metal platform (60.96-cm H). The male experimenter's lab coat was present in the testing room for the first 30 min of acclimation, and then the experimenter entered the room for the final 30 min of habituation; this was done to mitigate potential alterations to the animal's stress and endogenous antinociception levels. To execute the TRAP procedure, we placed mice in their habituated cylinder for 60 min, and then a 25G sharp pin was applied to the central-lateral plantar pad of the left hindpaw (tibial-sural nerve paw innervation territory), once every 30 s over 10 min. This stimulus frequency was selected to closely match the protocols used in the microendoscope imaging experiments in which significant Ca^{2+} transients were reliably detected in BLA *Camk2a*+ neurons. Following the pin stimulations, the mice remained in the cylinder for an additional 60 min before injection of 4-hydroxytamoxifen (20 mg/kg in ~0.25-mL vehicle; subcutaneous). After the injection, the mice remained in the cylinder for an additional 2 hrs to match the temporal profile for c-FOS expression, at which time the mice were returned to the home cage (*Note*: an immediate return to the home cage following the pin stimulations was considered, but ultimately avoided as potential safety-related neural activity could occur and thus TRAP BLA neurons of putative positive valence in addition to the nociceptive ensemble). To mitigate the influence of contextual memory recall from the noxious TRAP procedure, all subsequent behavioral assays occurred in a second testing room (room-B). In room-B, we placed the noci-TRAP mice within different holding chambers (7.62 × 15.25 × 15.25 cm plastic chamber [white opaque walls]), atop a different metal platform floor (smooth hexagon-hole perforated sheet, McMaster-Carr, #92725T22). Furthermore, the experimenter wore daily disposable lab coats; different from the coat used in the room-A context. After completion of all experiments, we perfused mice and dissected the brains for verification of hM4-mCherry expression in the BLA. We excluded mice with off-target viral expression in the central amygdalar nucleus from the behavioral analysis. Based on this criteria, n = 7 mice study were removed from the final analysis.

Chronic neuropathic pain TRAP (neuropathic-TRAP)

We habituated mice inside individual red plastic cylinders (10.16-cm D) on a raised flat, perforated metal platform (60.96-cm H) for 3 days prior to the start of behavioral sensory testing. After basal thermal and mechanical thresholds were measured, mice underwent a peripheral nerve injury surgery (Spared Nerve Injury, SNI; see "*Chronic neuropathic pain model*" above for details of the surgical procedure). At Day 21 post injury, when mice display significant mechanical and thermal hypersensitivity at the plantar surface of the left hindpaw, we habituated mice as stated above (see "*Acute nociceptive TRAP (noci-TRAP)*"). To execute the light touch-TRAP procedure, a von Frey filament (0.07-g) was lightly applied to the lateral aspect of ventral hindpaw (sural nerve innervation receptive field) with enough force to cause a slight bend of the filament for up to 1 s before being retracted. The filament stimulus was applied once every 30 s over 10 min. We selected this stimulus frequency to closely match the protocols used in microendoscope imaging experiments. Following the filament stimulations, the mice remained in the cylinder for an additional 60 min before injection of 4-hydroxytamoxifen (20 mg/kg in ~0.25-mL vehicle; subcutaneous). After the injection, the mice remained in the cylinder for an additional 2 hrs, at which time the mice were returned to the home cage. At Day 28 post injury, we confirmed neuropathic hypersensitivity persisted. Subsequent behavioral studies to assess chronic neuropathic hypersensitivity and affective-motivational behaviors were conducted beginning at Day 42 post SNI in order to allow sufficient expression of the viral DREADD cargo. After completion of all experiments, we perfused mice and dissected the brains for verification of hM4-mCherry expression in the BLA. We excluded mice with off-target viral expression in the central amygdalar nucleus from the behavioral analysis. Based on this criteria, n = 5 mice were removed from the final analysis.

Optogenetic nociception TRAP (o-TRAP)

Different AAV serotypes display unique infection tropisms. In particular, serotype-6 shows a preferential infection of peripheral primary afferent nociceptor populations (*80*). To express the light-sensitive cation channel channelrhodopsin2 (ChR2) in putative primary afferent nociceptors, we intrathecally injected AAV6-*hSyn*-ChR2(H134R)-eYFP immediately following the i.c. BLA injections of AAV-DIO-DREADD(Gi)-mCherry in TRAP mice while remaining anesthetized under

isoflurane (1–2% maintenance). Specifically, we shaved a small patch of fur on the back, wiped with alcohol and Betadine, and then inserted a 33G beveled needle connected to a WPI Nanofil syringe between the L5/L6 vertebrae and through the dura (confirmation by presence of reflexive tail flick). We slowly administered the virus was over 20 s. We retuned mice to their home cage for 4–6 weeks before behavioral verification of ChR2 expression. In pilot studies, we observed that intrathecal delivery of AAV6 does not uniformly infect all dorsal root ganglion (DRG) neurons across segmental levels. As we sought expression in lumbar DRGs for the purposes of our behavioral experiments that involve sensory testing on the hind limbs, we performed a behavioral screening of each mouse for transdermal light-responsivity when light was applied to the hindpaw. We placed mice inside individual red plastic cylinders (10.16-cm D) on a thin glass surface. A remotely movable fiber optic arm, connected to an 453-nm LED light source (SugarCube) below the glass (~8 mm from the fiber tip to the plantar surface of the paw), was positioned under the heel of the left hindpaw, and a 453-nm ~1-s light pulse was delivered (3 mW/mm^2). We measured whether an immediate nociceptive hindpaw reflex and/or pain affective-motivational behaviors (described below) occurred in response to the light indicated ChR2 expression in nociceptors. If no immediate responses were observed, the fiber optic was moved distally toward the toes and the stimulation was repeated; the location of light-responsivity on the paw was noted for future targeting during the TRAP protocol. We excluded mice from this experiment that exhibited no light-evoked pain behaviors. One week later, we habituated mice on the glass surface for 3 consecutive days (no blue light stimulus was given). Next, on the day of the TRAP procedure, we placed mice inside the cylinders for 30 min. The fiber optic was positioned under the left hindpaw at the previously noted light-responsive site, and we then delivered transdermal light pulses (1 s, 3 mW/mm^2) once every 30 s over 10 min. Following light stimulations, mice remained in the cylinder for an additional 60 min before injection of 4-hydroxytamoxifen (20 mg/kg in ~0.25-mL vehicle; subcutaneous). After the injection, mice remained in the cylinder for an additional 2 hrs, at which time we returned mice to their home cage. Subsequent behavioral experiments were performed 5–8 weeks later.

Behavioral quantification of acute and chronic pain behaviors

For all behavioral tests the experimenter was blind to either the SNI vs. sham procedure, or the injection of CNO vs. saline.

Classification of mouse pain behaviors into reflex and affective-motivational behaviors

In mice, we previously reported our observation that a cutaneous noxious stimulus can elicit several distinct behavioral responses (*81*, *82*): *1. Withdrawal reflexes*: rapid reflexive retraction or digit splaying of the paw that occur in response to noxious stimuli, but cease once the noxious stimulus is removed; and *2. Affective-motivational behaviors*: temporally-delayed (relative to the noxious stimulation), directed licking and biting of the paw (termed "attending"), extended lifting or guarding of the paw, and/or escape responses characterized by hyperlocomotion, rearing or jumping away from the noxious stimulus. *Please see **Fig. S11A** for an illustrative example of these nociceptive reflex and affective-motivational behaviors.* Paw withdrawal reflexes are classically measured in studies of sensitivity, and involve spinal cord and brainstem circuits (as these behaviors are observed in decerebrated rodents (*83*)). In contrast, affective-motivational responses are complex behaviors requiring processing of nociceptive information by brain limbic and cortical circuits. The presence of these complex behaviors indicates the subject's motivation to make the aversive sensation cease, by licking the affected tissue, protecting the tissue, or seeking an escape route (*83–92*).

Pain affective-motivational and nociceptive reflex behavioral assays

To evaluate mechanical reflexive sensitivity, we used a logarithmically increasing set of 8 von Frey filaments (Stoelting), ranging in gram force from 0.07- to 6.0-g (*93*). These filaments were applied perpendicular to the plantar hindpaw with sufficient force to cause a slight bending of the filament. A positive response was characterized as a rapid withdrawal of the paw away from the stimulus within 4 s. Using the Up-Down statistical method, the 50% withdrawal mechanical threshold scores were calculated for each mouse and then averaged across the experimental groups. The response frequency was calculated as the number of positive responses out of 10 stimulations, delivered at 30-s intervals.

To evaluate affective-motivational responses evoked by mechanical stimulation, we used three von Frey filaments (0.07-g, 0.4-g, and 2.0-g) and a sharp 25G syringe needle (pin prick) (*94*). Each filament was applied for 1 s and the pin prick was applied as a sub-second poke to the hindpaw, and the duration of attending behavior was collected for up to 30 s after the stimulation. Only one stimulation per filament was applied on a given testing session.

To evaluate affective-motivational responses evoked by thermal stimulation (*81*), we applied either a single, unilateral 50-µL drop of water (5, 30, or 55°C) or acetone (evaporative cooling) to the left hindpaw, and the duration of attending behavior was collected for up to 60 s after the stimulation. Only one drop stimulation was applied on a given testing session.

To evaluate adaptive thermal avoidance and temperature preference, we placed mice in the center of a linear Thermal Gradient Track (121.92-cm L × 8.25-cm W metal alloy floor; 15.24-cm H black plastic walls), with the floor featuring a temperature gradient along the long axis. Mice freely explored the track for 60 min. To create the temperature gradient, we placed either heating or cooling plates (Bioseb) under the outermost 16.51 cm of the metal floor, with one plate set to 50.0°C and the other set to 0.0°C , creating an actual floor gradient of 48°C → 5°C , respectively. The track was subdivided into 25 temperature zones (4.8-cm D per zone), and we assessed the temperature at the center of each zone by a K-probe thermocouple. "Noxious zone blocks" were designated based on the temperature thresholds for nociceptive behaviors (> 42°C and < 17°C). The track was illuminated by a centered, overhead light (104 lux), and the ambient room temperature was ~26°C . Only one mouse was present in the room during all trials. A video camera placed above the track recorded the position of the mouse within the temperature zones, and videos were later analyzed using a video-tracking software (Ethovision, Noldus) for duration of zone occupancy, zone visits, distance, velocity, and acceleration.

To evaluate active avoidance and escape behaviors to optogenetically driven nociception, mice expressing ChR2 in peripheral primary afferent nociceptors freely explored a custom-built two-choice chamber with LED-lit floor panels. A 32 × 32 LED array (8 × 8 cm) illuminated half the array in blue light, and the other half in red light (~0.3 mW/mm^2). A thin glass surface (0.5 cm thick) covered the array floor, upon which we fitted a black plastic chamber (38 cm H) with a center divider wall containing a square passage hole (5-cm D) raised 2.5 cm from the array floor. The entire LED chamber was maintained in a quiet room with low ambient light (~5 lux). We first placed mice in the red light chamber and then allowed them to freely explore the chambers for 15 min. A camera placed above the chamber recorded the location of the mouse in the apparatus. We manually scored the videos to determine the time spent by the mouse in each chamber (automated tracking was not possible given the light from the LED floor). Only one mouse was present in the room during all trials.

To evaluate neuropathic adaptive cool/cold avoidance behavior, mice with SNI freely explored a two temperature choice chamber. The chamber was constructed from adjoining two thermal plates (Bioseb): one reference plate set at 30°C , and a test plate with the temperature adjusted to either 30, 25, 20, 15, or 10°C for independent trials. The test plate temperature order was randomized for each trial within the day. The chamber (white opaque plastic with no distinguishing features and no divider, 30.48 × 15.24 × 15.24 cm) was fitted onto the conjoined plates. At Day 56 post SNI and at 30-min post CNO injection, we placed mice on the reference plate facing the back wall. Mice then freely explored the chamber for 5 min, while an overhead camera recorded the chamber position and locomotion. After each trial, the mouse was returned to the holding cylinder, while the test plate temperature was rapidly cooled or heated to the next randomly assigned temperature trial. This procedure was repeated until all temperature trials were collected (6 temperature trials total). Video files for each trial were later analyzed using automated tracking software (Ethovision, Noldus) for path tracking, time spent on the test plate, and number of entries onto the test plate.

Anxiety-like assays

The Elevated Plus Maze apparatus was made of blue plastic floors, and consisted of two open arms (30 × 8 cm), two arms enclosed in black plastic walls (30 × 8 × 30 cm) extending from a central platform (8 × 8 × 8 cm) at 90 degrees in the form of a "+". The maze was elevated 30 cm above the floor. We placed individual mice in the center of the apparatus, as an overhead video camera recorded the locomotor paths throughout the 15 min trial. A diffuse overhead fluorescent light (102 lux) illuminated the track. The ambient room temperature was ~26°C . Only one mouse was present in the room during all trials. Videos were later analyzed using a video-tracking software (Ethovision, Noldus) for distance, velocity, time spent in the open arms (body center-point tracking), and entries to the open arm (nose-point tracking).

The open field chamber (circular, 60.96-cm D, 38.1-cm H, opaque white polyethylene walls and floor) was divided into a central zone (center, 25-cm D) and an outer zone (peripheral). We placed individual mice in the peripheral zone, facing toward the chamber wall as an overhead video camera recorder the locomotor paths throughout the 15-min trial. A diffuse overhead fluorescent light (102 lux) illuminated the track, and the ambient room temperature was ~26°C . Only one mouse was present in the room during all trials. Videos were later analyzed using a video-tracking software (Ethovision, Noldus) for total distance traveled, total time spent in the center zone, and mean locomotion velocity as the mouse exited the center zone.

Sucrose-Water Preference assay

To evaluate incentive motivational behavior, we placed mice in a custom made plastic chamber (7.62 × 15.25 × 15.25 cm, 3 white opaque walls, 1 clear plastic wall for video monitoring) with two rounded gavage syringe spouts protruding from small holes in one of the two side walls: one spout dispensed room temperature water, while the other dispensed a room

temperature 10% sucrose solution (in water). Mice then had 20 min to freely sample the spouts. A custom microprocessor controlled release of either solution, which were set to dispense 12 µL of solution upon the first lick, with a minimum 1-s interval between all subsequent lick-induced dispensions. An Arduino using a custom circuit design described previously recorded the number of licks and lick rate while experimenters recorded consumption volume for each spout. To enhance the propensity of mice to actively sample the lick spouts, mice were water deprived 5–8 hr prior to the start of experiments. We repeated the protocol for 7 consecutive days to determine whether any changes in sucrose preference occurred.

Histology for BLA, dorsal root ganglion, and spinal cord tissue

Immunohistochemistry

Anesthetized mice (Fatal-PLUS, Vortech Pharmaceuticals) were transcardially perfused with room temperature 0.1 M phosphate buffered saline (PBS), followed by 10% formalin in 0.1 M PBS. The brain, DRG (L3–L5), and/or spinal cord (lumbar cord L3–L5 segments) were dissected, post-fixed overnight (brains) or for 4 hrs (DRG or cord) at 4°C , and cryoprotected in 30% sucrose in PBS. Tissues were then frozen in O.C.T. (Sakura Finetek, Inc.). Tissue sections (50 µm for brains; 30 µm for spinal cord; and 10 µm for DRG) were prepared using a cryostat (Leica Biosystems) and blocked with PBS containing 5% normal donkey serum and 0.3% Triton X-100 for 1 hr at room temperature. The sections were then incubated overnight with primary antibodies at 4°C . For the chicken anti-GFP antibody, the incubation was performed at 37°C for 2 hrs. After extensive wash with PBS containing 1% normal donkey serum and 0.3% Triton X-100, sections were incubated with appropriate secondary antibody conjugated to AlexaFluor for 2 hrs at room temperature. Sections were then mounted in the glass slide with Fluoromount (Southern Biotech) after washing with PBS for 3 times for 5 min. Images were collected under a Leica TCS SP5II confocal microscope with LAS AF Lite software (Leica Microsystems).

The following primary antibodies were used: Anti-c-Fos (Rabbit, Abcam # ab7963-1), Anti-c-Fos (Rabbit, Synaptic Systems # 226003), Anti-CGRP (Sheep, Abcam # ab22560), Anti-GFP (Chicken, Aves Labs # GFP-1020), Anti-RFP (Rabbit, Abcam # ab62341), Anti-NeuN (Mouse, Millipore # MAB377), Anti-Ret (Goat, R&D Systems # AF482), Anti-NF200 (Chicken, Aves Labs # NFH0211).

In Situ Hybridization

Anesthetized mice (C57Bl/6J, male, 5 - 8 weeks, Fatal-PLUS (Vortech Pharmaceuticals)) were transcardially perfused with 0.1 M PBS followed by 10% formalin in 0.1 M PB. Brains were dissected, cryoprotected in 30 % sucrose overnight, and then frozen in OCT. Frozen tissue was cut into 14 µm thick slices, placed onto Superfrost Plus slides, and kept at −80°C . Tissue was thawed from −80°C , washed with PBS at room temperature, and subsequently processed according to the Advanced Cell Diagnostics RNAscope Technology protocol (ACD Bioscience). We first washed the tissue with solutions from the pretreatment kit to permeabilize the tissue, incubated with protease for 30 min followed by the hybridization probe(s) for 2 hr at 40°C . Images were collected under a Leica TCS SP5II confocal microscope with LAS AF Lite software (Leica Microsystems).

The following RNAscope probes were used: Mm-*Camk2a*-C1 (# 445231), Mm-*Slc32a1*-C1 (#319191), Mm-*Sst*-C1 (#404631), Mm-*Pvalb*-C2 (#421931), Mm-*Vip*-C2 (#502231), Mm-*Rspo2*-C2 (# 402001), and Mm-*Ppp1rb*-C3 (# 405901).

Supplemental Figures

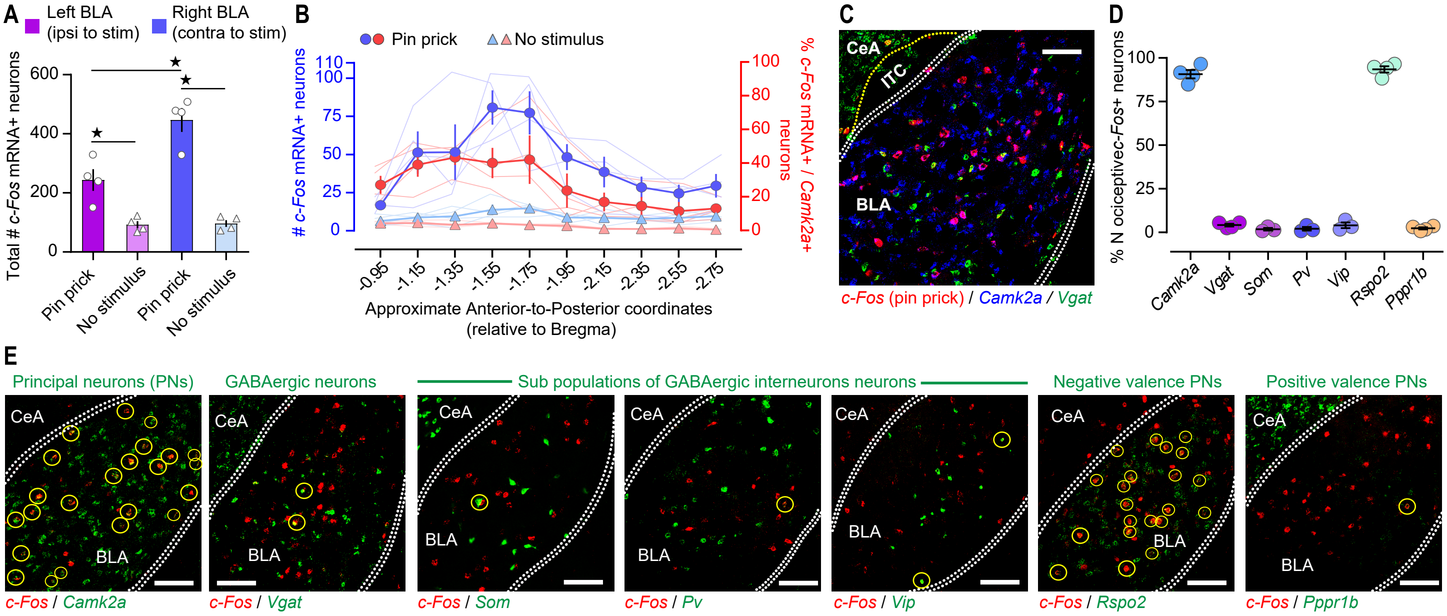

Fig. S1. Associate data for Fig. 1. Molecular and anatomical characterization of neurons constituting the BLA nociceptive neural ensemble. (**A**) Unilateral, left hindpaw pin prick-induced *c-Fos* mRNA expression in the ipsilateral left and contralateral right BLA. (Two-way ANOVA, Bonferroni post-hoc). Stars, at least $P < 0.05$. (**B**) Anterior to posterior quantification of nociceptive *c-Fos*+ neurons in the right BLA (left blue y-axis), and the percentage of nociceptive *c-Fos*+ neurons that are *Camk2a*+ principal neurons (right red y-axis). (**C**) Representative triple fluorescence *in situ* hybridization (FISH) for identifying nociceptive (*c-Fos*), principal (*Camk2a*), and GABAergic (*Vgat*) neurons in the BLA. (**D** and **E**) Quantification of nociceptive subpopulations of BLA neurons (D) and representative double FISH images (E). Yellow circles = co-expressing neurons. Scale bars, 50 µm.

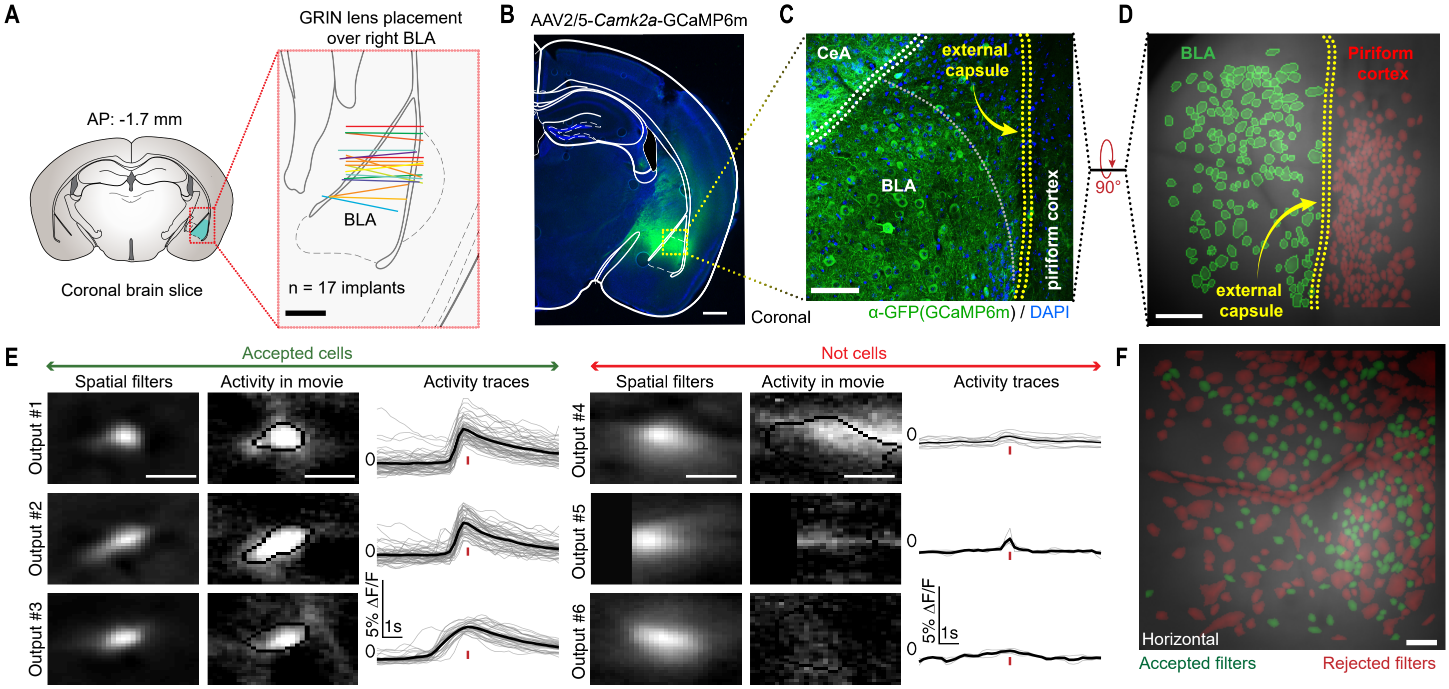

Fig. S2. Associate data for Fig. 1 and 3. Experimental design for microendoscopy imaging of the BLA during noxious and innocuous events. (**A**) Coronal schematic of microendoscope implant locations, in mice, used in miniature microscope BLA pain imaging experiments (n = 17) in **Fig. 1** and **3**. The most dorsal GRIN lens (red) was the only left BLA implant. Scale bar, 300 µm. (**B**) Coronal section of a mouse expressing AAV2/5-*Camk2a*-GCaMP6m ~5 weeks (37 days) post-injection showing healthy, cytoplasmic expression of GCaMP6m (green) within the right BLA along with DAPI DNA staining (blue). Scale bar, 500 µm. (**C**) Zoomed in coronal section of (B) showing anti-GFP staining (Invitrogen α-GFP rabbit A11122) for GCaMP6m (green) and DAPI DNA staining (blue). Scale bar, 100 µm. (**D**) Neuron map of active neurons from an uninjured right BLA imaging mouse from **Fig. 3**. A subset of mice contained miniature microscope fields of view divided into BLA and piriform cortex portions separated by a fibre tract (external capsule) that was darker (due to a lack of GCaMP6m expression). We manually selected piriform neurons based on location and differential activity compared to BLA neurons then excluded them from further analysis. Scale bar, 100 µm. (**E**) Example PCA-ICA neuron extraction outputs from a single mouse (same as in **Fig. 1C-D**) showing accepted and rejected ICA outputs. We manually classified all neurons used in imaging-related aspects of this study based on a variety of parameters, such as the PCA-ICA filter shape, the event triggered movie activity (e.g. whether it confirmed to prior expectation of one-photon neuron morphology and GCaMP activity), location within the imaging field of view (e.g. not within a blood vessel), and the shape of the transient having characteristic GCaMP dynamics. No automated heuristics were used to further remove accepted neurons. "Spatial filters" are the PCA-ICA output filters, "Activity in movie" is a 31 × 31 pixel square region cropped from the movie around the candidate neuron's centroid location during that candidate neuron's transients (black outlines are "Spatial filter" derived neuron contours), and "Activity traces" shows the mean (black) and per transient (gray) PCA-ICA activity of a candidate neuron from the imaging session. Scale bars, 25 µm. (**F**) Example neuron map (same animal and imaging session as **Fig. 1C-D**) showing accepted (green) and rejected (red) filters using criterion in (E). Scale bar, 100 µm.

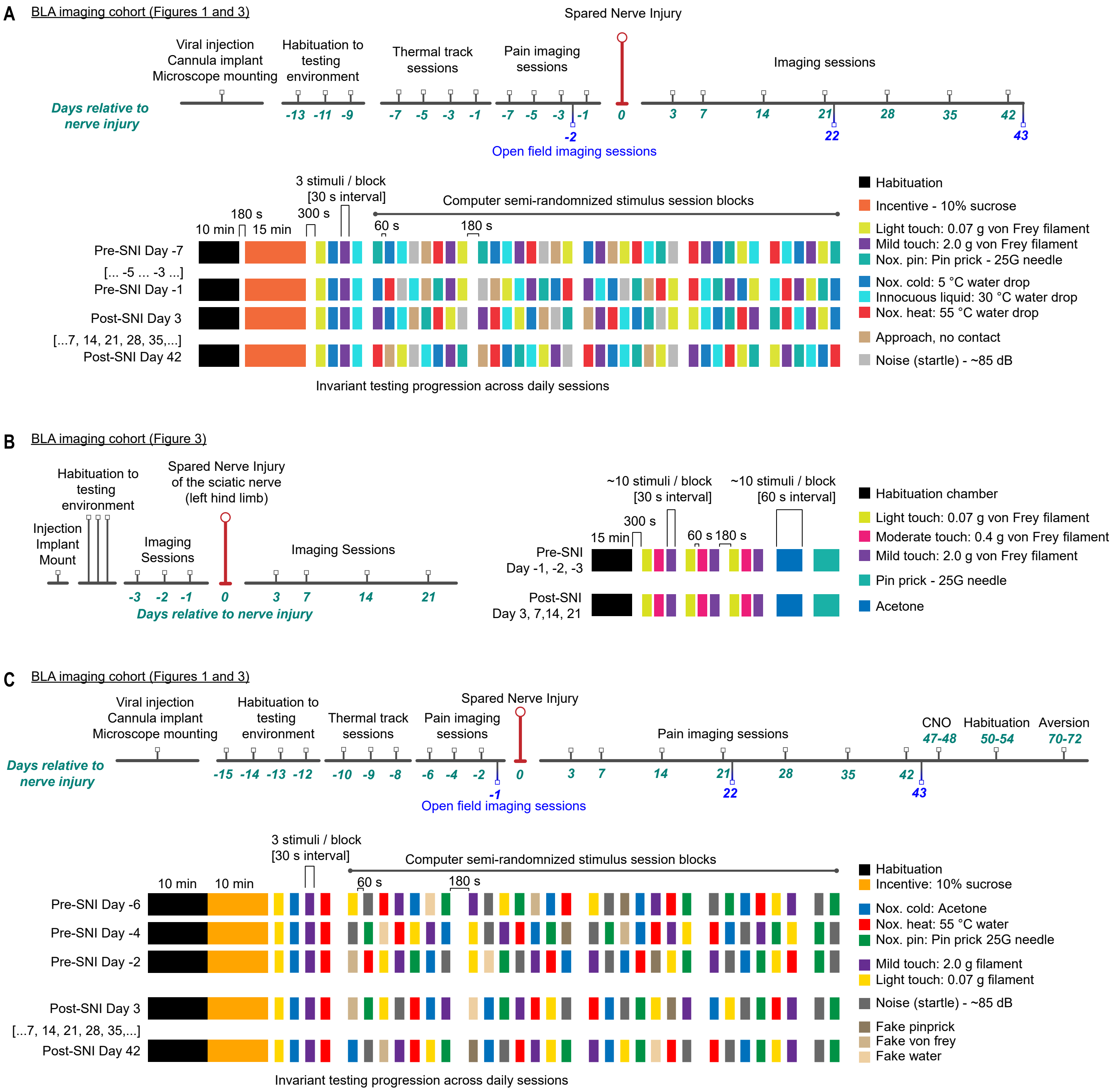
A
BLA imaging cohort (Figures 1 and 3)
Spared Nerve Injury
Viral injection
Cannula implant
Microscope mounting
Habituation to testing environment
Thermal track sessions
Pain imaging sessions
Imaging sessions
Days relative to nerve injury
-13 -11 -9
-7 -5 -3 -1
-7 -5 -3 -1
-2
0
3 7 14 21 28 35 42
22
43
Open field imaging sessions
3 stimuli / block [30 s interval]
Computer semi-randomnized stimulus session blocks
180 s
300 s
10 min
15 min
60 s
180 s
Pre-SNI Day -7
[... -5 ... -3 ...]
Pre-SNI Day -1
Post-SNI Day 3
[...7, 14, 21, 28, 35,...]
Post-SNI Day 42
Invariant testing progression across daily sessions
Habituation
Incentive - 10% sucrose
Light touch: 0.07 g von Frey filament
Mild touch: 2.0 g von Frey filament
Nox. pin: Pin prick - 25G needle
Nox. cold: 5 °C water drop
Innocuous liquid: 30 °C water drop
Nox. heat: 55 °C water drop
Approach, no contact
Noise (startle) - ~85 dB
B
BLA imaging cohort (Figure 3)
Habituation to testing environment
Spared Nerve Injury of the sciatic nerve (left hind limb)
Injection
Implant
Mount
Imaging Sessions
Imaging Sessions
-3 -2 -1 0 3 7 14 21
Days relative to nerve injury
~10 stimuli / block [30 s interval]
~10 stimuli / block [60 s interval]
300 s
15 min
60 s
180 s
Pre-SNI Day -1, -2, -3
Post-SNI Day 3, 7,14, 21
Habituation chamber
Light touch: 0.07 g von Frey filament
Moderate touch: 0.4 g von Frey filament
Mild touch: 2.0 g von Frey filament
Pin prick - 25G needle
Acetone
C
BLA imaging cohort (Figures 1 and 3)
Viral injection
Cannula implant
Microscope mounting
Habituation to testing environment
Thermal track sessions
Pain imaging sessions
Spared Nerve Injury
Pain imaging sessions
CNO 47-48
Habituation 50-54
Aversion 70-72
Days relative to nerve injury
-15 -14 -13 -12
-10 -9 -8
-6 -4 -2
-1
0
3 7 14 21 28 35 42
22
43
Open field imaging sessions
3 stimuli / block [30 s interval]
Computer semi-randomnized stimulus session blocks
10 min
10 min
60 s
180 s
Pre-SNI Day -6
Pre-SNI Day -4
Pre-SNI Day -2
Post-SNI Day 3
[...7, 14, 21, 28, 35,...]
Post-SNI Day 42
Invariant testing progression across daily sessions
Habituation
Incentive: 10% sucrose
Nox. cold: Acetone
Nox. heat: 55 °C water
Nox. pin: Pin prick 25G needle
Mild touch: 2.0 g filament
Light touch: 0.07 g filament
Noise (startle) - ~85 dB
Fake pinprick
Fake von frey
Fake water

Fig. S3. Associate data for Fig. 1 and 3. Experimental protocol for stimulus delivery during BLA microendoscopy imaging. (**A**) Experimental timeline and daily imaging protocol for miniature microscope imaged BLA-implanted mice before and after Spared Nerve Injury surgery (SNI). Each session begins with imaging basal neural activity in the BLA without explicit sensory stimulation (Habituation). Next, mice have free access to a lick port delivering *ad libitum* 10% sucrose (Incentive), which was removed after 15 min. We do not water deprived imaged mice during this or any parts of the protocol. Next, we applied somatosensory stimuli to the hindpaw. The first four stimulus blocks always occur in the same order across days in order to track daily pain behaviors and the development of chronic neuropathic pain. All subsequent stimulus blocks are semi-randomized computer generated sequences within and across days with the conditions that the same stimulus block does not occur twice in sequence, nor does the same daily protocol repeat on any given day. We designed this protocol to be less than 2.5 hr for each animal's imaging session; to give enough stimuli to have sufficient statistical power to identify stimulus-responsive neurons; and to incorporate sufficient "down time" between stimuli, in order to avoid potential photobleaching of imaging area or animal exhaustion. During "Approach" trials either a von Frey filament, water droplet, pin, or noise device was moved toward the animal similar to other trials but with no actual contact or stimulus delivery. Open field imaging sessions consist of 30 min sessions of animals exploring a 2 m diameter circle or 2 m square open field apparatus, see Fig. S7 for additional details and results. (**B**) Similar experimental timeline and daily imaging session layout as in (A). The protocol was simplified to directly assess relationship between innocuous and nociceptive ensembles before and after nerve injury.(**C**) Similar experimental timeline and daily imaging session layout as in (A). In addition, these imaging mice had two additional experimental days. "CNO" was a control imaging session in which CNO was injected and 30 min later mice imaged in response to sensory stimuli, see **Fig. S11D-G** for additional details and results. "Aversion" tested responses of BLA neurons to noxious and aversive stimuli, see **Fig. S8** for additional details and results. "Habituation" involved mice being habituated to the fear conditioning chambers used in "Aversion" imaging sessions.

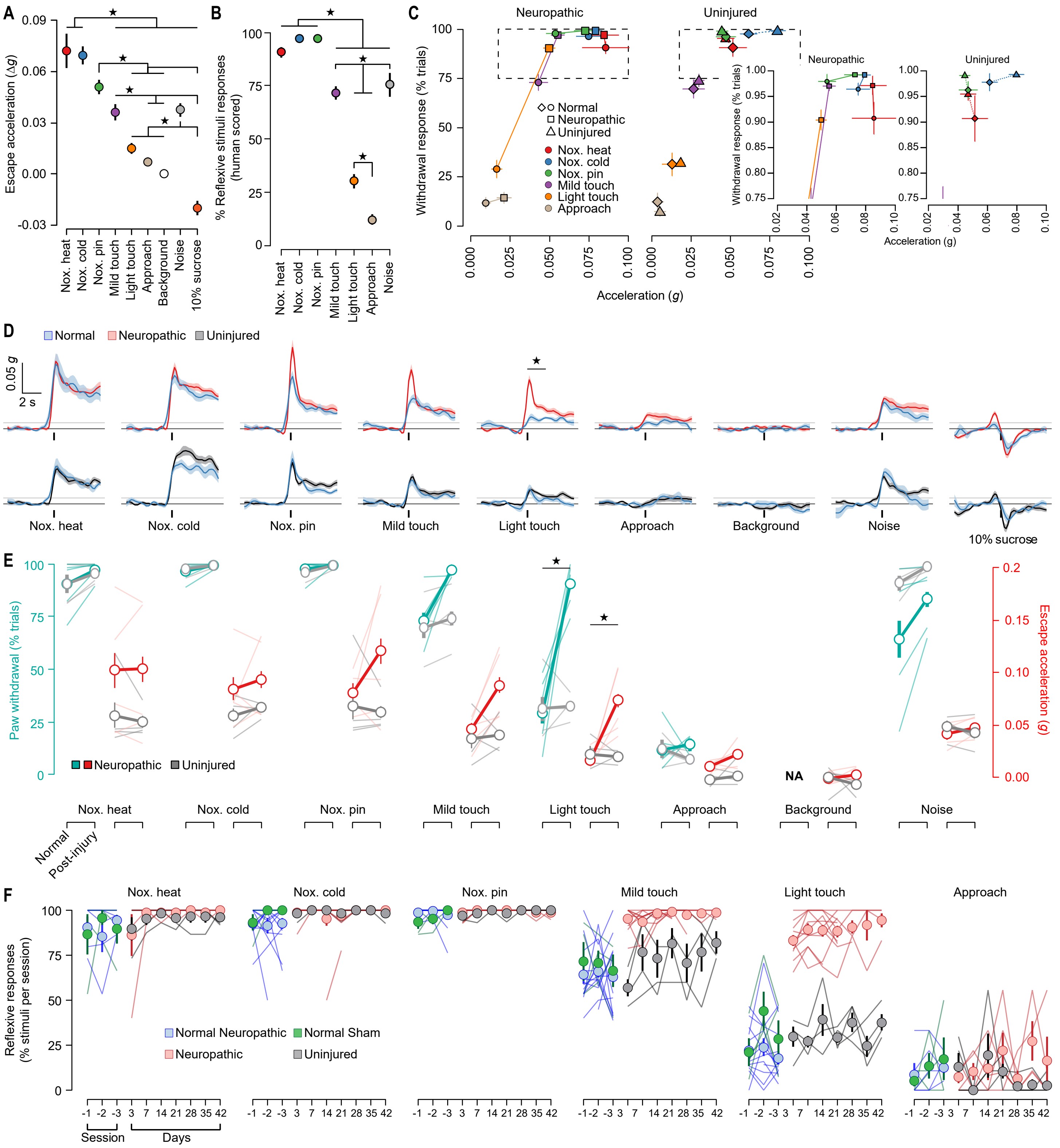

A
B
C
D
E
F
Escape acceleration (Δg)
% Reflexive stimuli responses (human scored)
Withdrawal response (% trials)
Acceleration (g)
Neuropathic
Uninjured
Normal
Nox. heat
Nox. cold
Nox. pin
Mild touch
Light touch
Approach
Background
Noise
10% sucrose
0.05 g
2 s
Paw withdrawal (% trials)
Escape acceleration (g)
NA
Post-injury
Reflexive responses (% stimuli per session)
Normal Neuropathic
Normal Sham
Session
Days

Fig. S4. Associate data for Fig. 1 and 3. Normal and neuropathic pain behaviors to innocuous and noxious stimuli using qualitative and quantitative measures. (**A**) A miniature microscope mounted accelerometer (Sparkfun, ADXL335 or ADXL 345) measured quantitative affective escape or reactive measures of animals' responses to various innocuous (0.07-g and 1.4- or 2.0-g von Frey filaments), noxious (55°C water, 5°C water, acetone, and 25G pin prick), and control ("Approach/No contact" and noise) stimuli. Mean session acceleration for 2 s after stimulus onset before nerve injury is plotted (n = 9 mice). Noxious stimuli all showed significantly more movement than light touch stimuli (One-way ANOVA, Tukey post-hoc). Stars, at least $P < 0.05$. (**B**) Behavior videos for the same animals as in (A) were manually scored to identify whether animals exhibited reflexive, whole-body, or head-jerk responses after stimulus application (note: this was not a measure of nocifensive paw withdrawal responses). Similar to (A), noxious stimuli (55°C water, 5°C water, acetone, and pin prick) all showed significantly (One-way ANOVA, Tukey post-hoc) greater responses than innocuous stimuli. Stars, at least $P < 0.05$. (**C**) To validate that both the quantitative and manual behavior measures produced similar results, data from (A) and (B) were combined on a per session basis for each stimuli. Before nerve injury (circles and diamonds), both metrics are positively correlated (Spearman's ρ = 0.72 [normal] and 0.59 [neuropathic and uninjured], p-value < 0.001). After nerve injury (squares), light touch stimuli (0.07-g, orange) show increased behavioral responses in both metrics that was not seen in uninjured animals (triangles). Inset, zoomed in section (from dotted regions) to better illustrate differences in noxious stimuli responses. (**D**) Using the same metric as in (A), the responses to noxious stimuli showed similar onset dynamics while light touch stimuli induced minimal behavioral response. Light touch showed a marked increase in onset, in peak reflexive behavior, and in continued escape dynamics after nerve injury (*top row*) that was not seen in uninjured mice (*bottom row*), suggesting the presence of neuropathic hypersensitivity and affective allodynia. Further, noxious pin and mild touch stimuli showed heightened responses immediately post-stimulus delivery, suggesting strong neuropathic hypersensitivity has developed, while the lack of an enhanced affective escape response might indicate a saturation or ceiling effect for this measure. The motion responses to "Approach/No contact", Noise, 10% sucrose, and background are anticipatory escape behavior, startle response followed by escape behavior, head motion toward lick port, and mean movement during random times in the trial when explicit stimuli are not being given, respectively. Baseline (black horizontal line), threshold for movement (gray horizontal line, see **Fig. 1M** and **S7G**, I analysis), and stimulus onset time (black vertical tick) are indicated. Stars, at least $P < 0.05$. (**E**) Using data in (D), the mean accelerometer response (2 s window after stimuli, red right axis) and human scored reflexive withdrawal (left axis, cyan, same measure as (B)) in uninjured (grey) and neuropathic (red) animals for all stimuli was calculated. Innocuous stimuli showed a significant increase in activity (p < 0.01, Wilcoxon rank-sum with Benjamini-Hochberg multiple comparisons correction) with both measures while noxious pin and mild touch showing hypersensitivity trends in escape acceleration. Stars, at least $P < 0.05$. NA indicates background manually scored behavioral responses were not measured. (**F**) Human scored reflexive responses for all animals (n = 17) in **Fig. 3C**, see (B) and *Supplemental Methods* for additional details, showing the responses across days before (blue) and after (red) spared nerve injury. Uninjured mice do not show changes in mild or light touch before (green) and after (grey) undergoing a mock surgery (only anesthesia for equivalent time as injured mice). Light colored lines indicate individual mice's responses to each stimuli across imaging sessions. All figure values means ± SEM unless otherwise noted.

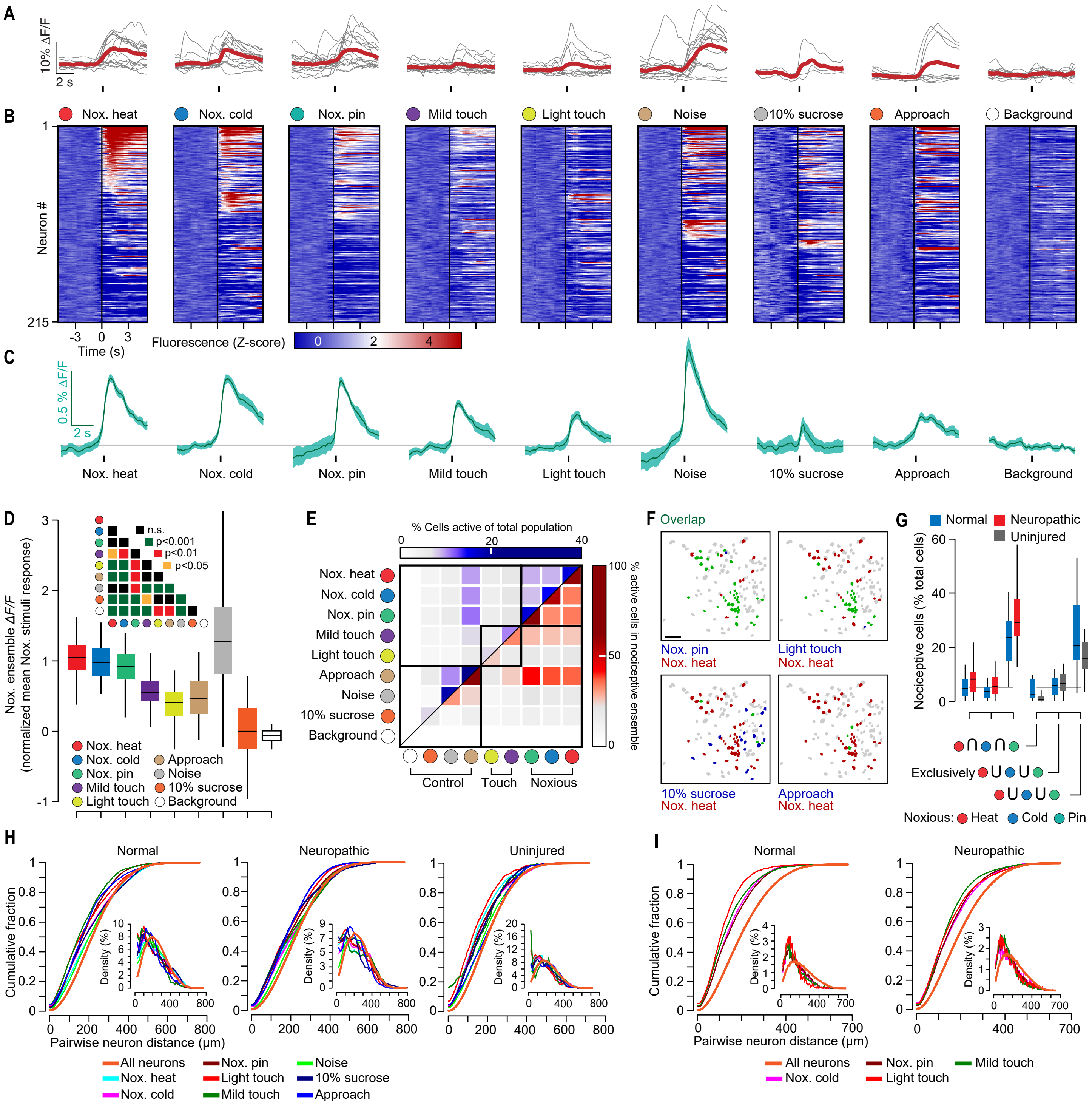

A
10% ΔF/F
2 s
B
Nox. heat
Nox. cold
Nox. pin
Mild touch
Light touch
Noise
10% sucrose
Approach
Background
Neuron #
Time (s)
Fluorescence (Z-score)
C
0.5 % ΔF/F
D
Nox. ensemble ΔF/F (normalized mean Nox. stimuli response)
n.s.
p<0.001
p<0.01
p<0.05
E
% Cells active of total population
% active cells in nociceptive ensemble
Control
Touch
Noxious
F
Overlap
G
Normal
Neuropathic
Uninjured
Nociceptive cells (% total cells)
Exclusively
Noxious: Heat Cold Pin
H
Normal
Neuropathic
Uninjured
Cumulative fraction
Density (%)
Pairwise neuron distance (μm)
All neurons
Nox. heat
Nox. cold
Nox. pin
Light touch
Mild touch
Noise
10% sucrose
Approach
I

Fig. S5. Associate data for Fig. 1. BLA neuron ensembles that are selective and co-active to noxious and aversive stimuli. (**A**) Responses of individual BLA neurons to various noxious (noxious pin, cold, and heat), innocuous (mild and light touch), control ("Approach/No contact", noise, and background), and positive valence (10% sucrose) stimuli in an uninjured mouse. Mean response (red), activity during individual stimuli trials (gray), and stimuli onset time (black tick) are indicated. (**B**) Mean stimulus response (Z-scored $\Delta F/F$) across all trials for all right BLA neurons during a single imaging session in an uninjured mouse (n = 215 neurons). Neuron identifications (rows) across different stimuli are consistent, demonstrating that some neurons encode multiple different types of noxious and innocuous stimuli, while a separate neuron population uniquely encodes nociception. The first three stimuli (noxious heat, cold, and pin) are considered noxious, the mild and light touch are innocuous, the Approach/No contact is a control for animal anticipation of stimuli, the 10% sucrose is an incentive (positive valence), the noise is a mildly aversive control stimuli, and background is a control showing average response during random trial time points at least 10 seconds away from any defined stimuli. (**C**) Temporal dynamics of the mean $\Delta F/F$ of neurons within the nociceptive ensemble for all imaging sessions and mice (n = 9 mice, 3–4 sessions each). (**D**) Mean $\Delta F/F$ of neurons within the nociceptive ensemble for all imaging sessions and mice (n = 9 mice, 3–4 sessions each). Values are an average of two seconds post stimulus as seen in (C). Mean BLA nociceptive ensemble response showed a graded reduction from noxious (55°C water, 5°C water or acetone, and pinprick) to innocuous and positive valence stimuli. Inset, BLA stimulus response was significantly modulated by stimulus type (One-way ANOVA, $F(8,247) = 29.4$, $p < 0.001$) as shown by the table of significant values (Tukey post-hoc) colored coded by p-value thresholds reached (colored) or not significant (black). (**E**) Activation of individual neuron ensembles to specific stimuli in the entire (top half, blue) and nociceptive ensembles (bottom half, red) along the diagonal. The percent of total and nociceptive neuron ensembles co-activated by pairs of stimuli (off-diagonal) showed greater co-activation of hindpaw delivered stimuli compared to noise or 10% sucrose stimuli. (**F**) Spatial locations of neurons, from a single mouse's imaging session, significantly responsive to various noxious (noxious pin and heat), innocuous (light touch), positive valence (10% sucrose), and approach stimuli. A high degree of overlap was seen between noxious stimuli that was absent when compared to positive valence stimuli. Gray neurons are those unresponsive to specific stimuli indicated in each subpanel and green neurons indicate those overlapped between the two stimuli indicated below the subpanels. Scale bar, 100 µm. (**G**) The number of stimuli responsive BLA neurons within select ensembles can vary based on ensemble definition. The BLA nociceptive ensemble (~24% total neurons) was based on the union of all neurons responsive to nociceptive stimuli, this number was reduced when looking at neurons that respond to nociceptive and no other stimuli (~6% total neurons) or those that respond to all nociceptive stimuli (4–6%). 3397 [normal] and 7535 [neuropathic and uninjured] neurons from 9 mice with 3–4 [normal] and 5–7 [neuropathic or uninjured] sessions each. (**H**) Stimuli responsive BLA ensembles from animals run through experimental protocol in **Fig. S3A, C** are slightly more spatially related than the general population (orange) before and after injury or sham. Centroid locations for 3397 (normal), 3783 (neuropathic), and 3752 (uninjured) neurons were computed from PCA-ICA spatial filters and the Euclidean distance calculated to estimate the cumulative and probability densities, see *Supplemental Methods*. (**I**) Same as (H) indicating that stimuli-specific BLA ensembles in animals from experimental protocol **Fig. S3B** exhibit slightly more spatial relatedness in uninjured and neuropathic states as compared to the general neuronal population (n = 2839 [8 mice, 22 sessions] and 3625 [8 mice, 26 sessions] neurons for normal and neuropathic, respectively).

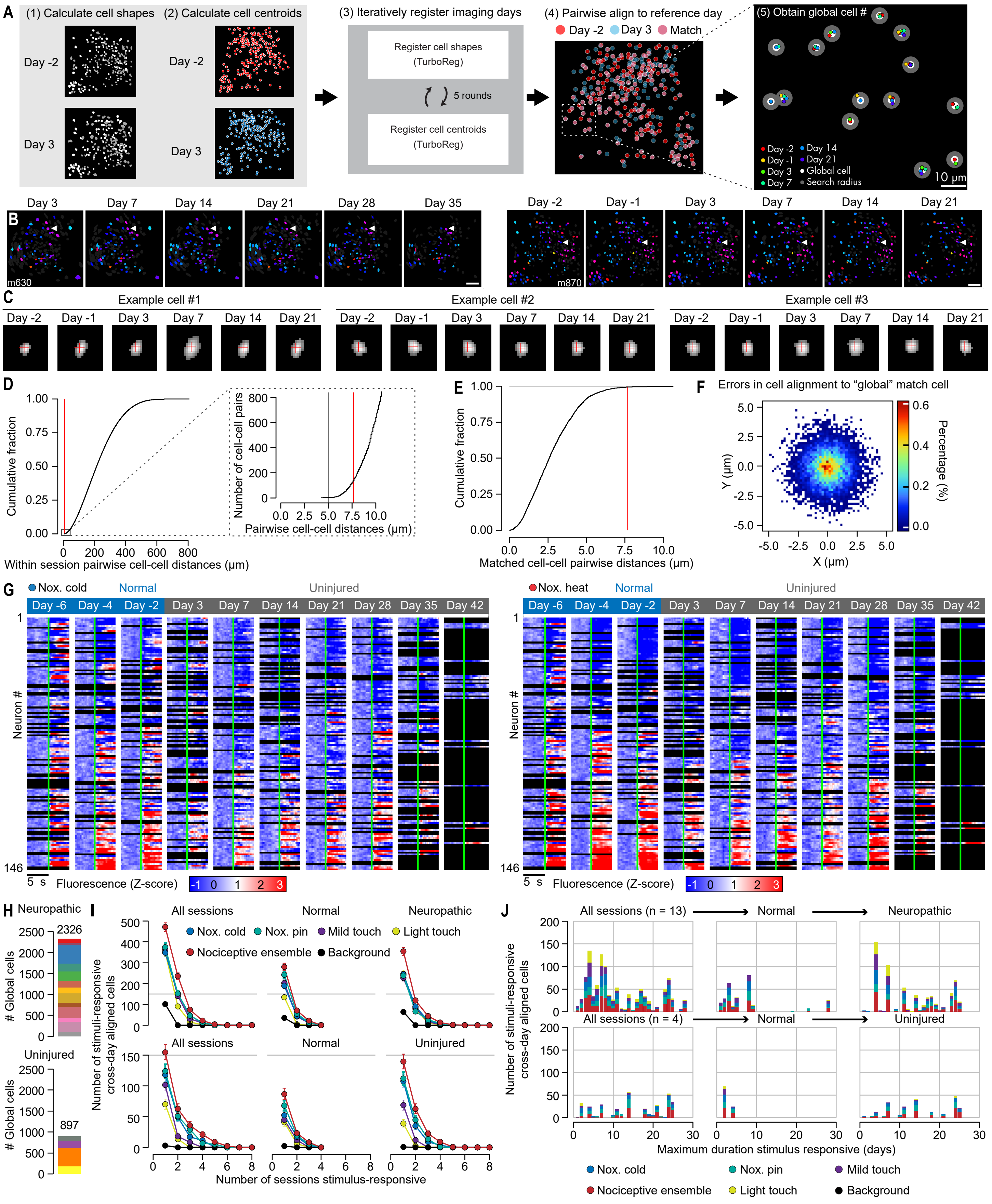
A
(1) Calculate cell shapes
(2) Calculate cell centroids
Day -2
Day 3
(3) Iteratively register imaging days
Register cell shapes (TurboReg)
5 rounds
Register cell centroids (TurboReg)
(4) Pairwise align to reference day
Day -2 Day 3 Match
(5) Obtain global cell #
Day -2 Day -1 Day 3 Day 7 Day 14 Day 21 Global cell Search radius
10 μm
B
Day 3 Day 7 Day 14 Day 21 Day 28 Day 35
m630
Day -2 Day -1 Day 3 Day 7 Day 14 Day 21
m870
C
Example cell #1
Example cell #2
Example cell #3
Day -2 Day -1 Day 3 Day 7 Day 14 Day 21
D
Cumulative fraction
Within session pairwise cell-cell distances (μm)
Number of cell-cell pairs
Pairwise cell-cell distances (μm)
E
Cumulative fraction
Matched cell-cell pairwise distances (μm)
F
Errors in cell alignment to "global" match cell
Y (μm)
X (μm)
Percentage (%)
G
Nox. cold Normal Uninjured
Nox. heat Normal Uninjured
Day -6 Day -4 Day -2 Day 3 Day 7 Day 14 Day 21 Day 28 Day 35 Day 42
Neuron #
1
146
5 s
Fluorescence (Z-score)
H Neuropathic
Global cells
2326
Uninjured
897
I
All sessions Normal Neuropathic
Nox. cold Nox. pin Mild touch Light touch
Nociceptive ensemble Background
All sessions Normal Uninjured
Number of stimuli-responsive cross-day aligned cells
Number of sessions stimulus-responsive
J
All sessions (n = 13) Normal Neuropathic
All sessions (n = 4) Normal Uninjured
Number of stimuli-responsive cross-day aligned cells
Maximum duration stimulus responsive (days)
Nox. cold Nox. pin Mild touch
Nociceptive ensemble Light touch Background

Fig. S6. Associate data for Fig. 1. BLA neuron stimulus-responsive ensembles are stable across days. (**A**) Method for cross-day alignment of BLA neural ensembles using real data from an example mouse. Day -2 and 3 are with respect to nerve injury surgery day. After neurons had been matched (steps 4 and 5), they were associated with a *global cell* that was then used to analyze their responses across days. See *Supplemental Methods* for detailed procedures. (**B**) Example neuron spatial filter maps showing cross-day alignment for two example mice's imaging sessions. Global cells matched across at least 70% of the imaging sessions are coded by a unique color. White arrow points to a neuron active across all aligned days for that animal. Scale bars, 100 µm. (**C**) Cross-day matched neurons showing similar spatial positioning for three example neurons from the right mouse in (B). Red crosses are neuron centroid locations, see *Supplemental Methods* for details of calculation. (**D**) Pairwise centroid Euclidean distances for all imaging sessions across mice (n = 17) showing that the vast majority of neurons are >10 µm apart. Inset, zoomed in view showing the absolute number of neuron pairs within 10 µm of one another. Red line indicates 0.01th percentile. Grey line indicates threshold used to group neurons in (A) into a global cell. (**E**) Same calculation as in (D) except restricted to neuron-neuron pairs within the same global cell, demonstrating the majority of neuron matches assigned to the same global cell are less than 5 µm apart. Red line is at the same location as the 99.99th percentile in (D) inset. (**F**) Individual neuron distances from their respective global cell centroid location if they were matched to another neuron on at least one other session (n = 13,558 session neurons). (**G**) Example animal showing all global cells (n = 146) that were active during greater than half of that animal's imaging sessions. A subset of neurons (bottom rows) are stimulus responsive to noxious cold (acetone) or heat (55°C water) across multiple imaging sessions and days to weeks of time. Black sections indicate sessions in which no associated neuron was found for that global cell. (**H**) Number of cross-day global cells across both neuropathic (n = 13 mice, n = 2,326 global cells) and uninjured control (n = 4 mice, 897 global cells) groups. Colors denote individual animals. These same neurons are used for analysis in (I-J). (**I**) Indicates number of global cells that significantly coded for indicated stimuli (see **Fig. 1** and **S5**) across either one or more imaging sessions irrespective of temporal distance separating imaging sessions. Grey line is 150 global cells and is common across the neuropathic (*top row*) and uninjured (*bottom row*) imaging groups. "Nociceptive ensemble" stimuli is a global cell that responded to either noxious pin and/or noxious cold on any given imaging session. (**J**) To determine how long global cells coded for specific stimuli (color coded), actual imaging session dates were used to calculate the maximum duration a global cells was found to be stimulus responsive. Of the 3,223 global cells matched across two or more imaging sessions, ~11% (350 global cells) responded to noxious stimuli with at least a week separating their first and final noxious stimuli responses

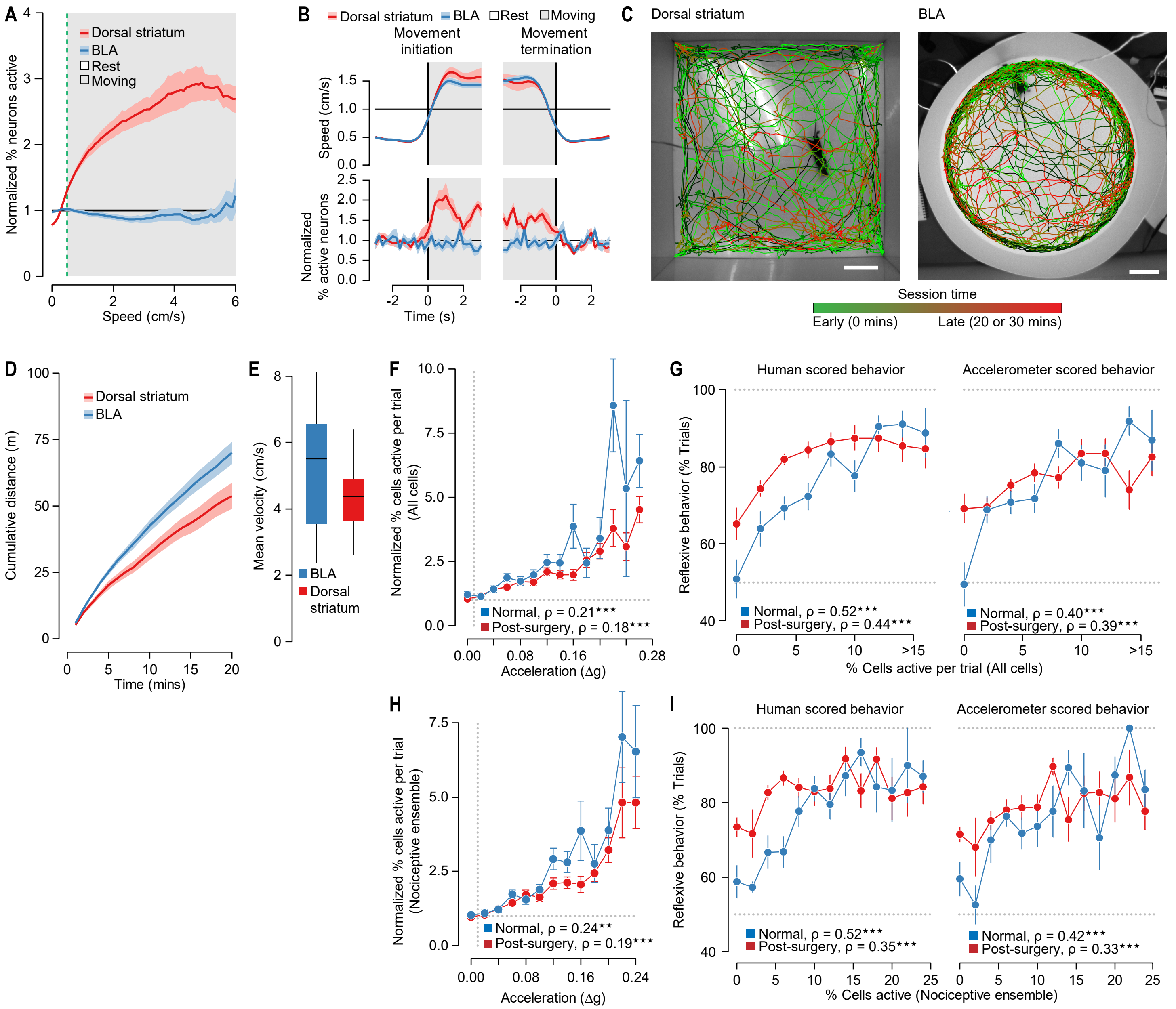

A
Dorsal striatum
BLA
Rest
Moving
Normalized % neurons active
Speed (cm/s)
B
Movement initiation
Movement termination
Speed (cm/s)
Normalized % active neurons
Time (s)
C
Dorsal striatum
BLA
Session time
Early (0 mins)
Late (20 or 30 mins)
D
Cumulative distance (m)
Time (mins)
E
Mean velocity (cm/s)
F
Normalized % cells active per trial (All cells)
Normal, ρ = 0.21***
Post-surgery, ρ = 0.18***
Acceleration (Δg)
G
Human scored behavior
Accelerometer scored behavior
Reflexive behavior (% Trials)
Normal, ρ = 0.52***
Post-surgery, ρ = 0.44***
Normal, ρ = 0.40***
Post-surgery, ρ = 0.39***
% Cells active per trial (All cells)
H
Normalized % cells active per trial (Nociceptive ensemble)
Normal, ρ = 0.24**
Post-surgery, ρ = 0.19***
Acceleration (Δg)
I
Human scored behavior
Accelerometer scored behavior
Reflexive behavior (% Trials)
Normal, ρ = 0.52***
Post-surgery, ρ = 0.35***
Normal, ρ = 0.42***
Post-surgery, ρ = 0.33***
% Cells active (Nociceptive ensemble)

Fig. S7. Associate data for Fig. 1 and 3. BLA neural activity is correlated with increased motivated escape behaviors, but not general movement. (**A**) Both dorsomedial striatum- (DMS) (red, n = 9 mice, 13 sessions total) and BLA-implanted (blue, n = 9 mice, 3–4 sessions each) animals freely explored either a square (60.96 × 60.96 cm) or circular (60.96 cm diameter) open field for 20–30 min. For each frame in a trial, we calculated the corresponding Ca^{2+} event-based population activity, the mean taken over specific velocity bins, and the final curve normalized by the mean velocity between 0 and 0.5 cm/s (threshold for movement). DMS, but not BLA, neuron activity showed a modulation in firing rate with velocity. (**B**) DMS- (red, n = 4 mice, 1 session each) and BLA-implanted (blue, n = 9 mice, 3–4 sessions each) animals freely explored an open field as in (A). Both animal speed (top) and population activity (bottom, normalized to 2 to 3 seconds before motion onset) were aligned to onset and offset of motion (see *Supplemental Methods*). Both groups showed similar movement initiation and termination behavior but only the DMS's neuron activity was modulated by start and stop of movement. (**C**) Example centroid positions of DMS - (left) and BLA-implanted (right) mice during free exploration in their respective open field setups. Early (green) and late (red) session times indicate continuous sampling of the environments throughout the session. Scale bars, 10 cm. (**D**) Cumulative distance traveled by DMS- (n = 9, 1 session each, red) and BLA-implanted (n = 9, 3–4 sessions each, blue) mice as run in (A). (**E**) Mean session velocity for both DMS- (n = 9, 1 session each, red) and BLA-implanted (n = 9, 3–4 sessions each, blue) mice. (**F**) Unlike during general movement in (A), increasingly vigorous responses to sensory (noxious cold, heat, and pin and mild and light touch) or "Approach/No contact" stimuli modulated population BLA activity (Spearman's ρ = [0.21, 0.18], p = [<0.001, <0.001] for [no injury, injury] cases). Graph shows the means ± SEM for population response at various levels of animal movement on a per trial basis as recorded from an accelerometer during pain trials as in **Fig. 1E, G, and M** and protocol in **Fig. S3A, C**. Neuron activity was normalized to trials with less than 0.01 *g* acceleration (the same acceleration threshold as used to indicate no response in (G), also see **Fig. S4D**) within each animal's session across all stimuli. Three stars, $P < 0.001$ (Spearman's). (**G**) The human manually scored and accelerometer calculated reflexive responses compared to % of all neurons activated by the same set of stimuli as in (F). Per trial responses were pooled across animals and the mean animal session response is plotted. They showed significantly increased responses as a larger fraction of the BLA ensemble was activated. For [normal, post-surgery (both neuropathic and uninjured)] cases human scored Spearman's ρ = [0.52, 0.44], p-value = [< 0.001, <0.001]: accelerometer, ρ = [0.40, 0.39], p-value = [< 0.001, < 0.001]. Error bars, ± SEM. 6815 trials, n = 9 mice, 3–4 (normal) and 5–7 (neuropathic/uninjured) sessions each. Three stars, $P < 0.001$ (Spearman's). (**H** to **I**) Same as (F) and (G) above except the population has been restricted to the nociceptive ensemble instead of the total neuron population. Error bars, ± SEM. 6815 trials, n = 9 mice, 3–4 (normal) and 5–7 (neuropathic/uninjured) sessions each. Three stars, $P < 0.001$ (Spearman's).

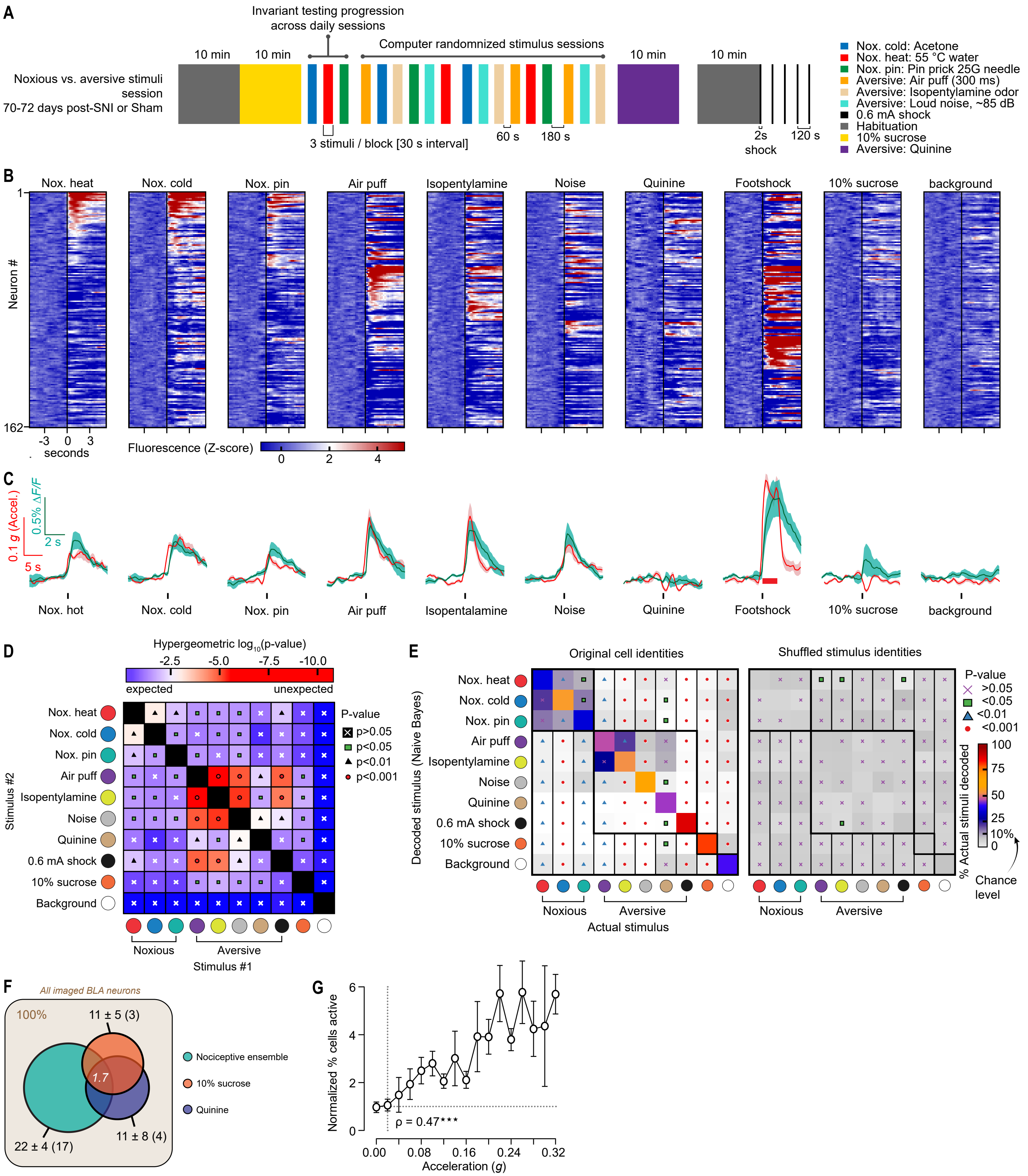
A
Invariant testing progression across daily sessions
Computer randomnized stimulus sessions
10 min
10 min
10 min
10 min
Noxious vs. aversive stimuli session
70-72 days post-SNI or Sham
3 stimuli / block [30 s interval]
60 s
180 s
2s shock
120 s
Nox. cold: Acetone
Nox. heat: 55 °C water
Nox. pin: Pin prick 25G needle
Aversive: Air puff (300 ms)
Aversive: Isopentylamine odor
Aversive: Loud noise, ~85 dB
0.6 mA shock
Habituation
10% sucrose
Aversive: Quinine
B
Nox. heat
Nox. cold
Nox. pin
Air puff
Isopentylamine
Noise
Quinine
Footshock
10% sucrose
background
Neuron #
seconds
Fluorescence (Z-score)
C
0.1 g (Accel.)
0.5% ΔF/F
2 s
5 s
Nox. hot
Isopentalamine
D
Hypergeometric log10(p-value)
expected
unexpected
P-value
p>0.05
p<0.05
p<0.01
p<0.001
Stimulus #2
Stimulus #1
0.6 mA shock
Background
Noxious
Aversive
E
Original cell identities
Shuffled stimulus identities
Decoded stimulus (Naive Bayes)
Actual stimulus
% Actual stimuli decoded
Chance level
F
All imaged BLA neurons
100%
11 ± 5 (3)
1.7
22 ± 4 (17)
11 ± 8 (4)
Nociceptive ensemble
10% sucrose
Quinine
G
Normalized % cells active
Acceleration (g)
ρ = 0.47***

Fig. S8. Associate data for Fig. 1. BLA nociceptive ensemble overlaps with, but is distinguishable from, aversive ensembles. (**A**) To compare BLA neuron responses to noxious and aversive stimuli, we adapted our protocol in **Fig. S3C** to study the response of animals to noxious (noxious heat, cold, and pin), five commonly used aversive (air puff [to the face], isopentylamine [odor], loud noise [~85 dB], quinine [bitter taste], and 0.6 mA footshock), and a positive valence (10% sucrose) stimuli. (**B**) Mean stimulus response across all trials for all BLA neurons during a single imaging session in an uninjured mouse (n = 162 neurons). Neuron identifications across different stimuli are consistent, demonstrating that some neurons encode multiple different types of noxious and aversive stimuli, while a separate neuron population uniquely encodes nociception (see **Fig. 1J**). The first three stimuli (noxious heat, cold, and pin) are considered noxious, the next five are aversive (air puff, isopentylamine, noise, quinine, and footshock), 10% sucrose is positive valence, and background is a negative control showing average response during random trial time points at least 10 seconds away from any defined stimuli. (**C**) Temporal dynamics of the mean $\Delta F/F$ of neurons within the nociceptive ensemble (cyan) and mean affective escape acceleration (red) for all imaging sessions and mice (n = 6 mice, 1 session each). (**D**) Probability of expecting overlap between two stimuli given total and stimuli responsive number of neurons was calculated using the hypergeometric distribution (see **Fig. S9C-E** and *Supplemental Methods*). Lower p-values (red) indicate the given overlap between stimuli was less likely to be due to chance. N = 6 mice, 1 session each. Symbols indicate various p-value thresholds using the same values used to color code the diagrams. (**E**) We constructed naïve Bayes decoders as described in **Fig. S9A** and applied them to the noxious vs. aversive stimuli experiments (n = 6 mice, 1 session each). The procedure, color coding, and symbols are as described in **Fig. S9B**, and see *Supplemental Methods*. The decoder tended to incorrectly predict one noxious stimuli as another based on the population activity but not the other aversive stimuli. Air puff and isopentylamine may have a high degree of neural ensemble overlap due to a similar method of stimulus delivery. Shuffled stimuli identities indicates that all trends are eliminated when the decoder was trained with the incorrect stimulus labels. (**F**) The nociceptive ensemble, as defined in **Fig 1H**, show less overlap with consummatory stimuli (10% sucrose and quinine) as compared to those stimuli with one another. (**G**) Increased response to either noxious or aversive stimuli (as defined in (A)) was predictive of amount of neural activity in the BLA (Spearman's $\rho = 0.47$, $p < 0.001$). Analysis same as in **Fig. S7F, H**.

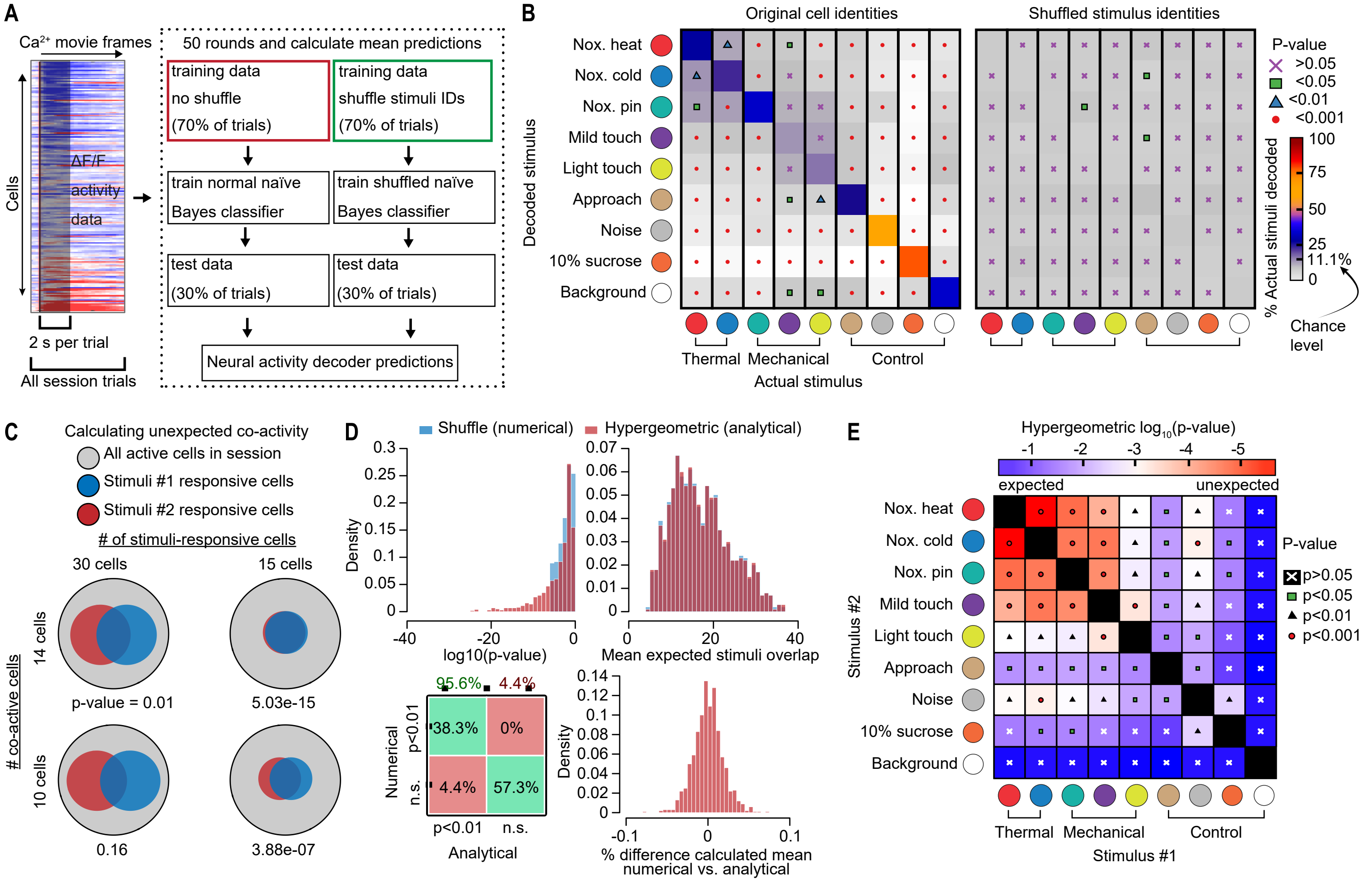

A
Ca²⁺ movie frames
Cells
ΔF/F
activity
data
2 s per trial
All session trials
50 rounds and calculate mean predictions
training data
no shuffle
(70% of trials)
training data
shuffle stimuli IDs
(70% of trials)
train normal naïve
Bayes classifier
train shuffled naïve
Bayes classifier
test data
(30% of trials)
test data
(30% of trials)
Neural activity decoder predictions
B
Original cell identities
Shuffled stimulus identities
Decoded stimulus
Nox. heat
Nox. cold
Nox. pin
Mild touch
Light touch
Approach
Noise
10% sucrose
Background
Thermal
Mechanical
Control
Actual stimulus
P-value
>0.05
<0.05
<0.01
<0.001
% Actual stimuli decoded
100
75
50
25
11.1%
0
Chance level
C
Calculating unexpected co-activity
All active cells in session
Stimuli #1 responsive cells
Stimuli #2 responsive cells
# of stimuli-responsive cells
30 cells
15 cells
co-active cells
14 cells
10 cells
p-value = 0.01
5.03e-15
0.16
3.88e-07
D
Shuffle (numerical)
Hypergeometric (analytical)
Density
log10(p-value)
Mean expected stimuli overlap
95.6%
4.4%
38.3%
0%
4.4%
57.3%
Numerical
p<0.01
n.s.
Analytical
% difference calculated mean numerical vs. analytical
E
Hypergeometric log10(p-value)
expected
unexpected
Stimulus #2
Stimulus #1
P-value
p>0.05
p<0.05
p<0.01
p<0.001

Fig. S9. Associate data for Fig. 1. BLA stimuli ensembles overlap and exhibit combinatorial coding of nociceptive information. (**A**) To test out the specificity of the neuronal ensemble dynamics between stimuli, we constructed a nine-way naïve Bayes decoder. For cross-validation, we split data each round 70:30 between training and test datasets using 2 seconds from each trial. After training the decoder, it was run on the test neuron activity data and the predicted stimuli state compared to the actual stimuli delivered (see (B)). The decoder was run through 50 rounds subsampling different sets of trials for use in training and test datasets. (**B**) The naïve Bayes decoder constructed in (A) was applied to sessions from **Fig. 1** (n = 9 mice, 3–4 sessions each). The decoder was then run on neural activity data for a new subset of stimuli and the actual stimuli at those frames compared to those predicted. We then normalized each actual stimuli column by the number of total actual stimuli to allow comparisons of how accurate the decoder was. Better performance (red/orange) occurred on noise and 10% sucrose than on innocuous (light purple) or noxious (blue and dark purple) stimuli. Symbols in the off-diagonal indicate whether prediction of correct stimuli was significantly higher than prediction of that stimuli (Wilcoxon sign-rank, Benjamini–Hochberg). Shuffled matrix indicates that all trends are eliminated when the decoder was trained with the incorrect stimuli labels. (**C**) How unexpected the overlap was in neurons activated by two stimuli depended on the total number of neurons activated by each stimuli, the amount of co-active neurons, and the total number of neurons. To quantify this, either a numerical (shuffling neuron identities and seeing how often they overlap) or exact analytical (using the hypergeometric distribution) solution can be used (see *Supplemental Methods*). Circles indicate numbers of neurons with gray circles indicating the total population. Number of stimuli activated neurons (red and blue circles) and number of co-activated neurons is the same in columns and rows, respectively. The hypergeometric distribution p-values are shown below each example of stimuli population overlaps. (**D**) To validate the use of the hypergeometric distribution, we ran 1,000 rounds of 1,000,000 shuffles for the estimated numerical distribution. Using the same number of total neurons, stimuli #1 responsive neurons, stimuli #2 responsive neuron, and overlap neurons, we also calculated the p-values, mean, and standard deviation using the hypergeometric distribution. For the mean and standard deviation the numerical and analytical solutions were not significantly different (Wilcoxon sign rank, $p = 0.68$ and 0.37 for mean and standard deviation). Paired difference in predicted mean were small (bottom right histogram), likely owing to precision error in numerical calculations since only 1 million shuffles were used. For the p-values, we found a high degree of agreement (95.6%) between overlap identified as significant by numerical and analytical methods (e.g. unexpected given the input parameters). (**E**) Probability of expecting overlap between two stimuli given total and stimuli responsive number of neurons was calculated using the hypergeometric distribution, see (C-D) and *Supplemental Methods*. Lower p-values (red) indicate the given overlap between stimuli was less likely to be due to chance. All the noxious stimuli are significantly unexpected in the amount of overlap with one another (top left). N = 9 mice, 3–4 sessions each. Symbols indicate various p-value thresholds using the same values used to color code the diagrams.

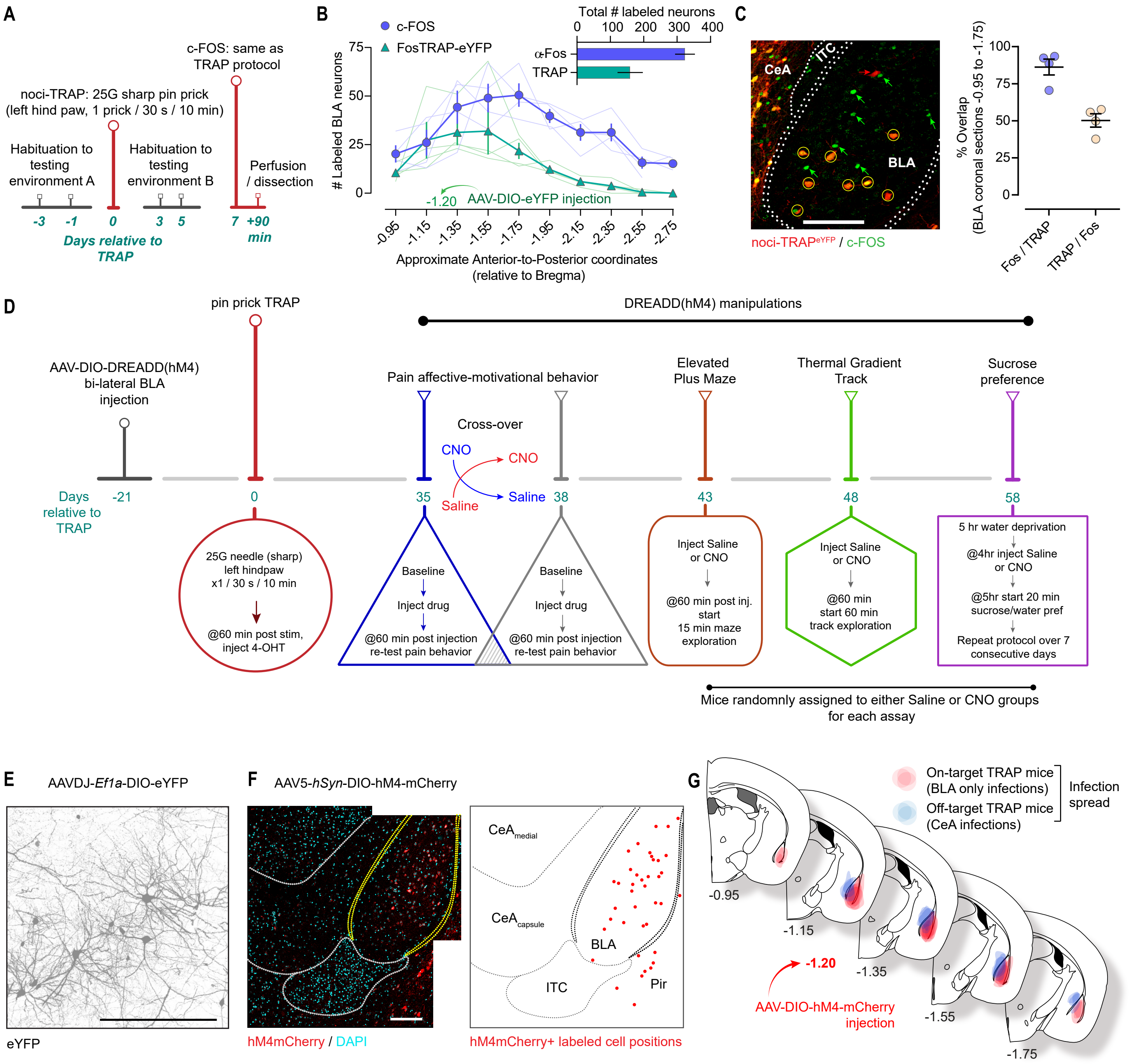
A
c-FOS: same as TRAP protocol
noci-TRAP: 25G sharp pin prick (left hind paw, 1 prick / 30 s / 10 min)
Habituation to testing environment A
Habituation to testing environment B
Perfusion / dissection
-3 -1 0 3 5 7 +90 min
Days relative to TRAP
B
c-FOS
FosTRAP-eYFP
Labeled BLA neurons
Total # labeled neurons
αFos
TRAP
-1.20 AAV-DIO-eYFP injection
Approximate Anterior-to-Posterior coordinates (relative to Bregma)
C
CeA
ITC
BLA
noci-TRAPeYFP / c-FOS
% Overlap (BLA coronal sections -0.95 to -1.75)
Fos / TRAP
TRAP / Fos
D
pin prick TRAP
DREADD(hM4) manipulations
AAV-DIO-DREADD(hM4) bi-lateral BLA injection
Pain affective-motivational behavior
Elevated Plus Maze
Thermal Gradient Track
Sucrose preference
Cross-over
CNO
CNO
Saline
Saline
Days relative to TRAP
-21 0 35 38 43 48 58
25G needle (sharp) left hindpaw x1 / 30 s / 10 min
@60 min post stim, inject 4-OHT
Baseline
Inject drug
@60 min post injection re-test pain behavior
Inject Saline or CNO
@60 min post inj. start 15 min maze exploration
@60 min start 60 min track exploration
5 hr water deprivation
@4hr inject Saline or CNO
@5hr start 20 min sucrose/water pref
Repeat protocol over 7 consecutive days
Mice randomnly assigned to either Saline or CNO groups for each assay
E
AAVDJ-Ef1a-DIO-eYFP
eYFP
F
AAV5-hSyn-DIO-hM4-mCherry
hM4mCherry / DAPI
CeAmedial
CeAcapsule
BLA
ITC
Pir
hM4mCherry+ labeled cell positions
G
On-target TRAP mice (BLA only infections)
Off-target TRAP mice (CeA infections)
Infection spread
-0.95 -1.15 -1.20 -1.35 -1.55 -1.75
AAV-DIO-hM4-mCherry injection

Fig. S10. Associate data for Fig. 2. Chemogenetic manipulation of the BLA nociceptive ensemble. (**A**) Experimental timeline for the noci-TRAP and nociceptive pin prick c-FOS immunohistochemical protocols. (**B**) Anterior to posterior BLA quantification of noci-TRAP^{eYFP} and c-FOS. (**C**) Representative image and quantification of anterior BLA noci-TRAP^{eYFP} neurons that were re-activated (c-FOS+) following a second pin prick stimulation 7 days later. (**D**) Experimental timeline for noci-TRAP for hM4-mCherry expression and subsequent behavioral testing. (**E**) Representative image of noci-TRAP neurons filled with eYFP. Note the highly branched architecture. (**F**) Representative image of precise expression of hM4-mCherry in the BLA but not in the neighboring central amygdala nucleus (CeA). Expanded view of same image as in **Fig. 2B**. Coordinates and structure demarcations from the mouse brain atlas of the Allen Institute for Brain Science. (**G**) Anatomical maps displaying the area of hM4-mCherry expression across the anterior-posterior amygdala in noci-TRAP mice. The AAV-*hSyn*-DIO-mCherry was injected at the A-P coordinate, -1.20 mm. On every brain slice illustration each red overlay shows the approximate medial-lateral spread of hM4-mCherry expressing neurons for an individual noci-TRAP mouse with a successful on-target TRAP (i.e. only BLA neurons were TRAP'd); the A-P spread for each mouse is illustrated across the different coordinate brain slices (n = 7 mice). The blue overlays on each brain slice indicate mice with off-target TRAP outside the BLA, primarily in the CeA. Based on this criteria n = 7 mice were excluded from the data set in **Fig. 2**.

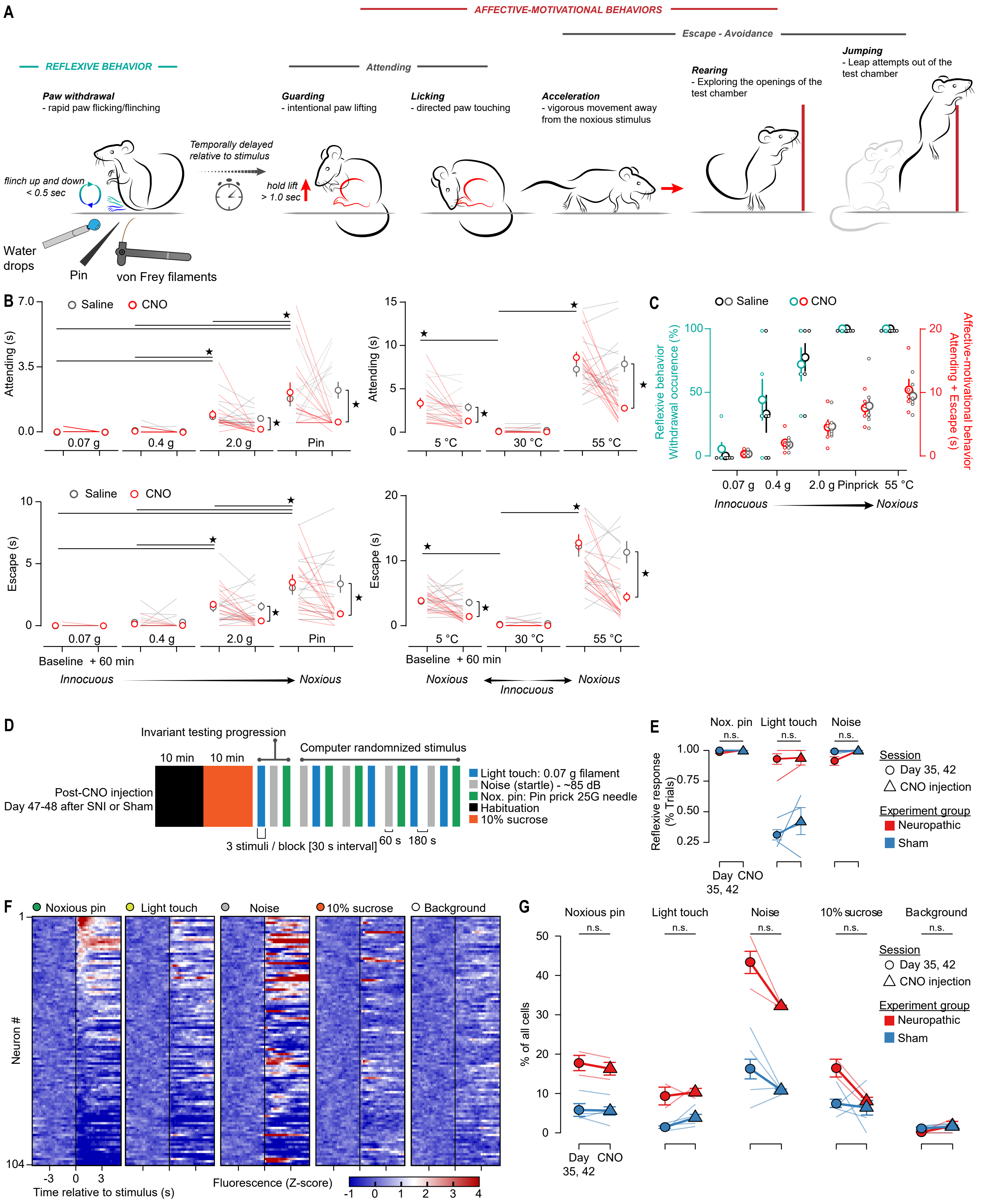

A
AFFECTIVE-MOTIVATIONAL BEHAVIORS
Escape - Avoidance
REFLEXIVE BEHAVIOR
Attending
Paw withdrawal
- rapid paw flicking/flinching
Guarding
- intentional paw lifting
Licking
- directed paw touching
Acceleration
- vigorous movement away from the noxious stimulus
Rearing
- Exploring the openings of the test chamber
Jumping
- Leap attempts out of the test chamber
flinch up and down < 0.5 sec
Temporally delayed relative to stimulus
hold lift > 1.0 sec
Water drops
Pin
von Frey filaments
B
Saline
CNO
Attending (s)
Escape (s)
0.07 g
0.4 g
2.0 g
Pin
5 °C
30 °C
55 °C
Baseline + 60 min
Innocuous
Noxious
C
Saline
CNO
Reflexive behavior Withdrawal occurence (%)
Affective-motivational behavior Attending + Escape (s)
0.07 g 0.4 g 2.0 g Pinprick 55 °C
Innocuous
Noxious
D
Invariant testing progression
Computer randomized stimulus
10 min
10 min
Post-CNO injection
Day 47-48 after SNI or Sham
Light touch: 0.07 g filament
Noise (startle) - ~85 dB
Nox. pin: Pin prick 25G needle
Habituation
10% sucrose
3 stimuli / block [30 s interval]
60 s
180 s
E
Nox. pin
Light touch
Noise
n.s.
Reflexive response (% Trials)
Day 35, 42
CNO
Session
Day 35, 42
CNO injection
Experiment group
Neuropathic
Sham
F
Noxious pin
Light touch
Noise
10% sucrose
Background
Neuron #
Time relative to stimulus (s)
Fluorescence (Z-score)
G
Noxious pin
Light touch
Noise
10% sucrose
Background
n.s.
% of all cells
Day 35, 42
CNO
Session
Day 35, 42
CNO injection
Experiment group
Neuropathic
Sham

Fig. S11. Associate data for Fig. 2. Chemogenetic separation of affective-motivational pain behaviors from reflexive responses. (**A**) Illustrative progression of pain behaviors, from the immediate reflexes to the temporally delayed affective-motivational behaviors, following delivery of a noxious stimulus. (**B**) Same mice as in **Fig. 2F** and **2G** are shown, but we display here the separate scores for subcategories of affective-motivational behaviors: attending (top rows) or escape behavior (bottom rows). N = 14 mice / group. (**C**) Lack of effect of clozapine-N-oxide (CNO, 10 mg/kg) on reflexive (left green y-axis) or affective-motivational (right red y-axis) behaviors in control mice expressing eYFP in the BLA nociceptive ensemble (AAVDJ-*Ef1α*-DIO-eYFP). N = 6 mice/group. (**D**) To compare BLA neuron responses before and after CNO application, we adapted our protocol in **Fig. S3C** to study the responses of animals 30 min after CNO (10 mg/kg) injection. Mice were then tested on a simplified version of protocol in **Fig. S3C**. (**E**) Lack of behavioral effect of CNO ($F(1,60) = 0.016$, $p = 0.90$, One-way ANOVA) and absence of statistical interaction between CNO and stimulus ($F(2,60) = 0.14$, $p = 0.87$, Two-way ANOVA). N = 6 mice, 1 session each, n.s. = no significant difference before and after CNO injection (Wilcoxon rank-sum). (**F**) Mean stimulus response across all trials for all BLA neurons during a single imaging session in an uninjured mouse (n = 104 neurons). Neuron identification across different stimuli are consistent, demonstrating that some neurons encode different types of stimuli. Stimuli are the same as in **Fig. S5B**. (**G**) Mean neural response to various stimuli on sessions where we did not (Day 35, 42) or did (CNO) inject mice with CNO (10 mg/kg). CNO did not alter neural responses, $F(1,178) = 1.002$, $p = 0.318$, One-way ANOVA pooled over groups and stimuli. n.s. = no significant difference before and after CNO injection (Wilcoxon rank-sum). Stars, $P < 0.05$. Overlaid small dots and lines are individual subjects. Large dots represent group mean responses and error bars show ± SEM.

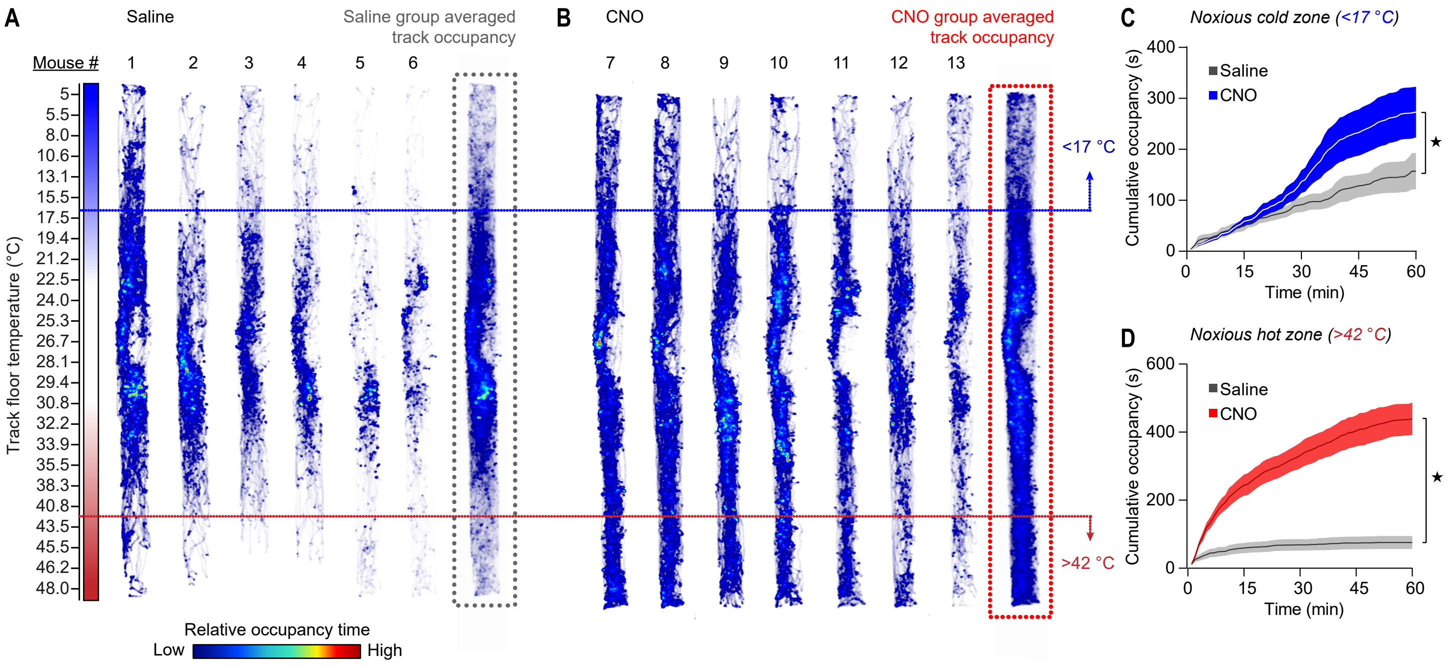

Fig. S12. Associate data for Fig. 2. Thermal track occupancy. (**A**) Individual trial and group average occupancy paths for noci-TRAP^{hM4} mice treated with saline. (**B**) Individual trial and group average occupancy paths for noci-TRAP^{hM4} mice treated with CNO. (**C** and **D**) Cumulative occupancy inside the (C) noxious cold or (D) noxious hot zones for noci-TRAP^{hM4} mice treated with either saline or CNO. Stars, $P < 0.05$, Kolmogorov-Smirnov test.

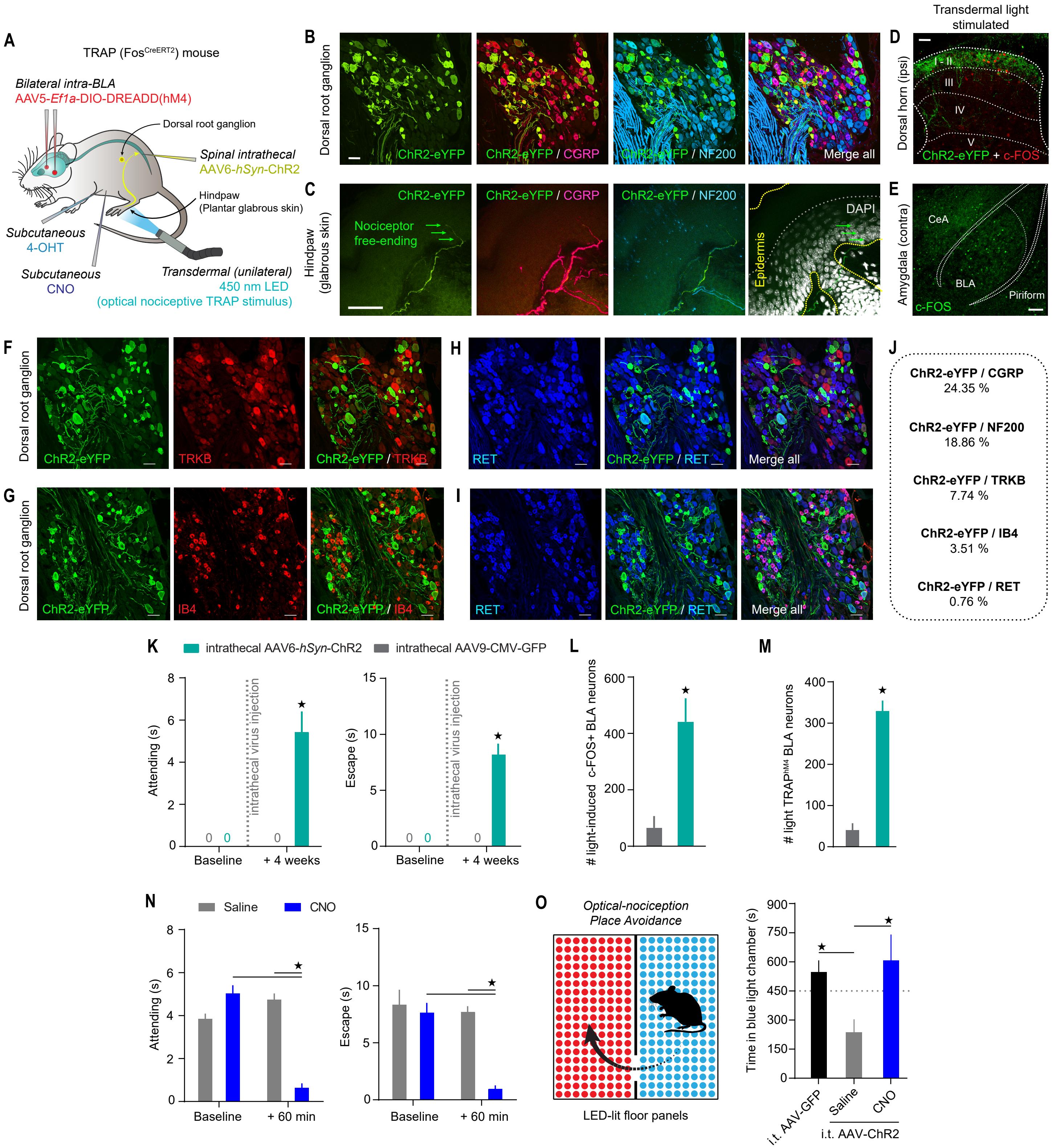
A
TRAP (Fos^CreERT2) mouse
Bilateral intra-BLA
AAV5-Ef1a-DIO-DREADD(hM4)
Dorsal root ganglion
Spinal intrathecal
AAV6-hSyn-ChR2
Hindpaw
(Plantar glabrous skin)
Subcutaneous
4-OHT
Subcutaneous
CNO
Transdermal (unilateral)
450 nm LED
(optical nociceptive TRAP stimulus)
B
Dorsal root ganglion
ChR2-eYFP
ChR2-eYFP / CGRP
ChR2-eYFP / NF200
Merge all
C
Hindpaw (glabrous skin)
Nociceptor free-ending
DAPI
Epidermis
D
Transdermal light stimulated
Dorsal horn (ipsi)
I - II
III
IV
V
ChR2-eYFP + c-FOS
E
Amygdala (contra)
CeA
BLA
Piriform
c-FOS
F
TRKB
ChR2-eYFP / TRKB
G
IB4
ChR2-eYFP / IB4
H
RET
ChR2-eYFP / RET
I
J
ChR2-eYFP / CGRP
24.35 %
ChR2-eYFP / NF200
18.86 %
ChR2-eYFP / TRKB
7.74 %
ChR2-eYFP / IB4
3.51 %
ChR2-eYFP / RET
0.76 %
K
intrathecal AAV6-hSyn-ChR2
intrathecal AAV9-CMV-GFP
Attending (s)
Escape (s)
intrathecal virus injection
Baseline
+ 4 weeks
L
light-induced c-FOS+ BLA neurons
M
light TRAP^hM4 BLA neurons
N
Saline
CNO
+ 60 min
O
Optical-nociception Place Avoidance
LED-lit floor panels
Time in blue light chamber (s)
i.t. AAV-GFP
Saline
CNO
i.t. AAV-ChR2

Fig. S13. Associate data for Fig. 2. Optogenetic activation of nociceptors elicits pain affective-motivational behaviors that require the BLA nociceptive ensemble. (**A**) Optogenetic nociceptive TRAP of hM4-mCherry in the BLA (o-TRAP^{hM4}). Cre-dependent AAV5-*hSyn*-DIO-hM4-mCherry was stereotaxically injected into the bilateral BLA of TRAP mice, followed by a spinal intrathecal injection of AAV6-*hSyn*-ChR2(H134R)-eYFP to infect peripheral dorsal root ganglion nociceptors. After 3–4 weeks, transdermal blue light was applied to the left hindpaw to activate the light-sensitive cation channel ChR2 in nociceptors (TRAP stimulus), which was followed by injection of 4-OHT 60 min later to induce DNA recombination and expression of hM4-mCherry in the BLA. Behavioral tests take place 3–5 weeks later. (**B**) Expression of ChR2-eYFP in peripheral CGRP+ nociceptors of dorsal root ganglia. (**C**) ChR2-eYFP was trafficked to the cutaneous terminals of CGRP+ nociceptors. These free nerve endings innervate the epidermis of the glabrous skin where they can be activated by transdermal 450 nm light. (**D**) The central terminals of ChR2-eYFP+ nociceptors innervate the substantia gelatinosa of the spinal cord dorsal horn. Repeated transdermal light stimulations (1 s; 2–3 mW/mm^2) induce FOS expression in dorsal horn neurons within the terminal fields of ChR2-eYFP+ nociceptors, indicating transmission of nociceptive information to the CNS. (**E**) Transdermal optogenetic nociception drives FOS expression in the BLA. (**F** to **J**) Additional immunohistochemical characterization of the peripheral afferent populations expressing viral ChR2-eYFP. ChR2-eYFP was predominately expressed in peptidergic CGRP+ nociceptors, and mostly excluded from the IB4+/Ret+ non-peptidergic nociceptor populations. (**K**) Optogenetic nociception elicits pain affective-motivational behaviors, such as attending and escape, similar to natural noxious stimuli. There was no effect of transdermal light on behavioral responses before expression of ChR2 or in mice expressing GFP in nociceptors. (**L**) Quantification of FOS expression in the BLA induced by optogenetic nociception. (**M**) Quantification of hM4-mCherry expression in the BLA following o-TRAP. (**N**) CNO-mediated silencing of the BLA nociceptive ensemble reduces attending and escape behaviors in response to noxious transdermal light stimuli. (**O**) (*Left*) Optical real-time place escape avoidance task in which one chamber floor was illuminated by a LED array red or blue light. (*Right*) Control mice expressing GFP in nociceptors show no preference between either chambers. ChR2-eYFP+ nociceptor mice given a saline injection significantly avoid the blue light chamber. CNO treatment (10 mg/kg) in a separate group of ChR2-eYFP+ nociceptor mice eliminates the aversion to the noxious blue light chamber. Panels B-J, n = 3 mice. Panels K-N, n = 5 mice / group; K,N, RM Two-way ANAOVA + Bonferroni; L,M, Student's t Test. Panel O, n = 4 mice / group; One-way ANAOVA + Bonferroni. Stars, $P < 0.05$ for all panels. Error bars, ± SEM. In (E), scale bar, 100 μm. All other scale bars, 50 μm. ChR2, Channelrhodopsin2; eYFP, enhanced Yellow Fluorescent Protein; CGRP, Calcitonin Gene-Related Peptide; NF200, Neurofilament 200; TRKB, Tropomyosin receptor kinase B; IB4, Isolectin B4; RET, Proto-oncogene tyrosine-protein kinase receptor Ret.

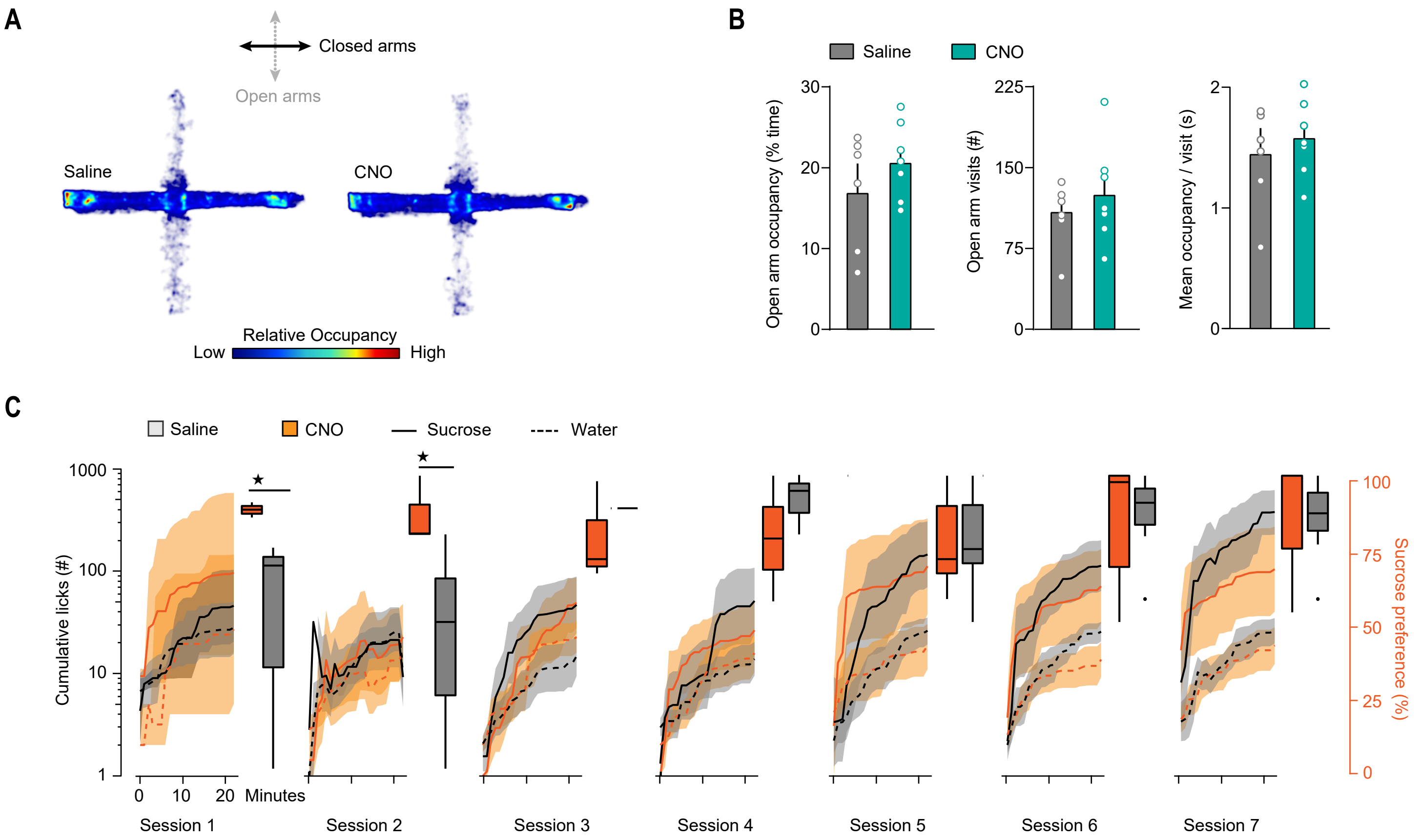

Fig. S14. Associate data for Fig. 2. Anxiety-like and incentive motivational behavior. (**A**) Temporal exploration paths on an elevated plus maze. Group mean occupancy path traces for noci-TRAP^{hM4} mice given either saline or CNO (10 mg/kg). (**B**) No effect of CNO-mediated silencing of the BLA nociceptive ensemble on the total distance traveled, or average velocity of mice in the Elevated Plus Maze. Overlaid dots are individual subjects. Error bars, ± SEM. (**C**) Daily incentive approach behavior during exposure to an unconditioned sucrose-water preference task. Left: Daily cumulative lick bouts at either a 10% sucrose port or a water port over a 7 day trail period. noci-TRAP^{hM4} mice were given either saline or CNO 60 min prior to the start of the daily trial. Right: Daily consumption of water and sucrose displayed as a % sucrose preference over water. Boxplots display the 1st, 2nd, and 3rd quartiles with whiskers indicating 1.5*IQR. Stars, $P < 0.05$. *Note that on the first and second days (Sessions 1 and 2) of conditioning the noci-TRAPhM4 mice treated with CNO displayed a significant sucrose preference over mice treated with saline.*

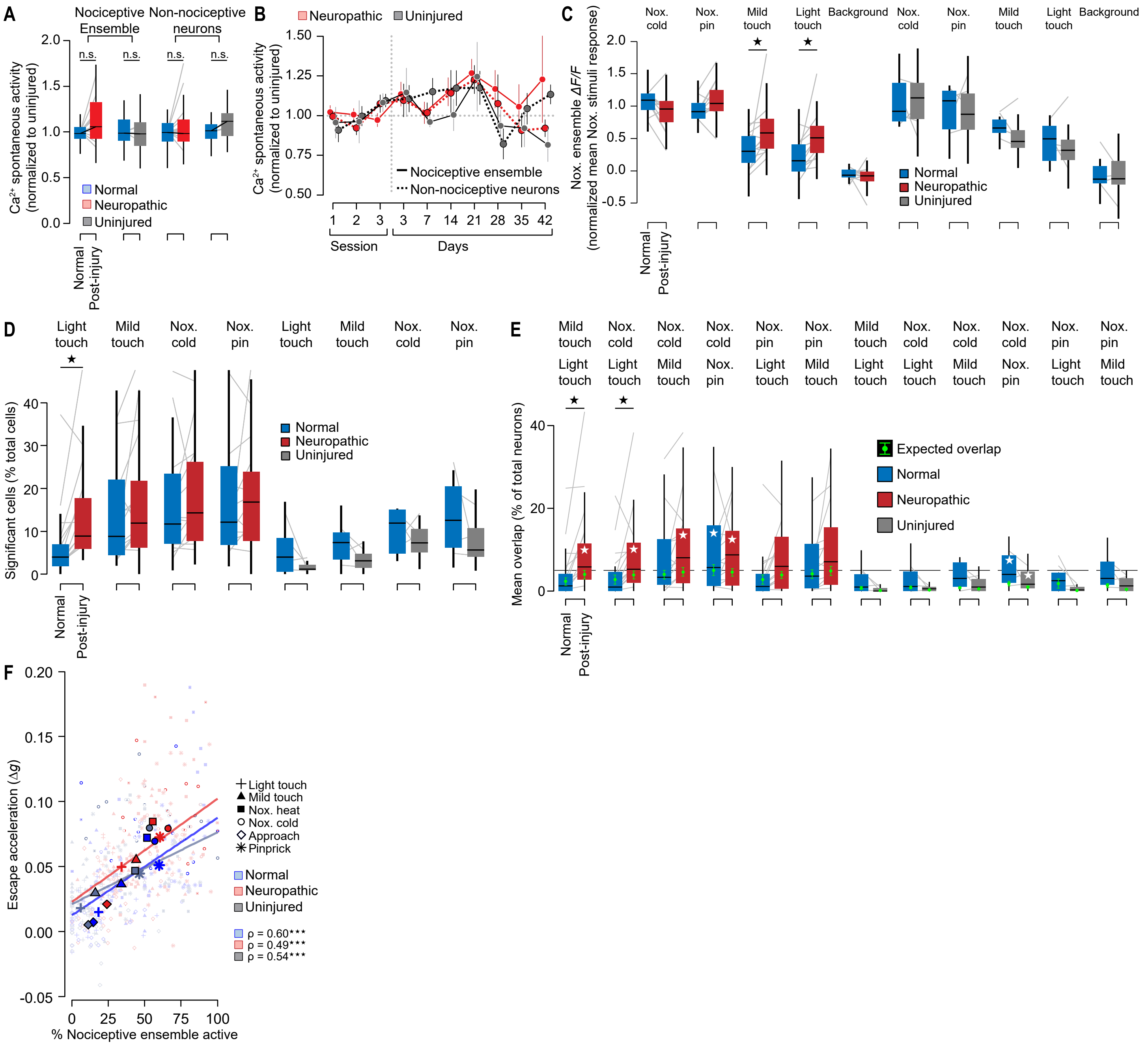
A
Nociceptive Ensemble
Non-nociceptive neurons
n.s.
Ca2+ spontaneous activity (normalized to uninjured)
Normal
Neuropathic
Uninjured
Normal
Post-injury
B
Neuropathic
Uninjured
Ca2+ spontaneous activity (normalized to uninjured)
Nociceptive ensemble
Non-nociceptive neurons
Session
Days
C
Nox. cold
Nox. pin
Mild touch
Light touch
Background
Nox. ensemble ΔF/F (normalized mean Nox. stimuli response)
Normal
Neuropathic
Uninjured
D
Light touch
Mild touch
Nox. cold
Nox. pin
Significant cells (% total cells)
Normal
Neuropathic
Uninjured
E
Mean overlap (% of total neurons)
Expected overlap
Normal
Neuropathic
Uninjured
F
Escape acceleration (Δg)
% Nociceptive ensemble active
Light touch
Mild touch
Nox. heat
Nox. cold
Approach
Pinprick
Normal
Neuropathic
Uninjured
ρ = 0.60***
ρ = 0.49***
ρ = 0.54***

Fig. S15. Associate data for Fig. 3. BLA spontaneous activity and neuron ensemble activity and overlap after nerve injury. (**A**) There were no significant spontaneous activity increases or decreases in the nociceptive or non-nociceptive neuron populations after spared nerve injury (p = 0.11 [nociceptive] and 0.58 [non-nociceptive], Wilxocon rank-sum, n = 13 mice) or in uninjured mice (p = 0.82 [nociceptive] and 0.13 [non-nociceptive], Wilxocon rank-sum, n = 4 mice). Spontaneous BLA neuron activity was measured during a 10 to 15 min habituation period that either took place in a small chamber separate from pain experiments (n = 8 mice) or within the test chamber itself (n = 9 mice). We calculated the BLA Ca^{2+} transient rate (see *Supplemental Methods*) for each session and the rates for each animal normalized by the mean session activity across all sessions before spared nerve injury or sham surgery. Individual gray lines indicate mean per animal changes in BLA Ca^{2+} transient rate before and after spared nerve injury or sham surgery. (**B**) Same as in (A), showing the spontaneous BLA Ca^{2+} transient activity across imaging sessions before and after spared nerve injury (red) or sham surgery (gray). For post-SNI days 28, 35, and 42, n = 9 mice from experimental protocol in **Fig. S3A, C** are included compared to a combination of all mice (n = 17) across both pre-SNI sessions and days 3–21 post-SNI. (**C**) Mean session population $\Delta F/F$ activity for neurons within the noxious ensemble (either 5°C [n = 2 mice] or acetone [n = 15 mice] and pin prick) before and after spared nerve injury normalized to noxious cold and pin $\Delta F/F$ on a per animal session basis. Innocuous (mild and light touch) stimuli showed a significant increase in activity specifically in nerve injured animals (Wilcoxon rank-sum, Benjamini–Hochberg corrected). Individual gray lines indicate mean per animal changes in $\Delta F/F$ response before and after injury or sham surgery. Stars, $P < 0.001$. (**D**) Percentage of significantly responding neurons to noxious and touch stimuli before and after nerve injury or sham surgery. Light touch stimuli show a significant increase specifically in injured animals. Stars, $P < 0.001$, Wilcoxon rank-sum test. Gray lines indicate individual animal mean % of neurons activated. (**E**) Light touch neural ensemble had a more unexpected overlap with noxious cold (5°C water or acetone) ensemble after spare nerve injury (n = 13) but not in uninjured control animals (n = 4). The mean overlap between pairs of stimuli ensembles is indicated by boxplots while the green dot indicates the median ± [1st and 3rd quartiles] expected overlap between stimuli (calculated from hypergeometric distribution, see **Fig. S9C-E** and *Supplemental Methods*). Gray lines are individual animals before and after spared nerve injury or sham surgery. Significant change in overlap regardless of expectedness is indicated by bar connecting "no injury" to SNI while white star within boxplot indicates significant difference from expected mean overlap. Significance calculated using Wilcoxon rank-sum with Benjamini–Hochberg multiple-comparisons correction. (**F**) Similar plot as in **Fig. 3H**. Correlation between % of nociceptive ensemble activated and escape acceleration per imaging session (light colored points) and across animal groups and conditions (dark, larger points) show significant correlation (Spearman's ρ = 0.60 [Normal], 0.49 [Neuropathic] and 0.54 [Uninjured]). Three stars, $P < 0.001$.

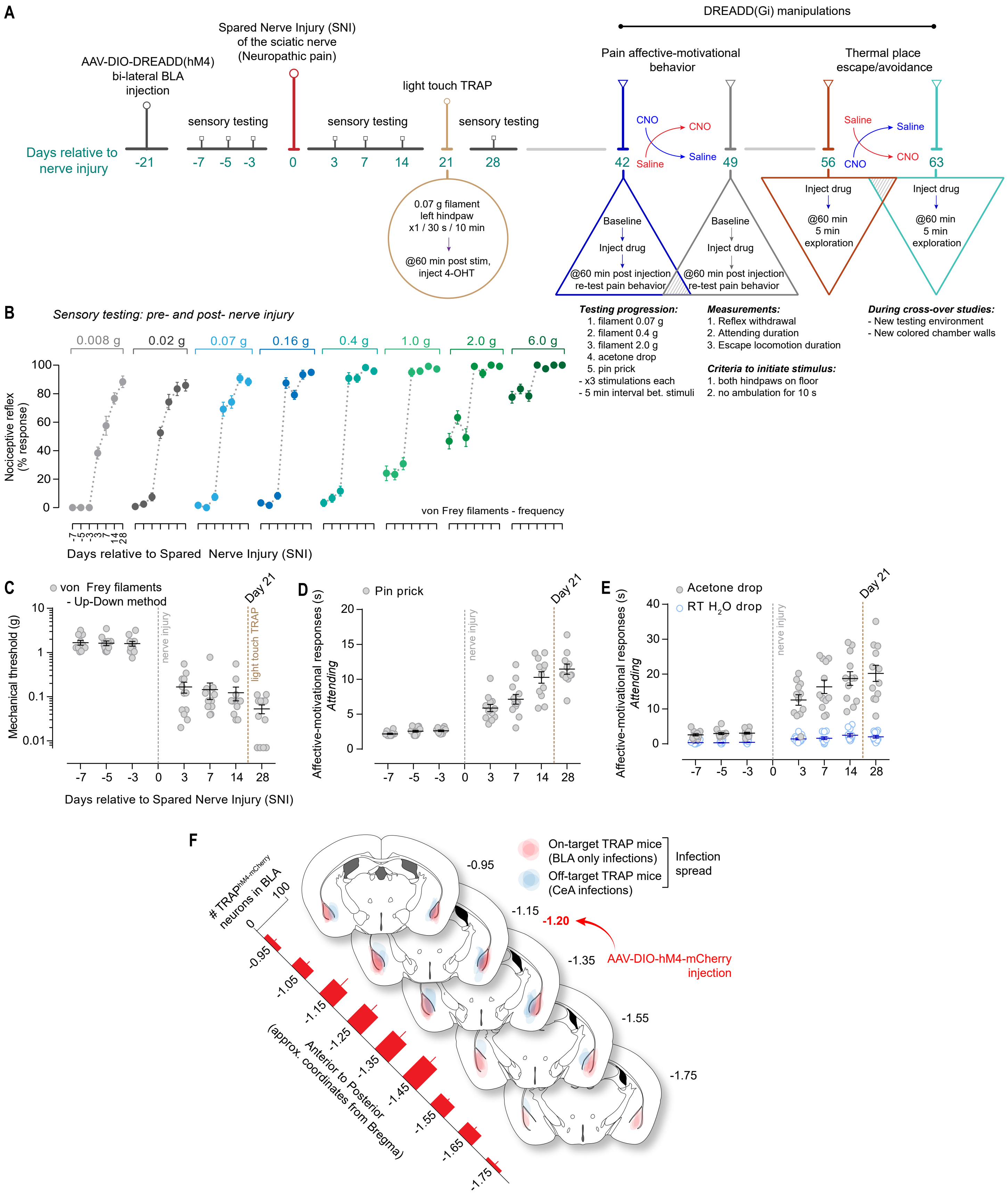
A
DREADD(Gi) manipulations
Spared Nerve Injury (SNI) of the sciatic nerve (Neuropathic pain)
AAV-DIO-DREADD(hM4) bi-lateral BLA injection
light touch TRAP
sensory testing
Pain affective-motivational behavior
Thermal place escape/avoidance
Days relative to nerve injury
-21 -7 -5 -3 0 3 7 14 21 28 42 49 56 63
CNO
Saline
0.07 g filament left hindpaw x1 / 30 s / 10 min
@60 min post stim, inject 4-OHT
Baseline
Inject drug
@60 min post injection re-test pain behavior
@60 min 5 min exploration
Testing progression:
1. filament 0.07 g
2. filament 0.4 g
3. filament 2.0 g
4. acetone drop
5. pin prick
- x3 stimulations each
- 5 min interval bet. stimuli
Measurements:
1. Reflex withdrawal
2. Attending duration
3. Escape locomotion duration
Criteria to initiate stimulus:
1. both hindpaws on floor
2. no ambulation for 10 s
During cross-over studies:
- New testing environment
- New colored chamber walls
B
Sensory testing: pre- and post- nerve injury
0.008 g 0.02 g 0.07 g 0.16 g 0.4 g 1.0 g 2.0 g 6.0 g
Nociceptive reflex (% response)
von Frey filaments - frequency
Days relative to Spared Nerve Injury (SNI)
C
von Frey filaments - Up-Down method
Mechanical threshold (g)
nerve injury
light touch TRAP
Day 21
D
Pin prick
Affective-motivational responses (s) Attending
E
Acetone drop
RT H_2O drop
F
TRAP^hM4-mCherry neurons in BLA
Anterior to Posterior (approx. coordinates from Bregma)
On-target TRAP mice (BLA only infections)
Off-target TRAP mice (CeA infections)
Infection spread
AAV-DIO-hM4-mCherry injection
-0.95 -1.15 -1.20 -1.35 -1.55 -1.75

Fig. S16. Associate data for Fig. 4. Strategy to manipulate BLA ensembles involved in chronic pain affect. (**A**) Experimental and behavioral testing timeline for light touch-TRAP of DREADD(hM4)-mCherry in neuropathic mice with mechanical allodynia. (**B** and **C**) Nociceptive mechanical sensitivity before and after sciatic nerve injury, quantified by (B) withdrawal frequency to intensifying von Frey filament stimulations and by (C) mechanical thresholds using the Up-Down method. (**D** and **E**) Pain affective-motivational hyperalgesia in response to (D) pin prick and (E) acetone drop. (**F**) Anatomical maps displaying the area of hM4-mCherry expression across the anterior-posterior amygdala in light touch-TRAP mice. The AAV-*hSyn*-DIO-mCherry was injected at the A-P coordinate, -1.20 mm. On every brain slice illustration each red overlay shows the approximate medial-lateral spread of hM4-mCherry expressing neurons for an individual light touch-TRAP mouse with a successful on-target TRAP (i.e. only BLA neurons were TRAP'd); the A-P spread for each mouse is illustrated across the different coordinate brain slices (n = 7 mice). The blue overlays on each brain slice indicate mice with off-target TRAP outside the BLA, primarily in the CeA. Based on this criteria n = 5 mice were excluded from the data set in **Fig. 4**. The underlying histogram displays the means ± SEM. quantification of light touch-TRAP^{hM4} neurons along the anterior-posterior axis of the BLA (n = 7 on-target TRAP mice).

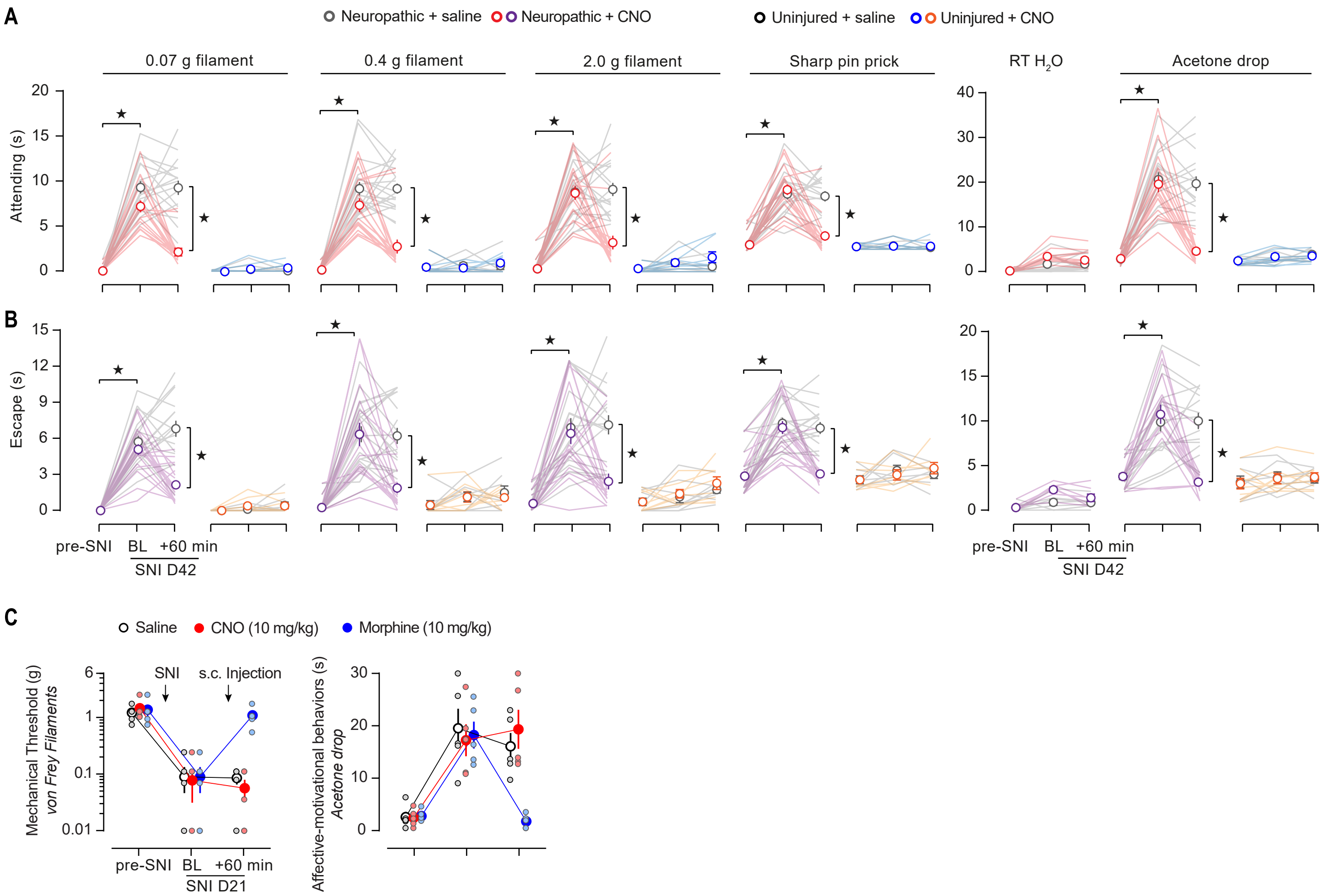

Fig. S17. Associate data for Fig. 4. Chemogenetic reduction in chronic pain affect. (**A** to **B**) Same mice as in **Fig. 4E**, but displaying the separate scores for different subcategories of affective-motivational behaviors; attending (B,D) or escape (C,E), in mice with nerve injury (B,C) or without injury (D,E). (**C**) Lack of effect of CNO (10 mg/kg) on reflex behaviors elicited by stimulation with von Frey filaments (left panel) or on affective-motivational behaviors in response to an acetone drop (right panel) in control neuropathic mice (i.e. not expressing hM4-mCherry in the BLA). Morphine served as a positive control and reduced both reflexive and affective-motivational pain behaviors. Stars, $P < 0.05$. Overlaid lines or small dots are individual subjects. Large dots represent group mean responses and error bars show ± SEM.

Table 1. Summary of the percent of stimuli responsive basolateral amygdala neurons in neuropathic and sham groups

Stimulus	Mice	Group	% of total cells		% of nociceptive ensemble		% of nociceptive ensemble as function of % total cells	
			Uninjured	Neuropathic	Uninjured	Neuropathic	Uninjured	Neuropathic
Noxious heat (~55°C water)	Fig. S3A, C[2]	Neuropathic	13 ± 2	18 ± 2	56 ± 8	53 ± 4	13 ± 2	18 ± 2
Noxious cold (~5°C water or Acetone)	Fig. S3A, C[2]	Neuropathic	15 ± 2	22 ± 3	60 ± 5	66 ± 3	15 ± 2	22 ± 3
Noxious pin (Pin prick)	Fig. S3A, C[2]	Neuropathic	16 ± 3	19 ± 2	61 ± 6	59 ± 3	16 ± 3	19 ± 2
Mild touch (2.0 g filament)	Fig. S3A, C[2]	Neuropathic	12 ± 2	17 ± 2	36 ± 5	42 ± 3	9 ± 2	15 ± 2
**Light touch (0.07 g filament)**	**Fig. S3A, C[2]**	**Neuropathic**	**7 ± 2**	**14 ± 2**	**21 ± 4**	**33 ± 3**	**6 ± 1**	**11 ± 2**
Approach ("Miss hit" or no contact)	Fig. S3A, C[2]	Neuropathic	9 ± 3	12 ± 2	20 ± 4	24 ± 3	4 ± 1	8 ± 2
Noise	Fig. S3A, C[2]	Neuropathic	37 ± 5	39 ± 4	68 ± 5	59 ± 4	17 ± 3	21 ± 3
10% sucrose	Fig. S3A, C[2]	Neuropathic	19 ± 4	17 ± 3	25 ± 6	20 ± 3	7 ± 2	7 ± 2
Background	Fig. S3A, C[2]	Neuropathic	0.1 ± 0.1	0.5 ± 0.2	0.3 ± 0.2	0.3 ± 0.2	0.1 ± 0.1	0.1 ± 0.1
Nociceptive ensemble[1]	Fig. S3A, C[2]	Neuropathic	25 ± 3	32 ± 2	100 ± 0	100 ± 0	25 ± 3	32 ± 2
Noxious heat (~55°C water)	Fig. S3C[3]	Sham	14 ± 4	9 ± 1	44 ± 9	47 ± 5	14 ± 4	9 ± 1
Noxious cold (~5°C water or Acetone)	Fig. S3C[3]	Sham	10 ± 1	10 ± 1	54 ± 8	51 ± 5	10 ± 1	10 ± 1
Pin prick	Fig. S3C[3]	Sham	15 ± 4	9 ± 1	56 ± 6	45 ± 5	15 ± 4	9 ± 1
Mild touch (2.0 g filament)	Fig. S3C[3]	Sham	7 ± 1	4 ± 1	24 ± 5	16 ± 3	6 ± 1	3 ± 1
**Light touch (0.07 g filament)**	**Fig. S3C[3]**	**Sham**	**7 ± 3**	**2 ± 0**	**15 ± 5**	**6 ± 1**	**5 ± 2**	**1 ± 0**
Approach ("Miss hit" or no contact)	Fig. S3C[3]	Sham	4 ± 1	5 ± 1	9 ± 3	10 ± 3	3 ± 1	2 ± 1
Noise	Fig. S3C[3]	Sham	28 ± 5	25 ± 3	60 ± 6	44 ± 5	14 ± 3	8 ± 1
10% sucrose	Fig. S3C[3]	Sham	16 ± 5	11 ± 2	26 ± 7	19 ± 4	8 ± 3	3 ± 1
Background	Fig. S3C[3]	Sham	0.4 ± 0.3	0.2 ± 0.1	0.1 ± 0.1	0.2 ± 0.2	0 ± 0	0 ± 0
Nociceptive ensemble[1]	Fig. S3C[3]	Sham	24 ± 4	18 ± 2	100 ± 0	100 ± 0	24 ± 4	18 ± 2
Noxious cold (Acetone)	Fig. S3B[5]	Neuropathic	20 ± 4	14 ± 2	63 ± 4	57 ± 4	20 ± 4	14 ± 2
Noxious pin (Pin prick)	Fig. S3B[5]	Neuropathic	21 ± 4	19 ± 4	66 ± 4	67 ± 4	21 ± 4	19 ± 4
Mild touch (2.0 g filament)	Fig. S3B[5]	Neuropathic	17 ± 3	18 ± 3	29 ± 5	37 ± 6	12 ± 3	13 ± 3
**Light touch (0.07 g filament)**	**Fig. S3B[5]**	**Neuropathic**	**10 ± 4**	**16 ± 3**	**14 ± 5**	**30 ± 6**	**7 ± 3**	**10 ± 2**
Nociceptive ensemble[4]	Fig. S3B[5]	Neuropathic	28 ± 4	25 ± 4	100 ± 0	100 ± 0	28 ± 4	25 ± 4

[1] Consist of cells responsive to 55°C water, 5°C water, Acetone, or Pin prick.

[2] N = 5 mice, analysis from 3 or 4 (uninjured) and 5 or 7 (neuropathic) sessions per mice. All values mean ± s.e.m.

[3] N = 4 mice, analysis from 3 (uninjured) and 7 (sham surgery) sessions per mice. All values mean ± s.e.m.

[4] Consist of cells responsive to Acetone and Pin prick.

[5] N = 8 mice, analysis from 22 (uninjured) and 26 (injured) total sessions pooled across all mice. All values mean ± s.e.m.

Table S1. Associated data for Fig. 1 and S3. Last two columns ("% of nociceptive ensemble as a function of % total neurons") are a measure of columns 5 and 6 ("% of nociceptive ensemble") neurons within the total population. **Fig. S3A, C** neuropathic mice have 1,779 neurons [5 mice, 3–4 sessions each] and 3,783 neurons [5 mice, 5–7 sessions each] from normal and neuropathic sessions, respectively. **Fig. S3C** sham mice have 1,618 neurons [4 mice, 3 sessions each] and 3,752 neurons [4 mice, 7 sessions each] from normal and uninjured sessions, respectively. **Fig S3B** neuropathic mice have n = 2,839 [8 mice, 22 total sessions] and 3,625 [8 mice, 26 total sessions] neurons from normal and neuropathic sessions, respectively

References and Notes

1. D. D. Price, Psychological and Neural Mechanisms of the Affective Dimension of Pain. *Science (80-.).* **288**, 1769–1772 (2000).
2. M. N. Baliki, A. V. Apkarian, Nociception, Pain, Negative Moods, and Behavior Selection. *Neuron*. **87**, 474–491 (2015).
3. C. Peirs, R. P. Seal, Neural circuits for pain: Recent advances and current views. *Science (80-.).* **354** (2016), doi:10.7554/eLife.22866.
4. L. Garcia-Larrea, H. Bastuji, Pain and consciousness. *Prog. Neuro-Psychopharmacology Biol. Psychiatry* (2017), doi:10.1016/j.pnpbp.2017.10.007.
5. P. H. Janak, K. M. Tye, From circuits to behaviour in the amygdala. *Nature*. **517**, 284–292 (2015).
6. N. Hebben, S. Corkin, H. Eichenbaum, K. Shedlack, Diminished ability to interpret and report internal states after bilateral medial temporal resection: case H.M. *Behav. Neurosci.* **99**, 1031–1039 (1985).
7. N. Neimann *et al.*, Familial Urbach-Wiethe Disease with indifference to pain. *Bull Soc Fr Dermatol Syphiligr*. **March**, 292–4 (1964).
8. M. Ploner, H. Freund, A. Schnitzler, Pain affect without pain sensation in a patient with a postcentral lesion. *Pain*. **81**, 211–4 (1999).
9. M. L. Uhelski, M. A. Davis, P. N. Fuchs, Pain affect in the absence of pain sensation : Evidence of asomaesthesia after somatosensory cortex lesions in the rat. *Pain*. **153**, 885–892 (2012).
10. M. Costigan, J. Scholz, C. J. Woolf, Neuropathic Pain: A Maladaptive Response of the Nervous System to Damage. *Annu. Rev. Neurosci.* **32**, 1–32 (2009).
11. V. Neugebauer, *Amygdala Pain Mechanisms* (2015), vol. 227.
12. P. Chang, M. Virginia, D. Procissi, A. Baria, A. V. Apkarian, Brain activity for tactile allodynia : a longitudinal awake rat functional magnetic resonance imaging study tracking emergence of neuropathic pain. **158**, 488–497 (2017).
13. L. E. Simons *et al.*, The responsive amygdala: Treatment-induced alterations in functional connectivity in pediatric complex regional pain syndrome. *Pain* (2014), doi:10.1016/j.pain.2014.05.023.
14. J. A. Hashmi *et al.*, Shape shifting pain: Chronification of back pain shifts brain representation from nociceptive to emotional circuits. *Brain*. **136**, 2751–2768 (2013).
15. F. Gore *et al.*, Neural Representations of Unconditioned Stimuli in Basolateral Amygdala Mediate Innate and Learned Responses. *Cell*. **162**, 134–145 (2015).
16. V. Neugebauer, W. Li, G. C. Bird, G. Bhave, R. W. Gereau, Synaptic plasticity in the amygdala in a model of arthritic pain: differential roles of metabotropic glutamate receptors 1 and 5. *J. Neurosci.* **23**, 52–63 (2003).
17. S. Han *et al.*, Elucidating an Affective Pain Circuit that Creates a Threat Memory Article Elucidating an Affective Pain Circuit that Creates a Threat Memory. *Cell*. **162**, 363–374 (2015).
18. G. Ji *et al.*, Cognitive impairment in pain through amygdala-driven prefrontal cortical deactivation. *J. Neurosci.* **30**, 5451–5464 (2010).
19. B. F. Grewe *et al.*, Neural ensemble dynamics underlying a long-term associative memory. *Nature*. **543**, 670–675 (2017).
20. Y. Ziv *et al.*, Long-term dynamics of CA1 hippocampal place codes SOM. *Nat. Neurosci.* **16**, 264–6 (2013).
21. J. G. Parker *et al.*, *Diametric neural ensemble dynamics in parkinsonian and dyskinetic states* (2018), vol. 557.
22. W. Zhang *et al.*, Functional circuits and anatomical distribution of response properties in the primate amygdala. *J. Neurosci.* **33**, 722–33 (2013).

23. P. Namburi *et al.*, A circuit mechanism for differentiating positive and negative associations. *Nature*. **520**, 675–678 (2015).

24. J. Kim, M. Pignatelli, S. Xu, S. Itohara, S. Tonegawa, Antagonistic negative and positive neurons of the basolateral amygdala. *Nat. Neurosci.*, 1–15 (2016).

25. C. Guenthner, K. Miyamichi, H. H. Yang, H. C. Heller, L. Luo, Permanent genetic access to transiently active neurons via TRAP: Targeted recombination in active populations. *Neuron*. **78**, 773–784 (2013).

26. B. L. Roth, DREADDs for Neuroscientists. *Neuron*. **89**, 683–694 (2016).

27. J. L. Gomez *et al.*, Chemogenetics revealed: DREADD occupancy and activation via converted clozapine. *Science (80-.).* **357**, 503–507 (2017).

28. G. G. Calhoon *et al.*, Acute Food Deprivation Rapidly Modifies Valence-Coding Microcircuits in the Amygdala. *bioRxiv* (2018), doi:10.1101/285189.

29. C. A. Von Hehn, R. Baron, C. J. Woolf, Deconstructing the Neuropathic Pain Phenotype to Reveal Neural Mechanisms. *Neuron*. **73**, 638–652 (2012).

30. B. W. Balleine, S. Killcross, Parallel incentive processing: an integrated view of amygdala function. *Trends Neurosci.* **29**, 272–279 (2006).

31. C. D. Salzman, S. Fusi, Emotion , Cognition , and Mental State Representation in Amygdala and Prefrontal Cortex. *Annu. Rev. Neurosci.* **33**, 173–202 (2010).

32. J. E. LeDoux, R. Brown, A higher-order theory of emotional consciousness. *Proc. Natl. Acad. Sci.* **114**, E2016–E2025 (2017).

33. N. Grahek, *Feeling Pain and Being in Pain* (MIT Press, 2007).

34. R. Melzack, K. Casey, in *The Skin Senses* (Charles C. Thomas, 1968), pp. 432–439.

35. F. Ramirez, J. M. Moscarello, J. E. LeDoux, R. M. Sears, Active Avoidance Requires a Serial Basal Amygdala to Nucleus Accumbens Shell Circuit. *J. Neurosci.* **35**, 3470–3477 (2015).

36. A. Burgos-robles *et al.*, Amygdala inputs to prefrontal cortex guide behavior amid conflicting cues of reward and punishment. *Nat. Neurosci.* (2017), doi:10.1038/nn.4553.

37. A. Beyeler *et al.*, Defined Neurons in the Basolateral Amygdala Article Organization of Valence-Encoding and Projection-Defined Neurons in the Basolateral Amygdala. *Cell Rep.* **22**, 905–918 (2018).

38. K. Wiech, Deconstructing the sensation of pain: The influence of cognitive processes on pain perception. *Science (80-.).* **354**, 584–587 (2016).

39. C. Torsney, A. B. MacDermott, Disinhibition Opens the Gate to Pathological Pain Signaling in Superficial Neurokinin 1 Receptor-Expressing Neurons in Rat Spinal Cord. *J. Neurosci.* **26**, 1833–1843 (2006).

40. P. A. Pizzo, N. M. Clark, O. Carter Pokras, *Relieving Pain in America: A Blueprint for Transforming Prevention, Care, Education, and Research* (2011).

41. N. D. Volkow, F. S. Collins, The Role of Science in Addressing the Opioid Crisis. *NEJM*. **377**, 391–394 (2017).

42. G. Corder, D. C. Castro, M. R. Bruchas, G. Scherrer, Endogenous and Exogenous Opioids in Pain. *Annu. Rev. Neurosci.* (2018), doi:10.1146/annurev-neuro-080317-061522.

43. P. Skolnick, N. D. Volkow, Re-energizing the Development of Pain Therapeutics in Light of the Opioid Epidemic. *Neuron*. **92**, 294–297 (2016).

44. F. Jelasic, Relation of the lateral part of the amygdala to pain. *Confin. Neurol.* **21**, 53–5 (1966).

45. B. T. Grosser, C. J. Woolf, G. A. Fitzgerald, Time for nonaddictive relief of pain. *Science (80-.).* **355**, 1026–1027 (2017).

46. B. Ahanonu, calciumImagingAnalysis: a software package for analyzing one- and two-photon calcium imaging datasets. *GitHub* (2018), doi:10.5281/ZENODO.2222295.

47. P. Jean-richard-dit-bressel, S. Killcross, G. P. Mcnally, Behavioral and neurobiological mechanisms of punishment : implications for psychiatric disorders. *Neuropsychopharmacology* (2018), doi:10.1038/s41386-018-0047-3.

48. R. Melzack, P. Wall, Pain Mechanisms: A New Theory. *Science (80-.).* **150**, 971–979 (1965).

49. M. J. Millan, The induction of pain: an integrative review. *Prog. Neurobiol.* **57**, 1–164 (1999).

50. G. Ekman, M. Frankenhauser, S. Levander, I. Mellis, The influence of intensity and duration of electrical stimulational on subjective variables. *Scand. J. Psychol.* **7**, 58–64 (1966).

51. T. Tashiro, A. Higashiyama, The perceptual properties of electrocutaneous stimulation : Sensory quality , subjective intensity , and intensity-duration relation. *Percept. Psychophys.* **30**, 579–586 (1982).

52. M. S. Fanselow, The postshock activity burst. *Anim. Learn. Behav.* **10**, 448–454 (1982).

53. H. Beaudry, I. Daou, A. Ase, A. Riberiro-da-Silva, P. Seguela, Distinct behavioral responses evoked by selective optogenetic stimulation of the major TRPV1 1 and MrgD 1 subsets of C-fibers. *Pain*. **158** (2017).

54. D. Terburg *et al.*, The Basolateral Amygdala Is Essential for Rapid Escape: A Human and Rodent Study. *Cell*. **175**, 723–735.e16 (2018).

55. J. Fadok *et al.*, A competitive inhibitory circuit for selection of active and passive fear responses. *Nat. Publ. Gr.* **542**, 96–100 (2017).

56. C. Gauriau, J. Bernard, Pain pathways and parabrachial circuits in the rat. *Exp. Physiol.* **87**, 251–8 (2002).

57. M. E. Carter, M. E. Soden, L. S. Zweifel, R. D. Palmiter, Genetic identification of a neural circuit that suppresses appetite. *Nature*. **503**, 111–4 (2013).

58. R. D. Palmiter, The Parabrachial Nucleus : CGRP Neurons Function as a General Alarm. *Trends Neurosci.* **41**, 280–293 (2018).

59. C. A. Campos, A. J. Bowen, C. W. Roman, R. D. Palmiter, Encoding of danger by parabrachial CGRP neurons. *Nature*. **555**, 617–620 (2018).

60. P. Tovote *et al.*, Midbrain circuits for defensive behaviour. *Nature*. **534**, 206–212 (2016).

61. T. Ozawa *et al.*, A feedback neural circuit for calibrating aversive memory strength. *Nat. Neurosci.* **20**, 90–97 (2016).

62. R. J. Fox, C. A. Sorenson, Bilateral lesions of the amygdala attenuate analgesia induced by diverse environmental challenges. *Brain Res.* **648**, 215–221 (1994).

63. W. Zhu, Z. Z. Pan, mu-Opioid-Mediated Inhibition of Glutamate Synaptic Transmission in Rat Central Amygdala Neurons. *Neuroscience*. **133**, 97–103 (2005).

64. M. A. Oliveira, W. A. Prado, Role of PAG in the antinociception evoked from the medial or central amygdala in rats. *Brain Res. Bull.* **54**, 55–63 (2001).

65. J. Annese *et al.*, Postmortem examination of patient H.M.'s brain based on histological sectioning and digital 3D reconstruction. *Nat Commun*. **5**, 3122 (2014).

66. P. Veinante, I. Yalcin, M. Barrot, The amygdala between sensation and affect: a role in pain. *J. Mol. Psychiatry*. **1**, 9 (2013).

67. P. Kyriazi, D. B. Headley, D. Pare, Multi-dimensional Coding by Basolateral Amygdala Neurons. *Neuron*. **99**, 1315–1328.e5 (2018).

68. T.-W. Chen *et al.*, Ultrasensitive fluorescent proteins for imaging neuronal activity. *Nature*. **499**, 295–300 (2013).

69. B. Slotnick, A Simple 2-Transistor Touch or Lick Detector Circuit. *J. Exp. Anal. Behav.* **91**, 253–255 (2009).

70. B. Solway, S. C. Bose, G. Corder, R. R. Donahue, B. K. Taylor, Tonic inhibition of chronic pain by neuropeptide Y. *Proc. Natl. Acad. Sci. U. S. A.* **108**, 7224–7229 (2011).

71. K. Kobayakawa *et al.*, Innate versus learned odour processing in the mouse olfactory bulb. *Nature*. **450**, 503–508

(2007).

72. C. M. Root, C. A. Denny, R. Hen, R. Axel, The participation of cortical amygdala in innate, odour-driven behaviour. *Nature*. **515**, 269–273 (2014).

73. L. R. Saraiva *et al.*, Combinatorial effects of odorants on mouse behavior. *Proc. Natl. Acad. Sci.* **113**, E3300–E3306 (2016).

74. Y. A. Blednov, B. F. Cravatt, S. L. Boehm, D. Walker, R. A. Harris, Role of endocannabinoids in alcohol consumption and intoxication: Studies of mice lacking fatty acid amide hydrolase. *Neuropsychopharmacology*. **32**, 1570–1582 (2007).

75. P. Thévenaz, U. E. Ruttimann, M. Unser, A pyramid approach to subpixel registration based on intensity. *IEEE Trans. Image Process.* **7**, 27–41 (1998).

76. E. A. Mukamel, A. Nimmerjahn, M. J. Schnitzer, Automated Analysis of Cellular Signals from Large-Scale Calcium Imaging Data. *Neuron*. **63**, 747–760 (2009).

77. Y. Benjamini, Y. Hochberg, Controlling the false discovery rate: a practical and powerful approach to multiple testing. *J. R. Stat. Soc. B*. **57** (1995), pp. 289–300.

78. M. J. Mathie, N. H. Lovell, A. C. F. Coster, B. G. Celler, Determining activity using a triaxial accelerometer. *Proc. Second Jt. 24th Annu. Conf. Annu. Fall Meet. Biomed. Eng. Soc.*, 2481–2482 (2002).

79. Y. Li *et al.*, Neuronal Representation of Social Information in the Medial Amygdala of Awake Behaving Mice. *Cell*. **171**, 1176–1179.e17 (2017).

80. S. M. Iyer *et al.*, Virally mediated optogenetic excitation and inhibition of pain in freely moving nontransgenic mice. *Nat. Biotechnol.* **32**, 274–278 (2014).

81. G. Corder *et al.*, Loss of μ-opioid receptor signaling in nociceptors, and not spinal microglia, abrogates morphine tolerance without disrupting analgesic efficacy. *Nat. Med.* **23**, 164–173 (2017).

82. A. Manglik *et al.*, Structure-based discovery of opioid analgesics with reduced side effects. *Nature*, 1–6 (2016).

83. C. J. Woolf, Long term alterations in the excitability of the flexion reflex produced by peripheral tissue injury in the chronic decerebrate rat. *Pain*. **18**, 325–343 (1984).

84. R. Blanchard, D. Blanchard, Passive and active reactions to fear-eliciting stimuli. *J Comp Physiol Psychol*. **68**, 129–135 (1969).

85. R. Bolles, Species-specific defense reactions and avoidance learning. *Psychol Rev*. **77**, 32–48 (1970).

86. R. Bolles, M. Fanselow, A perceptual-defensive-recuperative model of fear and pain. *Behav Brain Sci*. **3**, 291–323 (1980).

87. C. Darwin, *The expression of the emotions in man and animals* (Albemarle, London, 1872).

88. W. Estes, Discriminative conditioning; effects of a Pavlovian conditioned stimulus upon a subsequently established operant response. *J Exp Psychol*. **38**, 173–177 (1948).

89. W. Estes, B. Skinner, Some quantitative properties of anxiety. *J Exp Psychol*. **29**, 390–400 (1941).

90. J. S. Mogil, Animal models of pain: progress and challenges. *Nat. Rev. Neurosci.* **10**, 283–294 (2009).

91. R. Rescola, Pavlovian conditioned fear in Sidman avoidance learning. *J Comp Physiol Psychol*. **65**, 55–60 (1968).

92. B. Skinner, The behavior of organisms: an experimental analysis. *Appleton-CenturyCrofts, New York* (1938).

93. S. R. Chaplan, F. W. Bach, J. W. Pogrel, J. M. Chung, T. L. Yaksh, Quantitative assessment of tactile allodynia in the rat paw. *J. Neurosci. Methods*. **53**, 55–63 (1994).

94. G. Corder *et al.*, Constitutive μ-opioid receptor activity leads to long-term endogenous analgesia and dependence. *Science (80-.)*. **341**, 1394–1399 (2013).

References and Notes

1. D. D. Price, Psychological and neural mechanisms of the affective dimension of pain. *Science* **288**, 1769–1772 (2000). doi:10.1126/science.288.5472.1769 Medline

2. M. N. Baliki, A. V. Apkarian, Nociception, Pain, Negative Moods, and Behavior Selection. *Neuron* **87**, 474–491 (2015). doi:10.1016/j.neuron.2015.06.005 Medline

3. C. Peirs, R. P. Seal, Neural circuits for pain: Recent advances and current views. *Science* **354**, 578–584 (2016). doi:10.1126/science.aaf8933 Medline

4. L. Garcia-Larrea, H. Bastuji, *Prog. Neuropsychopharmacol. Biol. Psychiatry* (2017). 10.1016/j.pnpbp.2017.10.007

5. P. H. Janak, K. M. Tye, From circuits to behaviour in the amygdala. *Nature* **517**, 284–292 (2015). doi:10.1038/nature14188 Medline

6. N. Hebben, S. Corkin, H. Eichenbaum, K. Shedlack, Diminished ability to interpret and report internal states after bilateral medial temporal resection: Case H.M. *Behav. Neurosci.* **99**, 1031–1039 (1985). doi:10.1037/0735-7044.99.6.1031 Medline

7. N. Neimann, J. Beurey, M. Pierson, P. Tridon, J. Sapelier, P. Melin, Familial Urbach-Wiethe disease with indifference to pain. *Bull. Soc. Fr. Dermatol. Syphiligr.* **71**, 292–294 (1964). Medline

8. M. Ploner, H. J. Freund, A. Schnitzler, Pain affect without pain sensation in a patient with a postcentral lesion. *Pain* **81**, 211–214 (1999). doi:10.1016/S0304-3959(99)00012-3 Medline

9. M. L. Uhelski, M. A. Davis, P. N. Fuchs, Pain affect in the absence of pain sensation: Evidence of asomaesthesia after somatosensory cortex lesions in the rat. *Pain* **153**, 885–892 (2012). doi:10.1016/j.pain.2012.01.018 Medline

10. M. Costigan, J. Scholz, C. J. Woolf, Neuropathic pain: A maladaptive response of the nervous system to damage. *Annu. Rev. Neurosci.* **32**, 1–32 (2009). doi:10.1146/annurev.neuro.051508.135531 Medline

11. V. Neugebauer, Amygdala Pain Mechanisms. *Handb. Exp. Pharmacol.* **227,** 261–284 (2015). doi:10.1007/978-3-662-46450-2_13 Medline.

12. P. C. Chang, M. V. Centeno, D. Procissi, A. Baria, A. V. Apkarian, Brain activity for tactile allodynia: A longitudinal awake rat functional magnetic resonance imaging study tracking emergence of neuropathic pain. *Pain* **158**, 488–497 (2017). doi:10.1097/j.pain.0000000000000788 Medline

13. L. E. Simons *et al*., The responsive amygdala: Treatment-induced alterations in functional connectivity in pediatric complex regional pain syndrome. *Pain* **155,** 1727–1742 (2014). doi:10.1016/j.pain.2014.05.023 Medline

14. J. A. Hashmi, M. N. Baliki, L. Huang, A. T. Baria, S. Torbey, K. M. Hermann, T. J. Schnitzer, A. V. Apkarian, Shape shifting pain: Chronification of back pain shifts brain representation from nociceptive to emotional circuits. *Brain* **136**, 2751–2768 (2013). doi:10.1093/brain/awt211 Medline

15. F. Gore, E. C. Schwartz, B. C. Brangers, S. Aladi, J. M. Stujenske, E. Likhtik, M. J. Russo, J. A. Gordon, C. D. Salzman, R. Axel, Neural Representations of Unconditioned Stimuli in Basolateral Amygdala Mediate Innate and Learned Responses. *Cell* **162**, 134–145 (2015). doi:10.1016/j.cell.2015.06.027 Medline

16. V. Neugebauer, W. Li, G. C. Bird, G. Bhave, R. W. Gereau 4th, Synaptic plasticity in the amygdala in a model of arthritic pain: Differential roles of metabotropic glutamate receptors 1 and 5. *J. Neurosci.* **23**, 52–63 (2003). doi:10.1523/JNEUROSCI.23-01-00052.2003 Medline

17. S. Han, M. T. Soleiman, M. E. Soden, L. S. Zweifel, R. D. Palmiter, Elucidating an Affective Pain Circuit that Creates a Threat Memory. *Cell* **162**, 363–374 (2015). doi:10.1016/j.cell.2015.05.057 Medline

18. G. Ji, H. Sun, Y. Fu, Z. Li, M. Pais-Vieira, V. Galhardo, V. Neugebauer, Cognitive impairment in pain through amygdala-driven prefrontal cortical deactivation. *J. Neurosci.* **30**, 5451–5464 (2010). doi:10.1523/JNEUROSCI.0225-10.2010 Medline

19. B. F. Grewe, J. Gründemann, L. J. Kitch, J. A. Lecoq, J. G. Parker, J. D. Marshall, M. C. Larkin, P. E. Jercog, F. Grenier, J. Z. Li, A. Lüthi, M. J. Schnitzer, Neural ensemble dynamics underlying a long-term associative memory. *Nature* **543**, 670–675 (2017). doi:10.1038/nature21682 Medline

20. Y. Ziv, L. D. Burns, E. D. Cocker, E. O. Hamel, K. K. Ghosh, L. J. Kitch, A. El Gamal, M. J. Schnitzer, Long-term dynamics of CA1 hippocampal place codes. *Nat. Neurosci.* **16**, 264–266 (2013). doi:10.1038/nn.3329 Medline

21. J. G. Parker, J. D. Marshall, B. Ahanonu, Y.-W. Wu, T. H. Kim, B. F. Grewe, Y. Zhang, J. Z. Li, J. B. Ding, M. D. Ehlers, M. J. Schnitzer, Diametric neural ensemble dynamics in parkinsonian and dyskinetic states. *Nature* **557**, 177–182 (2018). doi:10.1038/s41586-018-0090-6 Medline

22. W. Zhang, D. M. Schneider, M. A. Belova, S. E. Morrison, J. J. Paton, C. D. Salzman, Functional circuits and anatomical distribution of response properties in the primate amygdala. *J. Neurosci.* **33**, 722–733 (2013). doi:10.1523/JNEUROSCI.2970-12.2013 Medline

23. P. Namburi, A. Beyeler, S. Yorozu, G. G. Calhoon, S. A. Halbert, R. Wichmann, S. S. Holden, K. L. Mertens, M. Anahtar, A. C. Felix-Ortiz, I. R. Wickersham, J. M. Gray, K. M. Tye, A circuit mechanism for differentiating positive and negative associations. *Nature* **520**, 675–678 (2015). doi:10.1038/nature14366 Medline

24. J. Kim, M. Pignatelli, S. Xu, S. Itohara, S. Tonegawa, Antagonistic negative and positive neurons of the basolaterial amygdala. *Nat. Neurosci.* **19**, 1636–1646 (2016). doi:10.1038/nn.4414 Medline

25. C. J. Guenthner, K. Miyamichi, H. H. Yang, H. C. Heller, L. Luo, Permanent genetic access to transiently active neurons via TRAP: Targeted recombination in active populations. *Neuron* **78**, 773–784 (2013). doi:10.1016/j.neuron.2013.03.025 Medline

26. B. L. Roth, DREADDs for Neuroscientists. *Neuron* **89**, 683–694 (2016). doi:10.1016/j.neuron.2016.01.040 Medline

27. J. L. Gomez, J. Bonaventura, W. Lesniak, W. B. Mathews, P. Sysa-Shah, L. A. Rodriguez, R. J. Ellis, C. T. Richie, B. K. Harvey, R. F. Dannals, M. G. Pomper, A. Bonci, M. Michaelides, Chemogenetics revealed: DREADD occupancy and activation via converted clozapine. *Science* **357**, 503–507 (2017). doi:10.1126/science.aan2475 Medline

28. G. G. Calhoon *et al*., Acute food deprivation rapidly modifies valence-coding microcircuits in the amygdala. *bioRxiv* 285189 [Preprint]. 19 March 2018. https://doi.org/10.1101/285189

29. C. A. von Hehn, R. Baron, C. J. Woolf, Deconstructing the neuropathic pain phenotype to reveal neural mechanisms. *Neuron* **73**, 638–652 (2012). doi:10.1016/j.neuron.2012.02.008 Medline

30. B. W. Balleine, S. Killcross, Parallel incentive processing: An integrated view of amygdala function. *Trends Neurosci.* **29**, 272–279 (2006). doi:10.1016/j.tins.2006.03.002 Medline

31. C. D. Salzman, S. Fusi, Emotion, cognition, and mental state representation in amygdala and prefrontal cortex. *Annu. Rev. Neurosci.* **33**, 173–202 (2010). doi:10.1146/annurev.neuro.051508.135256 Medline

32. J. E. LeDoux, R. Brown, A higher-order theory of emotional consciousness. *Proc. Natl. Acad. Sci. U.S.A.* **114**, E2016–E2025 (2017). doi:10.1073/pnas.1619316114 Medline

33. N. Grahek, *Feeling Pain and Being in Pain* (MIT Press, 2007).

34. R. Melzack, K. Casey, in *The Skin Senses* (Charles C. Thomas, 1968), pp. 432–439.

35. F. Ramirez, J. M. Moscarello, J. E. LeDoux, R. M. Sears, Active avoidance requires a serial basal amygdala to nucleus accumbens shell circuit. *J. Neurosci.* **35**, 3470–3477 (2015). doi:10.1523/JNEUROSCI.1331-14.2015 Medline

36. A. Burgos-Robles *et al*., Amygdala inputs to prefrontal cortex guide behavior amid conflicting cues of reward and punishment. *Nat. Neurosci.* **20,** 824–835 (2017). doi:10.1038/nn.4553 Medline

37. A. Beyeler, C.-J. Chang, M. Silvestre, C. Lévêque, P. Namburi, C. P. Wildes, K. M. Tye, Organization of Valence-Encoding and Projection-Defined Neurons in the Basolateral Amygdala. *Cell Reports* **22**, 905–918 (2018). doi:10.1016/j.celrep.2017.12.097 Medline

38. K. Wiech, Deconstructing the sensation of pain: The influence of cognitive processes on pain perception. *Science* **354**, 584–587 (2016). doi:10.1126/science.aaf8934 Medline

39. C. Torsney, A. B. MacDermott, Disinhibition opens the gate to pathological pain signaling in superficial neurokinin 1 receptor-expressing neurons in rat spinal cord. *J. Neurosci.* **26**, 1833–1843 (2006). doi:10.1523/JNEUROSCI.4584-05.2006 Medline

40. P. A. Pizzo, N. M. Clark, O. Carter Pokras, *Relieving Pain in America: A Blueprint for Transforming Prevention, Care, Education, and Research* (National Academies Press, 2011).

41. N. D. Volkow, F. S. Collins, The Role of Science in Addressing the Opioid Crisis. *N. Engl. J. Med.* **377**, 391–394 (2017). doi:10.1056/NEJMsr1706626 Medline

42. G. Corder, D. C. Castro, M. R. Bruchas, G. Scherrer, Endogenous and exogenous opioids in pain. *Annu. Rev. Neurosci.* **41,** 453–473 (2018). doi:10.1146/annurev-neuro-080317-061522 Medline

43. P. Skolnick, N. D. Volkow, Re-energizing the Development of Pain Therapeutics in Light of the Opioid Epidemic. *Neuron* **92**, 294–297 (2016). doi:10.1016/j.neuron.2016.09.051 Medline

44. F. Jelasić, Relation of the lateral part of the amygdala to pain. *Confin. Neurol.* **27**, 53–55 (1966). doi:10.1159/000103931 Medline

45. T. Grosser, C. J. Woolf, G. A. FitzGerald, Time for nonaddictive relief of pain. *Science* **355**, 1026–1027 (2017). doi:10.1126/science.aan0088 Medline

46. B. Ahanonu, calciumImagingAnalysis: a software package for analyzing one- and two-photon calcium imaging datasets. GitHub (2018); https://doi.org/10.5281/zenodo.2222295.

47. P. Jean-Richard-Dit-Bressel, S. Killcross, G. P. McNally, Behavioral and neurobiological mechanisms of punishment: Implications for psychiatric disorders. *Neuropsychopharmacology* **43**, 1639–1650 (2018). doi:10.1038/s41386-018-0047-3 Medline

48. R. Melzack, P. D. Wall, Pain mechanisms: A new theory. *Science* **150**, 971–979 (1965). doi:10.1126/science.150.3699.971 Medline

49. M. J. Millan, The induction of pain: An integrative review. *Prog. Neurobiol.* **57**, 1–164 (1999). doi:10.1016/S0301-0082(98)00048-3 Medline

50. G. Ekman, M. Frankenhaeuser, S. Levander, I. Mellis, The influence of intensity and duration of electrical stimulation on subjective variables. *Scand. J. Psychol.* **7**, 58–64 (1966). doi:10.1111/j.1467-9450.1966.tb01338.x Medline

51. T. Tashiro, A. Higashiyama, The perceptual properties of electrocutaneous stimulation: Sensory quality, subjective intensity, and intensity-duration relation. *Percept. Psychophys.* **30**, 579–586 (1981). doi:10.3758/BF03202013 Medline

52. M. S. Fanselow, The postshock activity burst. *Anim. Learn. Behav.* **10**, 448–454 (1982). doi:10.3758/BF03212284

53. H. Beaudry, I. Daou, A. R. Ase, A. Ribeiro-da-Silva, P. Séguéla, Distinct behavioral responses evoked by selective optogenetic stimulation of the major TRPV1+ and MrgD+ subsets of C-fibers. *Pain* **158**, 2329–2339 (2017). doi:10.1097/j.pain.0000000000001016 Medline

54. D. Terburg, D. Scheggia, R. Triana Del Rio, F. Klumpers, A. C. Ciobanu, B. Morgan, E. R. Montoya, P. A. Bos, G. Giobellina, E. H. van den Burg, B. de Gelder, D. J. Stein, R. Stoop, J. van Honk, The Basolateral Amygdala Is Essential for Rapid Escape: A Human and Rodent Study. *Cell* **175**, 723–735.e16 (2018). doi:10.1016/j.cell.2018.09.028 Medline

55. J. P. Fadok, S. Krabbe, M. Markovic, J. Courtin, C. Xu, L. Massi, P. Botta, K. Bylund, C. Müller, A. Kovacevic, P. Tovote, A. Lüthi, A competitive inhibitory circuit for selection

of active and passive fear responses. *Nature* **542**, 96–100 (2017). doi:10.1038/nature21047 Medline

56. C. Gauriau, J. F. Bernard, Pain pathways and parabrachial circuits in the rat. *Exp. Physiol.* **87**, 251–258 (2002). doi:10.1113/eph8702357 Medline

57. M. E. Carter, M. E. Soden, L. S. Zweifel, R. D. Palmiter, Genetic identification of a neural circuit that suppresses appetite. *Nature* **503**, 111–114 (2013). doi:10.1038/nature12596 Medline

58. R. D. Palmiter, The Parabrachial Nucleus: CGRP Neurons Function as a General Alarm. *Trends Neurosci.* **41**, 280–293 (2018). doi:10.1016/j.tins.2018.03.007 Medline

59. C. A. Campos, A. J. Bowen, C. W. Roman, R. D. Palmiter, Encoding of danger by parabrachial CGRP neurons. *Nature* **555**, 617–622 (2018). doi:10.1038/nature25511 Medline

60. P. Tovote, M. S. Esposito, P. Botta, F. Chaudun, J. P. Fadok, M. Markovic, S. B. E. Wolff, C. Ramakrishnan, L. Fenno, K. Deisseroth, C. Herry, S. Arber, A. Lüthi, Midbrain circuits for defensive behaviour. *Nature* **534**, 206–212 (2016). doi:10.1038/nature17996 Medline

61. T. Ozawa, E. A. Ycu, A. Kumar, L.-F. Yeh, T. Ahmed, J. Koivumaa, J. P. Johansen, A feedback neural circuit for calibrating aversive memory strength. *Nat. Neurosci.* **20**, 90–97 (2017). doi:10.1038/nn.4439 Medline

62. R. J. Fox, C. A. Sorenson, Bilateral lesions of the amygdala attenuate analgesia induced by diverse environmental challenges. *Brain Res.* **648**, 215–221 (1994). doi:10.1016/0006-8993(94)91120-7 Medline

63. W. Zhu, Z. Z. Pan, µ-Opioid-mediated inhibition of glutamate synaptic transmission in rat central amygdala neurons. *Neuroscience* **133**, 97–103 (2005). doi:10.1016/j.neuroscience.2005.02.004 Medline

64. M. A. Oliveira, W. A. Prado, Role of PAG in the antinociception evoked from the medial or central amygdala in rats. *Brain Res. Bull.* **54**, 55–63 (2001). doi:10.1016/S0361-9230(00)00420-2 Medline

65. J. Annese, N. M. Schenker-Ahmed, H. Bartsch, P. Maechler, C. Sheh, N. Thomas, J. Kayano, A. Ghatan, N. Bresler, M. P. Frosch, R. Klaming, S. Corkin, Postmortem examination of patient H.M.'s brain based on histological sectioning and digital 3D reconstruction. *Nat. Commun.* **5**, 3122 (2014). doi:10.1038/ncomms4122 Medline

66. P. Veinante, I. Yalcin, M. Barrot, The amygdala between sensation and affect: A role in pain. *J. Mol. Psychiatry* **1**, 9 (2013). doi:10.1186/2049-9256-1-9 Medline

67. P. Kyriazi, D. B. Headley, D. Pare, Multi-dimensional Coding by Basolateral Amygdala Neurons. *Neuron* **99**, 1315–1328.e5 (2018). doi:10.1016/j.neuron.2018.07.036 Medline

68. T.-W. Chen, T. J. Wardill, Y. Sun, S. R. Pulver, S. L. Renninger, A. Baohan, E. R. Schreiter, R. A. Kerr, M. B. Orger, V. Jayaraman, L. L. Looger, K. Svoboda, D. S. Kim, Ultrasensitive fluorescent proteins for imaging neuronal activity. *Nature* **499**, 295–300 (2013). doi:10.1038/nature12354 Medline

69. B. Slotnick, A simple 2-transistor touch or lick detector circuit. *J. Exp. Anal. Behav.* **91**, 253–255 (2009). doi:10.1901/jeab.2009.91-253 Medline

70. B. Solway, S. C. Bose, G. Corder, R. R. Donahue, B. K. Taylor, Tonic inhibition of chronic pain by neuropeptide Y. *Proc. Natl. Acad. Sci. U.S.A.* **108**, 7224–7229 (2011). doi:10.1073/pnas.1017719108 Medline

71. K. Kobayakawa, R. Kobayakawa, H. Matsumoto, Y. Oka, T. Imai, M. Ikawa, M. Okabe, T. Ikeda, S. Itohara, T. Kikusui, K. Mori, H. Sakano, Innate versus learned odour processing in the mouse olfactory bulb. *Nature* **450**, 503–508 (2007). doi:10.1038/nature06281 Medline

72. C. M. Root, C. A. Denny, R. Hen, R. Axel, The participation of cortical amygdala in innate, odour-driven behaviour. *Nature* **515**, 269–273 (2014). doi:10.1038/nature13897 Medline

73. L. R. Saraiva, K. Kondoh, X. Ye, K. H. Yoon, M. Hernandez, L. B. Buck, Combinatorial effects of odorants on mouse behavior. *Proc. Natl. Acad. Sci. U.S.A.* **113**, E3300–E3306 (2016). doi:10.1073/pnas.1605973113 Medline

74. Y. A. Blednov, B. F. Cravatt, S. L. Boehm 2nd, D. Walker, R. A. Harris, Role of endocannabinoids in alcohol consumption and intoxication: Studies of mice lacking fatty acid amide hydrolase. *Neuropsychopharmacology* **32**, 1570–1582 (2007). doi:10.1038/sj.npp.1301274 Medline

75. P. Thévenaz, U. E. Ruttimann, M. Unser, A pyramid approach to subpixel registration based on intensity. *IEEE Trans. Image Process.* **7**, 27–41 (1998). doi:10.1109/83.650848 Medline

76. E. A. Mukamel, A. Nimmerjahn, M. J. Schnitzer, Automated analysis of cellular signals from large-scale calcium imaging data. *Neuron* **63**, 747–760 (2009). doi:10.1016/j.neuron.2009.08.009 Medline

77. Y. Benjamini, Y. Hochberg, Controlling the False Discovery Rate: A Practical and Powerful Approach to Multiple Testing. *J. R. Stat. Soc. B* **57**, 289–300 (1995). doi:10.1111/j.2517-6161.1995.tb02031.x

78. M. J. Mathie, N. H. Lovell, A. C. F. Coster, B. G. Celler, *Proc. Second Jt. 24th Annu. Conf. Annu. Fall Meet. Biomed. Eng. Soc.*, pp. 2481–2482 (2002).

79. Y. Li, A. Mathis, B. F. Grewe, J. A. Osterhout, B. Ahanonu, M. J. Schnitzer, V. N. Murthy, C. Dulac, Neuronal Representation of Social Information in the Medial Amygdala of Awake Behaving Mice. *Cell* **171**, 1176–1190.e17 (2017). doi:10.1016/j.cell.2017.10.015 Medline

80. S. M. Iyer, K. L. Montgomery, C. Towne, S. Y. Lee, C. Ramakrishnan, K. Deisseroth, S. L. Delp, Virally mediated optogenetic excitation and inhibition of pain in freely moving nontransgenic mice. *Nat. Biotechnol.* **32**, 274–278 (2014). doi:10.1038/nbt.2834 Medline

81. G. Corder, V. L. Tawfik, D. Wang, E. I. Sypek, S. A. Low, J. R. Dickinson, C. Sotoudeh, J. D. Clark, B. A. Barres, C. J. Bohlen, G. Scherrer, Loss of μ opioid receptor signaling in nociceptors, but not microglia, abrogates morphine tolerance without disrupting analgesia. *Nat. Med.* **23**, 164–173 (2017). doi:10.1038/nm.4262 Medline

82. A. Manglik *et al*., Structure-based discovery of opioid analgesics with reduced side effects. *Nature* **537**, 185–190 (2016).

83. C. J. Woolf, Long term alterations in the excitability of the flexion reflex produced by peripheral tissue injury in the chronic decerebrate rat. *Pain* **18**, 325–343 (1984). doi:10.1016/0304-3959(84)90045-9 Medline

84. R. J. Blanchard, D. C. Blanchard, Passive and active reactions to fear-eliciting stimuli. *J. Comp. Physiol. Psychol.* **68**, 129–135 (1969). doi:10.1037/h0027676 Medline

85. R. Bolles, Species-specific defense reactions and avoidance learning. *Psychol. Rev.* **77**, 32–48 (1970). doi:10.1037/h0028589

86. R. Bolles, M. Fanselow, A perceptual-defensive-recuperative model of fear and pain. *Behav. Brain Sci.* **3**, 291–323 (1980). doi:10.1017/S0140525X0000491X

87. C. Darwin, *The Expression of the Emotions in Man and Animals* (Albemarle, London, 1872).

88. W. K. Estes, Discriminative conditioning; effects of a Pavlovian conditioned stimulus upon a subsequently established operant response. *J. Exp. Psychol.* **38**, 173–177 (1948). doi:10.1037/h0057525 Medline

89. W. Estes, B. Skinner, Some quantitative properties of anxiety. *J. Exp. Psychol.* **29**, 390–400 (1941). doi:10.1037/h0062283

90. J. S. Mogil, Animal models of pain: Progress and challenges. *Nat. Rev. Neurosci.* **10**, 283–294 (2009). doi:10.1038/nrn2606 Medline

91. R. A. Rescorla, Pavlovian conditioned fear in Sidman avoidance learning. *J. Comp. Physiol. Psychol.* **65**, 55–60 (1968). doi:10.1037/h0025412 Medline

92. B. Skinner, *Appleton-CenturyCrofts, New York* (1938).

93. S. R. Chaplan, F. W. Bach, J. W. Pogrel, J. M. Chung, T. L. Yaksh, Quantitative assessment of tactile allodynia in the rat paw. *J. Neurosci. Methods* **53**, 55–63 (1994). doi:10.1016/0165-0270(94)90144-9 Medline

94. G. Corder, S. Doolen, R. R. Donahue, M. K. Winter, B. L. Jutras, Y. He, X. Hu, J. S. Wieskopf, J. S. Mogil, D. R. Storm, Z. J. Wang, K. E. McCarson, B. K. Taylor, Constitutive μ-opioid receptor activity leads to long-term endogenous analgesia and dependence. *Science* **341**, 1394–1399 (2013). doi:10.1126/science.1239403 Medline
